# Skull base repair following endonasal pituitary and skull base tumour resection: a systematic review

**DOI:** 10.1007/s11102-021-01145-4

**Published:** 2021-05-10

**Authors:** Danyal Z. Khan, Ahmad M. S. Ali, Chan Hee Koh, Neil L. Dorward, Joan Grieve, Hugo Layard Horsfall, William Muirhead, Thomas Santarius, Wouter R. Van Furth, Amir H. Zamanipoor Najafabadi, Hani J. Marcus

**Affiliations:** 1 Division of Neurosurgery, National Hospital for Neurology and Neurosurgery, Queen Square, London, UK; 2 Wellcome/EPSRC Centre for Interventional and Surgical Sciences, University College London, London, UK; 3 Department of Neurosurgery, The Walton Centre, Liverpool, UK; 4 Division of Neurosurgery, University of Cambridge and Cambridge University Hospitals, Cambridge, UK; 5 Department of Neurosurgery, University Neurosurgical Centre Holland, Leiden University Medical Centre, Haaglanden Medical Centre and Haga Teaching Hospital, Leiden and The Hague, The Netherlands

**Keywords:** Endoscopic transsphenoidal surgery, Endoscopic endonasal, Skull base surgery, Cerebrospinal fluid, CSF, Cerebrospinal fluid leak, Cerebrospinal fluid rhinorrhoea

## Abstract

**Purpose:**

Postoperative cerebrospinal fluid rhinorrhoea (CSFR) remains a frequent complication of endonasal approaches to pituitary and skull base tumours. Watertight skull base reconstruction is important in preventing CSFR. We sought to systematically review the current literature of available skull base repair techniques.

**Methods:**

Pubmed and Embase databases were searched for studies (2000–2020) that (a) reported on the endonasal resection of pituitary and skull base tumours, (b) focussed on skull base repair techniques and/or postoperative CSFR risk factors, and (c) included CSFR data. Roles, advantages and disadvantages of each repair method were detailed. Random-effects meta-analyses were performed where possible.

**Results:**

193 studies were included. Repair methods were categorised based on function and anatomical level. There was absolute heterogeneity in repair methods used, with no independent studies sharing the same repair protocol. Techniques most commonly used for low CSFR risk cases were fat grafts, fascia lata grafts and synthetic grafts. For cases with higher CSFR risk, multilayer regimes were utilized with vascularized flaps, gasket sealing and lumbar drains. Lumbar drain use for high CSFR risk cases was supported by a randomised study (Oxford CEBM: Grade B recommendation), but otherwise there was limited high-level evidence. Pooled CSFR incidence by approach was 3.7% (CI 3–4.5%) for transsphenoidal, 9% (CI 7.2–11.3%) for expanded endonasal, and 5.3% (CI 3.4–7%) for studies describing both. Further meaningful meta-analyses of repair methods were not performed due to significant repair protocol heterogeneity.

**Conclusions:**

Modern reconstructive protocols are heterogeneous and there is limited evidence to suggest the optimal repair technique after pituitary and skull base tumour resection. Further studies are needed to guide practice.

**Supplementary Information:**

The online version contains supplementary material available at 10.1007/s11102-021-01145-4.

## Background

Endonasal approaches to the skull base, most commonly described in the transsphenoidal approach (TSA) to pituitary lesions, have allowed minimally invasive and maximally effective surgical resection of skull base tumours. They may allow early optic decompression whilst avoiding excessive vascular manipulation, resulting in superior visual outcomes compared to transcranial approaches [1–3]. As these techniques have developed, access to the skull base has been bolstered, establishing the expanded endoscopic endonasal approaches (EEA)—allowing resection of larger pituitary lesions and an increasing variety of skull base tumours beyond the sella alone [4, 5].

Despite the purported advantages of endonasal approaches (TSA and EEA), postoperative cerebrospinal fluid rhinorrhoea (CSFR) remains a frequent complication, which may result in significant complications, including meningitis, pneumocephalus and the need for reoperation [6–8]. Reported CSFR rates are variable in the literature—generally up to 5% for TSA and up to 20% for EEA [7, 9, 10].

CSFR results from iatrogenic disruption of the barrier between the CSF-containing subarachnoid space and the sinonasal cavity during surgery. This disruption may be unavoidable (e.g. intradural tumour resection) or inadvertent (e.g. most pituitary adenoma resections). Regardless of the cause, a watertight repair of the skull base is paramount in preventing postoperative CSFR [11]. This is a technically challenging task—using long rigid instruments to repair a defect against gravity and under dependent intracranial structures [12]. There are various available repair options with varying morbidity profiles, including reconstructive materials (e.g. fat grafts, nasoseptal flaps) and supportive measures (e.g. lumbar drains) [11, 13]. These repair choices may be influenced by numerous factors, including approach (TSA or EEA), presence or grade of intraoperative CSF leak (ioCSFL) [14], patient characteristics (e.g. elevated BMI) and surgeon experience [11, 15, 16]. However, there is a paucity of high-quality evidence or consensus on skull base repair methodology, and surgical practice is resultantly heterogenous [11, 13, 16].

The first step in establishing optimal skull base repair techniques after endonasal resection of pituitary and skull base tumours is understanding the current scope of techniques available. Although there are several studies that review this topic, none are both systematic and comprehensive [11, 17–23]. We therefore sought to systematically review the current literature and produce a framework of available skull base repair techniques, their potential roles, advantages and disadvantages.

## Methods

### Search strategy

This review was conducted in accordance with the Preferred Reporting Items for Systematic Reviews and Meta-Analyses (PRISMA) Statement [24] with the study protocol published a priori in an open-access database (PROSPERO ID: 42020172372). A search strategy was created using the keywords “transsphenoidal”, “endonasal”, “EEA”, “skull base”, “cerebrospinal fluid” and synonyms (Supplementary information 1). Studies from 2000 to 2020 were included if they: (a) reported on resection of pituitary and skull base tumours via TSA/EEA, (b) focussed primarily on skull base repair techniques and/or postoperative CSFR risk factors, and (c) included the incidence of postoperative CSF rhinorrhoea. Exclusion criteria were: spontaneous/traumatic CSF rhinorrhoea, paediatrics (< 16 years old), case series < 3 patients, editorials, secondary research, animal studies and cadaveric studies. Studies without a specific focus on skull base repair or postoperative rhinorrhoea risk factors were excluded. Both PubMed and Embase databases were searched on 19/06/2020. Duplicates were removed using Endnote X9. Independent abstract screening was performed in duplicate by two authors (DZK, AMSA). Related-article search was performed for each included article. Review of full-text articles ensued, according to the inclusion/exclusion criteria. Any discrepancies in selection were resolved by discussion and mutual agreement.

### Data extraction

Data points extracted from the included articles comprised of: study details (continent, design), tumour characteristics (sample size, tumour type), operative characteristics (surgical approach, intraoperative CSF leak and grade [14], skull base repair materials used and rationale behind choice; CSF diversion use), complications (CSFR incidence/method of confirmation/number requiring re-operation; repair-related complications).

### Quality assessment

A bespoke risk of bias tool (based on COSMOS-E guidelines) [25] was created for study-level assessment focusing on information bias and selection bias (Supplementary information 2). This tool sought to interrogate key study characteristics included sample, ioCSFL, skull base repair and postoperative CSFR. Each study was scored out of 5, stratifying studies into low (score 0–1), moderate (score 2–3) and high (score 4–5) risk of bias. This was a deviation from our protocol, after use of generic assessment tools was felt not to clearly delineate study quality. Additionally, after categorisation of repair methods, each category was assigned a grade of recommendation based on the 2009 Oxford Levels of Evidence Criteria [26].

### Data analysis

Repair techniques were organised into a comprehensive taxonomy according to uniting characteristics (e.g. intended function, anatomical level, material type) [27, 28]. Each category was explored in turn in terms of sub-categories, indications, advantages, disadvantages and refinements. Summary statistics (using Excel, Version 16.43, Microsoft) were generated for the number of studies and cases by pathology, approach, and repair technique. Random-effects meta-analyses (using R “metafor” package, version 3.6.1, R Foundation, Austria) were also performed for the CSFR rates by approach and repair technique where possible. Study heterogeneity was assessed by calculating I^2^ values (I^2^ > 50% considered significant).

## Results

### General

**Fig. 1:**
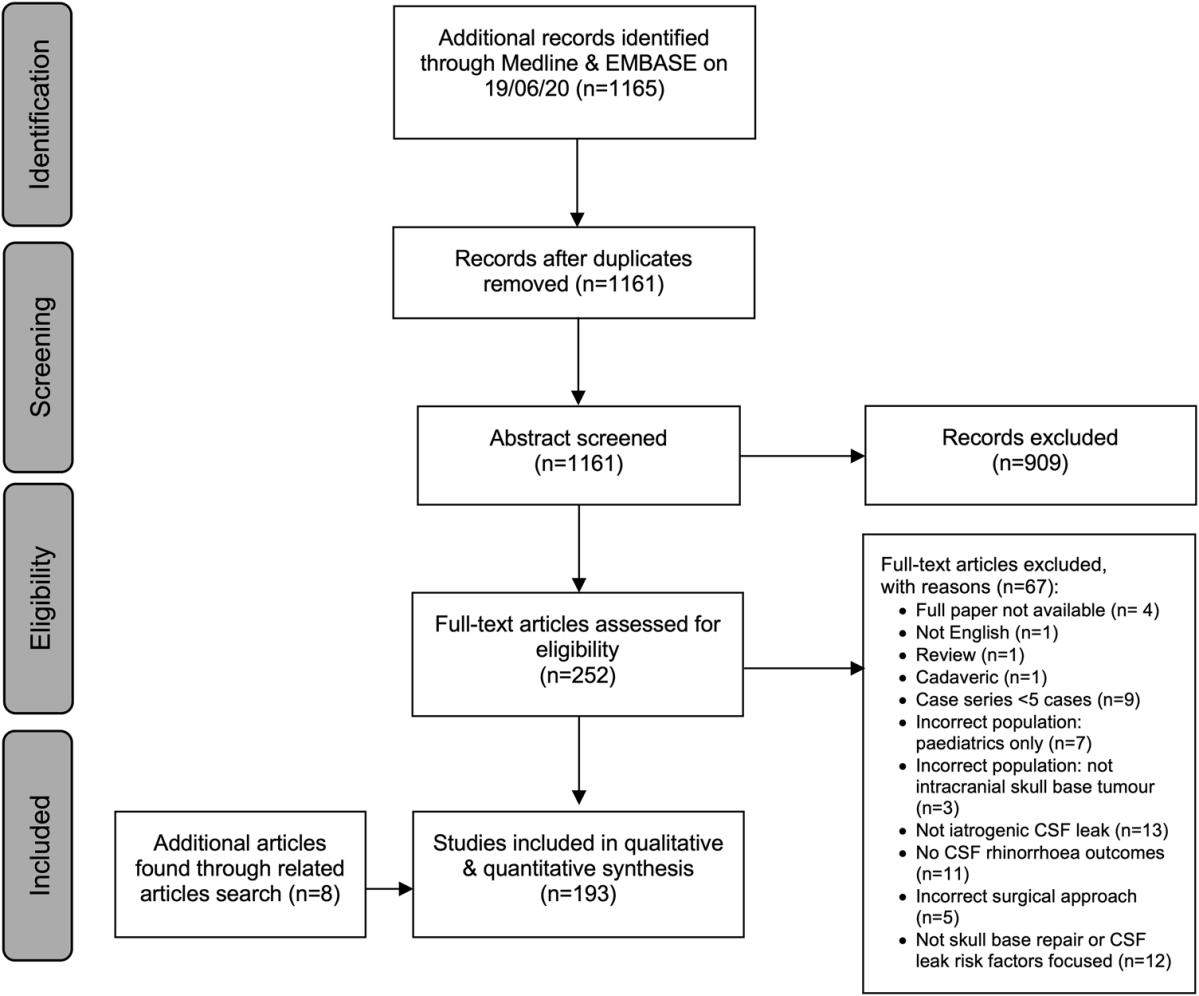
PRISMA flow chart of paper identification, screening and eventual inclusion

The search returned 1165 records (1161 after removal of duplicates). After abstract screening, 256 full-text studies were reviewed with 193 studies included for final analysis (Fig. 1). The yearly rate of publication in this field has been increasing over time (Supplementary information 3). Most studies originated from groups in North America (44.6%, 86/193), Asia (32.6%, 63/193) and Europe (18.1%, 35/193). The majority (191/193) of studies were case series (prospective or retrospective) and 2/193 were randomised controlled trials (RCT). The median risk of bias score of 2 (IQR 1–3) suggestive of moderate risk of bias (Supplementary information 2). Of the included studies, the reported approaches were: TSA (49.2%, 95/193), EEA (28.5%, 55/193), or both (22.3%, 43/193). The most frequent pathologies are highlighted in Table 1—reported at study level only as many studies did not report frequency for each pathology included.

**Table 1:** Commonest pathologies treated using the transsphenoidal or expanded endonasal approach

Most common pathology types	No. of studies
Pituitary adenoma	156
Rathke Cleft Cyst	65
Craniopharyngioma	92
Chordoma	56
Meningioma (e.g. planum sphenoidale, tuberculum sellae, clival, cavernous, olfactory groove)	77
Arachnoid cyst	14
Metastatic (e.g. breast, renal, melanoma)	17
Other cysts (epidermoid, dermoid, colloid, hydatid)	14

Other pathologies included	Examples
Central nervous tissue	Esthesioneuroblastoma
	Pituicytoma
	Pituitary apoplexy
	Schwannoma
	Optic glioma
	Pilocytic astrocytoma
	Hypothalamic hamartoma
Connective tissue	Chondrosarcoma
	Osteosarcoma
	Sarcoma
Vascular	Hemangioblastoma
	Cavernoma
Haematological	Lymphoma
	Plasmacytoma
Miscellaneous	Germinoma
	Glomus jugulare
	Granular cell tumour
	Xanthogranuloma
	Mucocele
	Papilloma
	Cystic adenocarcinoma

### Repair techniques

Numerous materials and techniques were reported within the included studies. There was almost absolute heterogeneity, with no studies (from different author groups) sharing the same repair protocol (Supplementary information 4).

The choice of the number of layers and the type of repair was often decided based on pre- or intra-operative considerations. A predominant consideration was the presence and severity of ioCSFL [14, 29–34], with the Esposito-Kelly grading system serving as a basis for many repair protocols [14]. Patient-related factors considered in planning repair strategy included age (e.g. poor wound healing in the elderly), elevated body mass index, previous endonasal surgery and concomitant radiotherapy [10, 28, 35–41]. Tumour-related factors included pathology type [28, 31, 33, 35, 42, 43], size, extension (e.g. suprasellar extension) [31] and consistency [43]. Operative factors consisted of the presence of intrasellar dead space [14, 44], dural morphology (thinned or tense) [31, 45] and osteodural defect size [33, 46, 47]. When considering osteodural defect location, transplanum (where the exposed optic chiasm cannot provide counter pressure to support the repair) and clival (vertical plane and are often large/high flow) defects were considered particularly high risk and required robust repair [28, 37, 46–49].

**Fig. 2:**
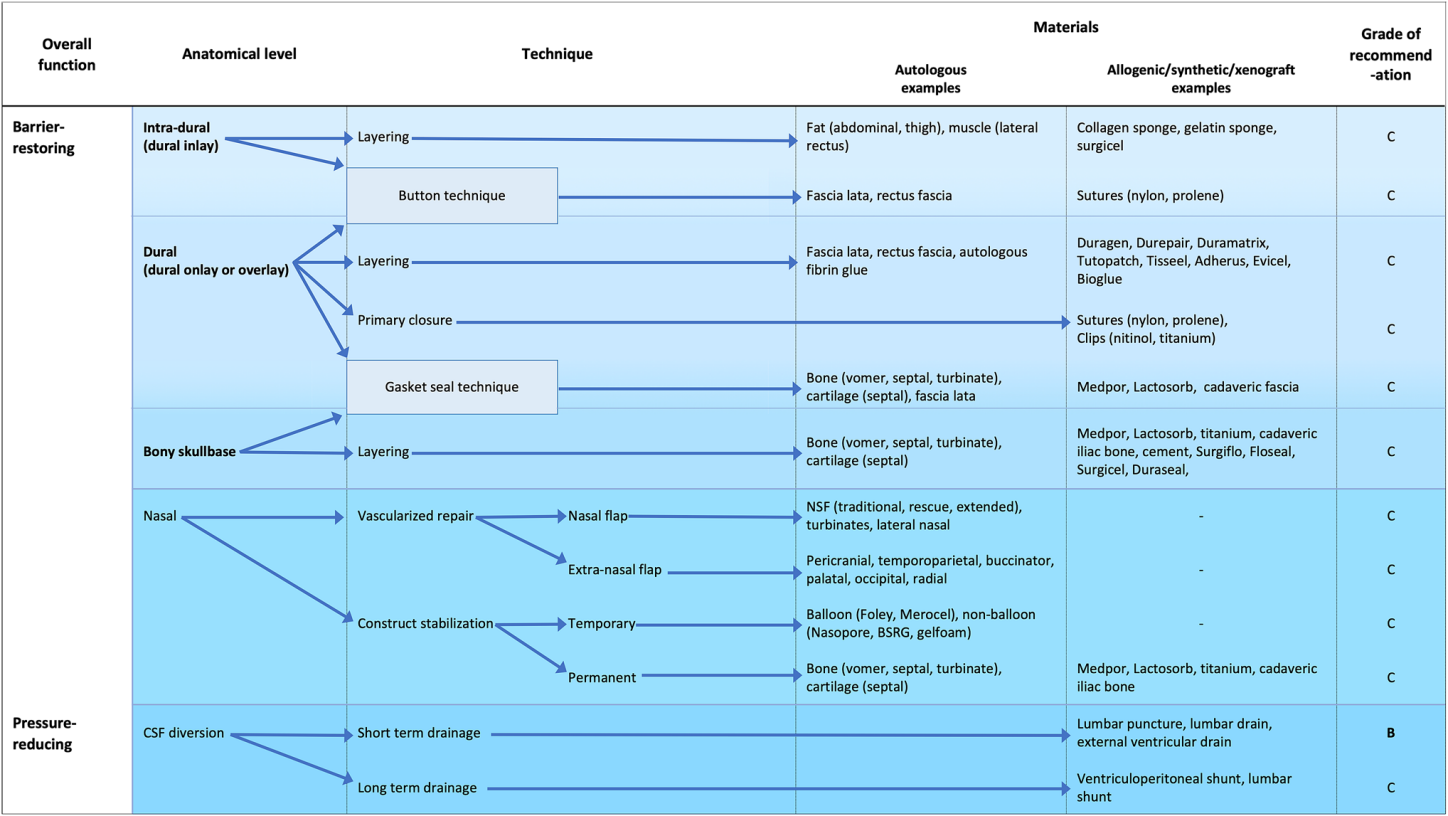
Repair technique taxonomy

**Fig. 3:**
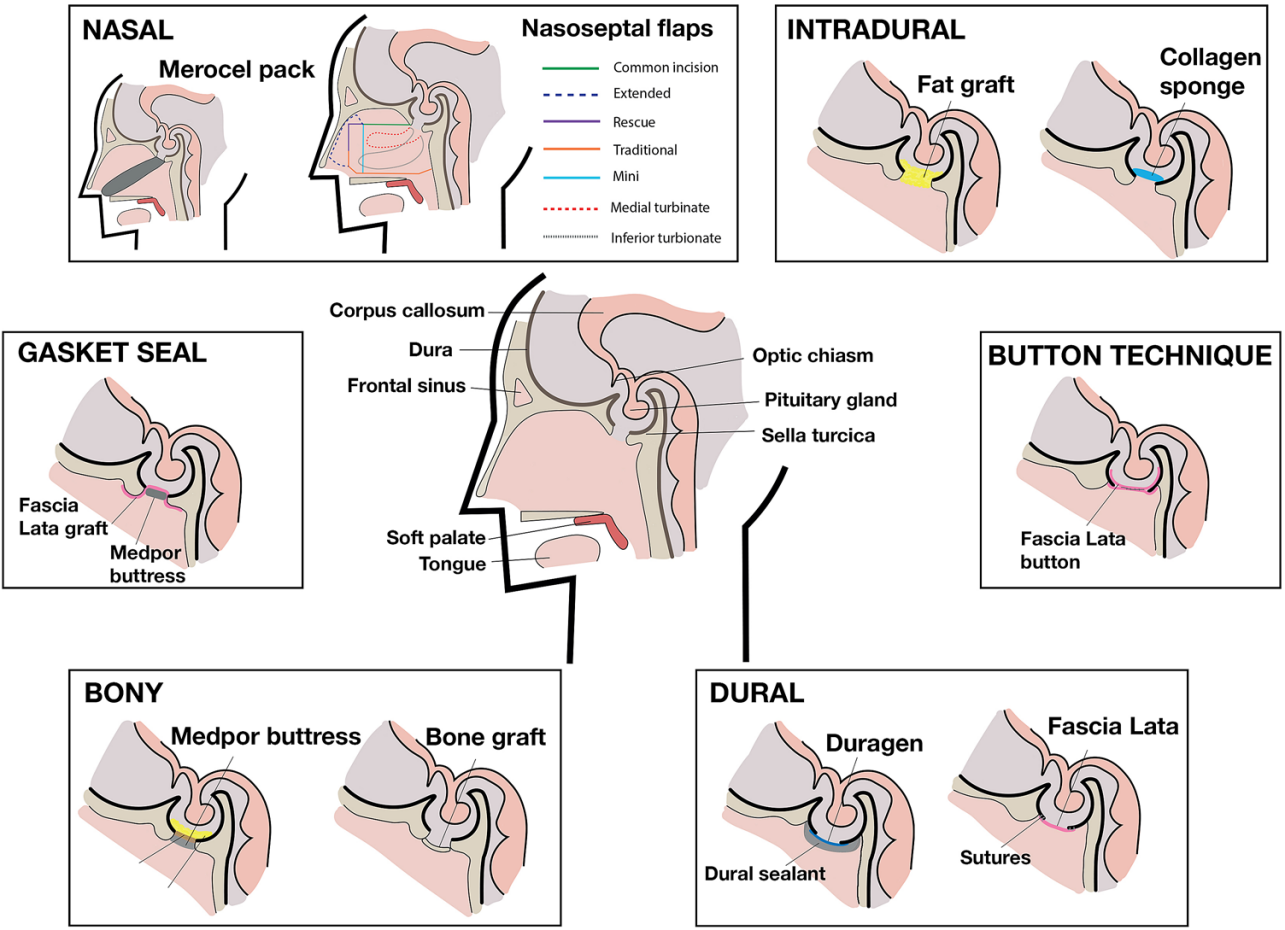
Sagittal section to the skull base highlighting various levels of repair and common repair techniques used per level

Below, we discuss these techniques in turn according to our taxonomy (Figs. 2, 3). Table 2 discusses the purposes, advantages and disadvantages at each anatomical phase. The incidences of each of these repair materials across TSA and EEA papers are highlighted in Supplementary Information 5.

**Table 2:** Overview of the repair technique characteristics within each anatomical level

Purpose	Examples	Advantages	Disadvantages
Intradural			
To obliterate excessive dead space (e.g. intrasellar) after tumour resection	• Autologous grafts (e.g. fat) • Synthetic grafts (e.g. collagen sponge, gelatine sponge)	• Provides support to surrounding neurovascular structures (e.g. optic chiasm) • Absorbs CSF pulsations (if fluctuant)	• Risk of overpacking causing and damage to surrounding structures (e.g. optic apparatus compression) • Risk of obstructive hydrocephalus if particulate entry into ventricular system (e.g. an open third ventricle) • Premature absorption (fat grafts) • Potential interference with postoperative MRI interpretation • Autologous grafts: donor-site morbidity • Synthetic grafts: increased direct cost, decreased biocompatibility, infection
Dural			
To repair the dural defect—via primary closure or reconstruction	• Dural sutures • Dural clips • Autologous grafts (e.g. fascia lata) • Allografts and xenografts (e.g. cadaveric fascia) • Synthetic grafts (e.g. artificial dura)	• Potential for watertight seal	• Sutures/clips: technically challenging, time-consuming nature, steep learning curves, limited to small defects unless grafts used in patchwork fashion, radiological artefacts (clips) • Autologous grafts: donor-site morbidity • Allografts and xenografts: donor infection transmission • Synthetic grafts: increased direct cost, decreased biocompatibility, infection
Bony			
To repair the bony skull base (e.g. sellar floor) using rigid or semi-rigid grafts	• Autologous grafts (e.g. nasal bone) • Allografts and xenografts (e.g. cadaveric iliac crest) • Synthetic grafts (e.g. Medpor)	• Capable of withstanding higher forces across the repair site and provides protection to underlying structures	• Autologous grafts: donor-site morbidity • Allografts and xenografts: donor infection transmission • Synthetic grafts: increased direct cost, decreased biocompatibility, infection, radiological artefacts
Nasal			
Direct reconstruction of skull base (vascular flaps) or support to underlying repair (buttresses and nasal packing)	• Pedicled flap • Autologous buttress • Synthetic buttress • Nasal packing	• Vascularised repairs have the potential to maintain supportive strength for a long period of time and adhere strongly to the skull base • Packing and buttressing may protect the underlying repair from force across the repair site	• Vascular flaps: donor site morbidity (e.g. nasal crusting) • Autologous buttress: donor site morbidity • Synthetic buttress: increased direct cost, decreased biocompatibility, infection, extrusion, chronic sphenoid sinusitis, radiological artefacts • Packing: discomfort, risk of dislodgement, risk of adhesion to underlying repair
CSF diversion			
Reduction of CSF pressure and pulsations to support reconstruction	• Short-term (e.g. lumbar drain) • Long-term (e.g. ventriculoperitoneal shunt)	• Supported by level 1B evidence (peri-operative lumbar drainage)	• Low-pressure headaches, pneumocephalus, infection, neural injury, bleeding, catheter dislodgment, retained catheter, increased length of stay, decreased mobility, venous thromboembolism, anaesthetic complications

*CSF* cerebrospinal fluid, *MRI* magnetic resonance imaging

#### Barrier restoring: intradural phase (dural inlay)

Materials used were autologous or synthetic (Figs. 2, 3). The most common autologous graft was composed of fat (Table 2) from the abdomen or thigh (137 studies) [50–57]. Techniques to mitigate the potential disadvantages (Table 3) of this material included robust dural repair underlying the fat graft with clips [51] or sutures to protect neurovascular structures from overpacking [58]. Dislodgement can be prevented by supporting the graft with a rigid buttress or suturing the graft to the dura (“bath-plug technique”) [32, 51]. Premature absorption can be prevented by placing a barrier graft between fat tissue and overlying vascularised flaps [52], whilst harvesting the fat via an intra-umbilical approach may reduce donor site scarring and morbidity [55]. Additionally, wrapping the fat tissue in barrier grafts (e.g. Avitene or oxidised cellulose) may aid placement and stability (prevents lobules detaching) [56, 59]. Alternatively, synthetic grafts can be used for the intra-dural phase—either layered thin grafts (e.g. Alloderm, Tachosil) or sponge-like voluminous grafts (collagen sponges, gelatin sponges) [15, 60–62]. Several studies have found similar CSFR results between synthetic and fat sellar packing packing [60, 62, 63], although some authors suggested fat graft packing was more effective in the context of moderate/large flow ioCSFL [15].

**Table 3:** The rates and methods of confirmation and management of intraoperative CSF leak and postoperative CSF rhinorrhoea

Measure	Transsphenoidal approach	Expanded endonasal approach	Both approaches
Intraoperative CSF leak			
No. of studies reporting	87/95 (92%)	53/55 (97%)	32/43 (82%)
Methods of confirmation (number of studies)	Valsalva (n = 32) IT fluorescein (n = 6) Observation alone (n = 2) IT saline (n = 1) Not specified (n = 54)	Valsalva (n = 3) IT fluorescein (n = 5) Observation alone (n = 2) Not specified (n = 45)	Valsalva (n = 12) IT fluorescein (n = 4) Observation alone (n = 2) Not specified (n = 25)
Grading methods (number of studies)	Esposito-Kelly (n = 15) High/low flow (n = 13) Modified Esposito-Kelly (n = 1) Anatomical grading (n = 2) Not specified (n = 64)	Esposito-Kelly (n = 4) High/low flow (n = 12) Not specified (n = 43)	Esposito-Kelly (n = 13) High/low flow (n = 12) Modified Esposito-Kelly (n = 2) Not specified (n = 16)
Postoperative CSF rhinorrhea			
No. of studies reporting	94/95 (99%)	55/55 (100%)	41/43 (95%)
Adjuncts for confirmation (number of studies)	β2 transferrin (n = 10) tes-tape (n = 1) Not specified (n = 84)	Pneumocephalus on CT (n = 2) β2 transferrin (n = 3) Not specified (n = 50)	β2 transferrin (n = 4) Pneumocephalus on CT (n = 5) MRI (n = 3) Leaning forward (n = 4) Tch99 cisternography (n = 1) IT fluorescein (n = 1) Endoscopic exploration (n = 2) Not specified (n = 23
CSFR management methods (number of studies)	Lumbar drain (n = 28) Reoperation (n = 46) VPS (n = 2) Combined lumbar drain and reoperation (n = 17) Serial lumbar punctures (n = 1) Not specified (n = 1)	Lumbar drain (n = 18) Reoperation (n = 30) VPS (n = 3) EVD (n = 1) Not specified (n = 3)	Lumbar drain (n = 15) Reoperation (n = 24) Combined lumbar drain and reoperation (n = 4) Not specified (n = 8)

*IT* intrathecal, *Tch* technetium, *CSF* cerebrospinal fluid, *MRI* magnetic resonance imaging, *CT* computed topography, *VPS* ventriculo-peritoneal shunt, *EVD* external ventricualr drain

#### Barrier restoring: dural (dural onlay and overlay)

Direct closure using suturing (Figs. 2, 3) was reported in 69 papers [58, 64]. A variety of suture materials were used (e.g. 7–0 pronova [65], 5–0 nylon [66], 5–0 PDS [58]) in a simple or continuous fashion. Some authors preferred continuous suturing (particularly for high flow ioCSFL and large dural defects) due to even tension distribution, the potential for a tighter seal across the defect, and the requirement of only two knots [42, 67]. To offset some of the challenges of this technique (Table 2) [42, 64, 65, 67–69], surgeons describe suturing grafts (fat, fascia, gelatin sponge) directly to the dura in a patchwork configuration for larger defects [38, 58, 64–70, 143]. By using specialised suture-tying instruments with a sliding-lock-knot technique, suturing was increasingly feasible [58, 65, 66, 68]. Other modifications include the “snare technique”: catching redundant dura around an identified leaking point with a loop of suture—sealing the leak without additional puncturing [71]. Similarly, direct dural closure utilising clips (with or without graft patches) is non-penetrating, quicker and less technically demanding than suturing [52, 72–75]. Using particular materials (e.g. Nitinol [52, 75]) created less radiological artefact than traditional titanium clips [72–74].

Alternatively, dural reconstruction through the use of grafts alone was described in 64 papers. These can be autografts (e.g. fascia lata, septal mucosa), synthetic (e.g. Duramatrix, Duragen), allografts (e.g. cadaveric fascia) or xenografts (e.g. equine pericardium) [41, 76–78]. Autologous options are generally cheaper, universally available and maximally biocompatible [29, 78, 79]. Fascia lata grafts represent an established, strong, versatile and pliable autologous material which is easy to harvest [44, 78]. Alternatives include nasal mucosa grafts (e.g. septal or turbinate) [80–83] and leukocyte-enriched platelet-rich fibrin membranes harvested from 10 to 20 ml of the patient’s blood—both of which avoid a separate abdominal or thigh incision [84–86]. Similarly, autologous dura may be used to reconstruct defects by forming a dural flap at the time of durotomy which is subsequently replaced during reconstruction [87]. In terms of synthetic grafts, many are collagen-based and sheet-like (e.g. Duragen), with some fibrinogen-coated (e.g. Tachosil or Tachocomb) to increase adhesion [41, 76, 88]. Other options included collagen sponges (e.g. Spongostan, Tissuefleece), gelatin sponges (Gelfoam), oxidised cellulose (Surgicel) [29, 79, 89], and nanofibrous scaffolds (e.g. ReDura) which may reduce graft infection through the promotion of native dura ingrowth [78]. Moreover, allografts, like synthetic materials, avoid the donor-related complications of autografts. For example, acellular dermis or dehydrated amniotic membrane, both of which are biocompatible and encourage native tissue growth [90–92]. Xenografts, such as equine pericardium sheet or equine collagen foil, retain many of these benefits, with some products having a lower infectious transmission profile (e.g. no bovine spongiform encephalopathy risk with equine products) [77, 93].

Finally, dural reconstruction with grafts may be augmented by employing the button technique [94]. This involved suturing a larger barrier graft (e.g. fascia lata) to a smaller barrier graft in a stacked fashion. This construct was manoeuvred so that the larger graft underlays the defect (intradural phase) whilst the small defect lies as an overlay (dural phase), producing a watertight plug. This technique may decrease CSFR rates in the context of high flow ioCSFL in EEA (decreasing CSFR rates from 45 to 10%, p = 0.03) in this subgroup [94].

Tissue glues and haemostatic agents can be used to consolidate the dural phase and are described across almost all phases of repair. In included studies, tissue glues were used to stabilise the repair construct, create a watertight sealant and/or fill dead space [4, 75, 95, 96]. The majority were fibrin-based glues (e.g. Evicel, Tisseel, autologous), but others included polyethylene glycol (e.g. Duraseal), hydrogel (Adherus) and cyanoacrylate (e.g. Bioglue, Cyanoacrylate) based agents. Fibrin-glues offer strong adhesion and are animal- or human-derived, and therefore may carry the risk of allergic response or infection [97, 98]. Some authors highlight the potential for autologous fibrin glue from serum (e.g. Vivostat), which is cost-effective and avoids the risk of immune reaction or infectious transmission [98, 99] but may not be feasible if the patient is unable to give this blood (e.g. anaemia). Autologous glues tend to be less viscous and have a slightly slower coagulation time than synthetic alternatives [99]. Polyethylene glycol and cyanoacrylate glues are entirely synthetic, solidify in seconds with strong adhesion [39, 100]. Taken together, equipoise remains about the optimal tissue glue [100, 101]. Moreover, haemostatic materials can be divided into those which are liquid at application and those solid at application. Liquid agents included Surgiflo and Floseal whilst solid agents included Surgicel and Avitene. Like tissue glues, they can be used in isolation or as part of multilayer graded regimes [30, 69, 102–104].

#### Barrier restoring: bony skull base

Rigid or semi-rigid materials used can be autografts, allografts, xenografts or synthetic (Table 2, Figs. 2, 3). Autologous options include cartilage (e.g. septal) and bone (e.g. vomer) grafts [105]. Some authors describe a skull base craniotomy (crafting and eventually replacing a bone flap from the sellar floor) instead of traditional craniectomy, repairing the bony integrity and providing a foundation for nasoseptal flap adhesion [105]. Allograft alternatives include cadaveric radiation-sterilized iliac bone [106]. Other approaches include the use of mouldable cement (hydroxyapatite or polymethylmethacrylate) [29, 70, 79, 89, 107, 108].

The bony phase may be augmented by the use of the gasket seal technique, in which a sheet-like graft (e.g. fascia lata) is placed as an oversized overlay to a bony skull base defect and a rigid graft (e.g. polyethylene, titanium, bone) is countersunk into the defect to create a watertight seal [109]. This technique was used in 20 papers, particularly in the context of large and high-flow defects, and is cited as an option that could potentially spare the need for vascularised repair, lumbar drainage and nasal packing [109–111]. However, it requires a suitable bony rim and may not be possible in multiplanar defects [110, 112]. A modified version, the “one-piece gasket-seal” used a unitized (via sutures) Medpor and fascia construct, reducing the technical demand of placement, decreasing the time required of manoeuvring the two components into position intra-nasally, and avoiding the need for an underlying fat graft to provide counterpressure during countersinking of the rigid buttress [57].

#### Barrier restoring: nasal

Pedicled vascular flaps (Supplementary information 6) have been a critical advancement in skull base repair methods—used in 169 of included papers (Figs. 2, 3). They can be harvested from nasal (e.g. nasoseptal flaps) or extra-nasal (e.g. pericranial) regions. Their use is particularly described in high flow ioCSFL with large skull base defects, as part of graded multilayer repair protocols [34, 37, 52, 75, 113–119].

The sentinel pedicled vascular flap technique, the nasoseptal flap (NSF), was described by Hadad-Bassagasteguy in 2006. It boasts a rich vascular pedicle (based on the posterior nasoseptal arteries), making it robust and versatile. The NSF was the first-line for vascularised repairs of anterior, middle, clival, sellar and lateral/parasellar defects across many protocols [31, 37, 75, 113, 120]. It is technically easy to raise, although there is a learning curve associated with its effective use [75, 121]. Principle disadvantages of NSF included sinonasal morbidity (e.g. crusting, loss of smell, sinusitis, nasal perforations, adhesions, synechiae) and potential iatrogenic damage to the raised flap/pedicle intraoperatively [52, 122–124]. Rivera-Serrano et al. described the “rescue” NSF technique, which allows protection of the vascular pedicle early in the operation so that the choice to raise a full NSF or not is available at the reconstruction phase of the operation [125]. Several modifications of this “rescue” technique are described, including the “pedicle sparing-transposition technique” [126], “Sigmoid incision rescue flap” [127], and the “hemi-transeptal” technique [122, 124]. For larger EEA defects, bilateral NSFs have been described (with some authors also advocating for their use in the context of expected radiotherapy) [116, 117, 121, 126], as well as extended unilateral NSFs which incorporate inferior turbinate mucosa and lateral nasal wall mucosa [71]. Finally, NSFs were often used as part of multilayer repair regime, one example of this is the “3F” (“fat, flap, flash”) technique used after EEA in which a fat graft fills the dead space (“fat”), stabilised with fibrin glue, then covered by NSF (“flap”) which is supported by Merocel packing—allowing fast mobilisation (“flash”) [50].

In the absence of the NSF, multiple other nasal and extra-nasal flaps were described (Supplementary information 6), with their use tailored to defect sizes and location. For anterior defects: middle turbinate flaps, pericranial flaps, buccinator flap, palatal, occipital, radial (free) flaps or vastus lateralis (free) flaps were used [31, 120, 121, 128–133]. For posterior/clival defects, options included the temporoparietal flap and inferior turbinate flaps [31, 120, 132, 134]. Other options included the lateral nasal wall, sellar floor, superior turbinate, “U” inverted rhinopharyngeal and nasopharyngeal (mucosal and muscle) flaps [28, 59, 135, 136].

Whilst vascularised flaps form part of the skull base reconstruction, measures such as buttresses and packing provide external stabilisation (temporary or permanent) to the established construct. Buttressing materials were described in 94 studies and used particularly in the context of ioCSFL. They were autologous (e.g. bone) or synthetic (e.g. Medpor, titanium mesh) [92, 109, 131, 137–140]. Medpor buttresses were commonly used, having the advantage of being mouldable, porous and relatively inert [57, 92, 137, 139]. Titanium mesh, although strong and mouldable, is not porous. Therefore, it prevents tissue ingrowth (which improves stabilisation and decreases infection) [139]. Additionally, nasal packing was described in 96 studies, being balloon or non-balloon based. Balloon-based methods included Foley catheters, Merocel sponges and Rapid Rhino balloons [37, 44, 46, 57, 115]. The shape of the balloons may be important for support distribution, stability and patient comfort—with tubular-shaped balloons (e.g. Merocel) being considered more favourable by some authors than spherical (e.g. Foley) counterparts [44]. Non-balloon-based packing (e.g. Nasopore, iodoform gauze, polyvinyl alcohol sponge, bismuth soaked ribbon gauze, Gelfoam packing), maybe more comfortable, more mouldable, and thus provided a more even support distribution across the repair [115, 141]. Defect location may inform the choice of packing, for example, Foley catheters for sellar or parasellar support and tampon sponges for cribriform or clival support [37]. Antibiotic impregnation of packing (e.g. gentamicin-soaked Gelfoam, Merocel covered in bacitracin ointment) may reduce colonisation of bacteria on the repair construct and may improve underlying healing [44, 71, 142].

#### Pressure reducing: CSF diversion

CSF diversion can be used for treatment or prevention of CSFR (Figs. 2, 3, Table 2). Temporary diversion measures include lumbar drainage (often placed pre-operatively for high-risk CSFR cases or immediately postoperatively in reaction to ioCSFL), lumbar puncture, or rarely, external ventricular drainage (e.g. if concomitant acute hydrocephalus). Medium- and long-term options include ventriculoperitoneal and lumbar shunts—again, usually in the context of concomitant underlying hydrocephalus. A recent randomised controlled trial by Zwagerman et al. suggested that perioperative lumbar drain (in the context EEA with dural defects > 1 cm^2^ and high flow ioCSFL) decreased CSFR rates when combined with nasoseptal flap repair [34]. In this study, lumbar drains were inserted immediately postoperatively (under the same general anaesthetic), draining 10 ml/h for 3 days. 8.2% of those with lumbar drainage and 21.2% of those without lumbar drainage had CSFR during follow-up (p = 0.03) [34]. Observational studies echo the utility of perioperative (post-procedural) in this high CSFR risk context, in combination with NSF [10, 115, 143]. Additionally, pre-procedural lumbar drainage may facilitate tumour resection (by allowing drainage of CSF or injection of intrathecal saline [49]) and allow the use of other intrathecal adjuncts (e.g. fluorescein) [144, 145]. The evidence in low flow ioCSFL and smaller defects (TSA) is less robust [146] and again, largely observational [34, 147, 148].

### Postoperative CSF rhinorrhoea

The methods of confirming and grading intraoperative CSF leak rates are summarised in Table 3. The pooled incidence of ioCSFL rate (if reported) was 4.6% (CI 3.7–5.6%, I^2^ 98.1%, Cochran’s Q p < 0.01) for TSA, 9.6% (CI 9.1–9.8%, I^2^ 89.2%, Cochran’s Q: p < 0.01) for EEA and 7.9% (CI 6.4–8.9%, I^2^ 89.2%, Cochran’s Q: p < 0.01) for studies describing both approaches. Additionally, the methods of confirming, grading, and managing postoperative CSFR are summarised in Table 3. The pooled incidence of CSFR rates (if reported) was 3.7% (CI 3–4.5%, I^2^ 72.8%, Cochran’s Q: p < 0.01) for TSA, 9% (CI 7.2–11.3%, I^2^ 54.9%, Cochran’s Q: p < 0.01) for EEA and 5.3% (CI 3.4–7%, I^2^ 90.2%, Cochran’s Q: p < 0.01) for studies describing both. Totally heterogeneous protocols used across the included studies did not allow for meaningful meta-analyses of techniques and materials.

## Discussion

### Principle findings

This systematic review of skull base repair during endonasal pituitary and skull base tumour resection has highlighted the wide scope of repair techniques used, which is increasing yearly in parallel with the increasing indications for endonasal approaches.

Reconstruction of the skull base is challenging—using long rigid instruments in a restricted working space to place repair materials (with varying risk and morbidity profiles) against the forces of gravity and overlying dependent intracranial structures [12]. In the context of these challenges, this article presents a comprehensive taxonomy that highlights the principles of barrier restoration, pressure relief and the use of staged approaches to prevent CSFR (Figs. 2, 3). Protocols were totally heterogeneous between author groups—ranging from the use of one material at a single level (intradural, dural, bony, nasal) to a complex multilevel closure. Most protocols employed a graded protocol, tailoring the extent of reconstruction to the risk of postoperative CSF leak. Techniques most commonly used in the absence of ioCSFL or the presence of low-grade ioCSFL included fat grafts (most commonly abdominal), sheet-like grafts (e.g. fascia lata) or synthetic materials (e.g. Surgicel) and non-balloon nasal packs (e.g. Nasopore). On the other hand, in the context of high-grade ioCSFL, studies described multilayer regimes with vascularized flaps, gasket sealing, balloon-based packing and lumbar drain use to prevent CSFR.

To date, there is sparse high-level evidence to recommend most repair methods, except for lumbar drain use in the context of large dural defect size and high flow intraoperative CSF leak (Oxford CEBM: Grade B recommendation) [34]. Pooled CSFR incidence was 3.7% (CI 3–4.5%) for TSA, 9% (CI 7.2–11.3%) for EEA, and 5.3% (CI 3.4–7%) for studies describing both. Totally heterogeneous protocols used across the included studies did not allow for meaningful meta-analyses of techniques and materials.

### Findings in the context of other syntheses

Several reviews (qualitative and quantitative) exploring the available repair techniques in iatrogenic skull base neurosurgery exist. In terms of narrative reviews, Hannan et al. explored methods of skull base repair with historical reference and technical details [13]. Reye et al. focused on pedicled and free flaps through narrative discussion, exploring the indications and complication profile for vascularised repair options available [20]. Sughrue and Aghi provided an insight into the challenges posed by pathology in different anatomical regions [19]. Oakley et al. reviewed the literature to produce limited recommendations for various repair categories as well as CSFR management factors not investigated in our review (including the use of perioperative prophylactic antibiotics, postoperative bed rest, CPAP; and the timing of air travel postoperatively) [22].

Where meta-analyses have been attempted to compare repair techniques [11, 17, 18], strict inclusion criteria have been used to enable specific comparisons. Harvey et al. reviewed 38 studies where the size of the bony/dural defect was reported in order to compare vascularised and non-vascularised autografts in the context of large skull base defects [11]. Postoperative CSFR rates were less in studies using vascular flaps as part of their regime (15.6% vs. 6.7% with avascular repair) [11]. Soudry et al. explored the effectiveness of multilayer graded regimes in the context of ioCSFL [17]. In the 22 studies included, a meta-analysis suggested that vascularised flaps provided maximal benefit in high flow leak situations reducing CSFR rates from 18 to 6% with their use (whilst non-vascularised multilayer regimes sufficed for low ioCSFL) [17]. Iavarone et al. commented specifically on the treatment of CSFR (including non-iatrogenic aetiologies), highlighting the introduction of multilayer and vascularised repair in EEA, which may reduce CSFR rates < 5% [18]. However, these studies acknowledge the limitations of their meta-analyses in light of the repair technique and CSFR reporting heterogeneity in the primary literature [11, 17, 18].

### Strengths and limitations

The principal strength of this review is the systematic and sensitive search strategy, capturing the breadth of surgical practice in this field. By focussing on articles orientated towards repair techniques or CSFR risk factors, a detailed exploration of repair technique roles, categories modifications and disadvantages was synthesised. The taxonomy generated aims to be comprehensive but, owing to the multifunctionality of certain repair materials (e.g. Surgicel), has overlapping techniques within levels. General limitations include the pragmatic exclusion of case series which captured surgeries but with a focus other than skull base repair or CSFR. Of the included studies, there was inconsistent reporting of key data elements (patient demographics, follow-up time, tumour type, tumour size, dural or bony defect, ioCSFL severity), heterogeneity in definitions of CSFR and mixed case populations from which individual data cannot be extracted. This prevented accurate meta-analysis on the comparative CSFR incidence for various repair techniques. We have previously described meta-analyses of CSFR in more defined populations, with analysis on selected repair technique effectiveness [8, 149], however this paper aimed to capture the breadth of repair techniques and material currently in use. A useful future target would be the development of a standardised core data and/or outcome set to allow comprehensive quantitative synthesis of the literature.

## Conclusions

Modern skull base reconstructive protocols are heterogeneous. Most protocols are multi-layered and graded to case-specific risk factors for postoperative CSF rhinorrhoea. This review captures the scope of current repair methods, categorised according to function and anatomical level. Currently, there is limited evidence to guide the optimal repair technique after pituitary and skull base tumour resection. Prospective multicentre studies and registries will be useful in determining current practice and outcomes with a view to generating a consensus, and possibly providing the basis for further randomised studies [150].

## Supplementary Information

Below is the link to the electronic supplementary material.

Supplementary file1 (DOCX 15 kb) Supplementary information 1: Search strategy.

Supplementary file2 (DOCX 50 kb) Supplementary information 2: Bespoke risk of bias scoring system and score per study.

Supplementary file3 (TIFF 2197 kb) Supplementary information 3: Number of studies published over time.

Supplementary file4 (DOCX 136 kb) Supplementary information 4: Summary of repair protocols described per study.

Supplementary file5 (DOCX 21 kb) Supplementary information 5: Taxonomy of repair techniques with the number of studies reporting the use of the repair technique.

Supplementary file6 (TIFF 97663 kb) Supplementary information 6: Nasal and extra-nasal vascularised flaps used in skull base repair.

## Data Availability

Available upon reasonable request.

## Abbreviations

CSF: Cerebrospinal fluid
ioCSFL: Intraoperative cerebrospinal fluid leak
CSFR: Cerebrospinal fluid rhinorrhoea
TSA: Transsphenoidal approach
EEA: Endonasal endoscopic approach
