# Supplementary material for: Skull base repair following endonasal pituitary and skull base tumour resection: a systematic review
Source: Pituitary. 2021 May 10;24(5):698–713. doi: 10.1007/s11102-021-01145-4 (PMC8416859; doi:10.1007/s11102-021-01145-4)
Supplement: Supplementary file 1 — Supplementary file1 (DOCX 15 kb) Supplementary information 1: Search strategy. [file 11102_2021_1145_MOESM1_ESM.docx]

Supplementary Information 1: Search strategy

| **Study question** | |
| --- | --- |
| Population | Patients undergoing trans-sphenoidal surgery for pituitary adenoma AND patients undergoing an *expanded* endoscopic endonasal approach for anterior skull base pathology |
| Intervention | Method(s) of intraoperative skull base repair used |
| Comparator | No repair/various methods used |
| Outcome | CSF Rhinorrhea |
|  |  |
| **Decision criteria** | |
| Inclusion Criteria | Papers discussing trans-sphenoidal surgery for pituitary adenoma AND/OR expanded endoscopic endonasal approach for anterior skull base pathology |
|  | Reporting methods of specific skull base repair methods used |
|  | Reporting outcomes of CSF Rhinorrhoea |
|  | Within the last 20 years |
|  |  |
| Exclusion Criteria | Case-reports |
|  | Case series <3 patients |
|  | Editorials |
|  | Reviews |
|  | Abstracts |
|  | Animal studies |
|  | Cadaveric study |
|  | No specific outcome reported |
|  | Full text not available in English |
|  |  |
| **Database search** | |
| Search Terms | (Transsphenoidal OR endonasal OR EEA OR EEEA) AND (pituitary OR sellar OR suprasellar OR adenoma OR craniopharyngioma OR Rathke OR meningioma OR chordoma OR chondrosarcoma OR “skull base”) AND (“Cerebrospinal fluid” OR CSF) |
| Limits | Last 20 years, English |
