# Supplementary material for: Skull base repair following endonasal pituitary and skull base tumour resection: a systematic review
Source: Pituitary. 2021 May 10;24(5):698–713. doi: 10.1007/s11102-021-01145-4 (PMC8416859; doi:10.1007/s11102-021-01145-4)
Supplement: Supplementary file 2 — Supplementary file2 (DOCX 50 kb) Supplementary information 2: Bespoke risk of bias scoring system and score per study. [file 11102_2021_1145_MOESM2_ESM.docx]

Supplementary Information 2: Risk of Bias and Quality Assessment

***Bias Domains & Scoring:***

1. **Description of patient population: degree of granularity with describing patient characteristics.**
   1. High risk of bias (scored 1): No description of included pathology (i.e. anterior skull base pathology).
   2. Low risk of bias (scored 0): Clear description of included pathology types (i.e. pituitary adenoma, chordoma, etc.).
2. **Description of surgical route and technique: identifiability of treatment groups.**
   1. High risk of bias (scored 1): No description of which percentage of cases by surgical approach (EEA, TSA endoscopic, TSA microscopic) and first/revision surgery.
   2. Low risk of bias (scored 0): Clear description of percentage of cases by surgical approach (EEA, TSA endoscopic, TSA microscopic) and first/revision surgery.
3. **Description of repair technique: clarity of description and identifiability of each treatment group.**
   1. High risk of bias (scored 1): No description in which percentage of cases the repair technique was used.
   2. Low risk of bias (scored 0): Description in which percentage/proportion of cases a repair technique was used (e.g. repair A was used for TSA only or repair B was used for Esposito Grade 3 cases).
4. **Description of intraoperative CSF leak: clarity of description and grading of intraoperative CSF leak.**
   1. High risk of bias (scored 1): No definition or classification provided.
   2. Low risk of bias (scored 0): Severity/classification described (high flow vs low flow or Esposito-Kelly classification).
5. **Separate reporting of intraoperative and postoperative CSF leak.**
   1. High risk of bias (scored 1): No distinguishment of results between intraoperative and postoperative CSF leak.
   2. Low risk of bias (scored 0): Results separately on both intraoperative and postoperative CSF leak.

***Scoring per paper:***

| **Author** | **Year** | **PMID** | **A** | **B** | **C** | **D** | **E** | **Total** |
| --- | --- | --- | --- | --- | --- | --- | --- | --- |
| Tewfik, MA et al. | 2014 | 4240765 | 0 | 1 | 0 | 1 | 0 | 2 |
| Citardi, M J et al. | 2000 | 10711336 | 0 | 1 | 1 | 1 | 0 | 3 |
| Seiler, R W and Mariani, L | 2000 | 11059655 | 1 | 0 | 0 | 1 | 0 | 2 |
| Kelly, D F et al. | 2001 | 11564250 | 1 | 0 | 0 | 0 | 0 | 1 |
| Kim, J et al. | 2002 | 12087514 | 0 | 0 | 1 | 1 | 1 | 3 |
| Cappabianca, P et al. | 2002 | 12445341 | 0 | 0 | 1 | 1 | 0 | 2 |
| Kumar, A et al. | 2003 | 12464532 | 0 | 0 | 1 | 1 | 1 | 3 |
| Sonnenburg, R et al. | 2003 | 14750609 | 0 | 0 | 1 | 1 | 0 | 2 |
| Kitano, M et al. | 2004 | 15028140 | 0 | 0 | 0 | 1 | 1 | 2 |
| Cappabianca, P et al. | 2004 | 15336865 | 0 | 1 | 0 | 1 | 0 | 2 |
| van Aken, M O et al. | 2004 | 15761657 | 0 | 1 | 0 | 1 | 0 | 2 |
| Nishioka, J. et al. | 2005 | 16047106 | 0 | 0 | 0 | 1 | 0 | 1 |
| Sade, B et al. | 2006 | 16328773 | 0 | 1 | 0 | 1 | 0 | 2 |
| Cappabianca, P et al. | 2006 | 16427407 | 0 | 0 | 1 | 0 | 0 | 1 |
| Couldwell, W et al. | 2006 | 16599417 | 1 | 1 | 1 | 1 | 1 | 5 |
| Silva, L R F et al. | 2006 | 16708337 | 1 | 0 | 0 | 1 | 0 | 2 |
| Seda, L et al. | 2006 | 16793438 | 1 | 1 | 0 | 1 | 0 | 3 |
| Hadad, G et al. | 2006 | 17003708 | 0 | 1 | 1 | 1 | 0 | 3 |
| Dusick, J et al. | 2006 | 17015111 | 0 | 0 | 0 | 0 | 0 | 0 |
| Yano, S et al. | 2007 | 17210302 | 0 | 0 | 0 | 0 | 0 | 0 |
| Esposito, F et al. | 2007 | 17415166 | 0 | 0 | 0 | 0 | 0 | 0 |
| Kitano, M et al. | 2007 | 17876222 | 0 | 1 | 0 | 1 | 0 | 2 |
| Cavallo, L et al. | 2007 | 17937213 | 0 | 1 | 1 | 1 | 1 | 4 |
| Sherman, J et al. | 2008 | 18054621 | 0 | 1 | 0 | 1 | 0 | 2 |
| El-Banhawy, O et al. | 2008 | 18416976 | 0 | 1 | 0 | 1 | 1 | 3 |
| Tamasauskas, A et al. | 2008 | 18469507 | 0 | 0 | 0 | 1 | 0 | 1 |
| Yoon, T et al. | 2008 | 18568522 | 0 | 1 | 0 | 1 | 0 | 2 |
| Leng, SB et al. | 2008 | 18596534 | 0 | 1 | 0 | 1 | 0 | 2 |
| Kassam, AB et al. | 2008 | 18728603 | 1 | 0 | 0 | 1 | 0 | 2 |
| El-Banhawy, OA et al. | 2008 | 19240829 | 0 | 0 | 1 | 1 | 1 | 3 |
| El-Sayed, IH et al. | 2008 | 19412408 | 0 | 1 | 1 | 1 | 0 | 3 |
| Rabadan, A et al. | 2009 | 19430889 | 1 | 1 | 0 | 1 | 0 | 3 |
| Nishioka, H et al. | 2009 | 19499173 | 1 | 0 | 0 | 1 | 0 | 2 |
| Locatelli, D et al. | 2009 | 19551337 | 1 | 1 | 1 | 1 | 1 | 5 |
| Zanation, A et al. | 2009 | 19807986 | 0 | 0 | 0 | 0 | 0 | 0 |
| Ahn, JY et al. | 2009 | 19935004 | 0 | 0 | 1 | 1 | 1 | 3 |
| Cappabianca, P et al. | 2010 | 20049488 | 0 | 0 | 0 | 0 | 0 | 0 |
| Nyquist, G et al. | 2010 | 20172375 | 0 | 0 | 1 | 1 | 1 | 3 |
| Patel, MR et al. | 2010 | 20173545 | 0 | 1 | 1 | 1 | 1 | 4 |
| Horiguchi, K et al. | 2010 | 20195676 | 0 | 1 | 0 | 1 | 0 | 2 |
| Moliterno, JA et al. | 2010 | 20345225 | 0 | 0 | 0 | 1 | 0 | 1 |
| Luginbuhl, PG et al. | 2010 | 20422679 | 0 | 1 | 0 | 0 | 0 | 1 |
| Sciarretta, V et al. | 2010 | 20533135 | 0 | 1 | 0 | 0 | 0 | 1 |
| Romero, AD et al. | 2010 | 20602047 | 0 | 0 | 1 | 1 | 0 | 2 |
| Acerbi, F et al. | 2010 | 21099571 | 0 | 0 | 1 | 1 | 0 | 2 |
| Cohen-Gadol, A et al. | 2010 | 21132615 | 1 | 1 | 0 | 1 | 0 | 3 |
| Cho, JM et al. | 2011 | 21206312 | 0 | 1 | 0 | 0 | 0 | 1 |
| Kaptain, GJ et al. | 2011 | 21206313 | 0 | 0 | 0 | 1 | 0 | 1 |
| Kong, D et al. | 2011 | 21240667 | 1 | 0 | 0 | 0 | 0 | 1 |
| Patel, MR et al. | 2010 | 21772795 | 1 | 1 | 0 | 0 | 0 | 2 |
| Burkett, C et al. | 2011 | 21868232 | 0 | 0 | 0 | 0 | 0 | 0 |
| Bergsneider, M et al. | 2012 | 22095220 | 1 | 0 | 0 | 0 | 0 | 1 |
| Garcia-Navarro, V et al. | 2013 | 22120292 | 1 | 1 | 1 | 0 | 0 | 3 |
| Mortuaire, G et al. | 2012 | 22245756 | 0 | 1 | 0 | 1 | 0 | 2 |
| Kim, S et al. | 2011 | 22323936 | 0 | 1 | 1 | 0 | 1 | 3 |
| Eloy JA et al. | 2012 | 22344288 | 0 | 0 | 1 | 0 | 0 | 1 |
| Chung, S et al. | 2012 | 22350441 | 0 | 1 | 0 | 0 | 0 | 1 |
| Kobayashi, H et al. | 2011 | 22451799 | 0 | 0 | 1 | 1 | 1 | 3 |
| Mehta, G et al. | 2012 | 22482793 | 0 | 0 | 0 | 1 | 0 | 1 |
| Eloy JA, et al. | 2012 | 22522971 | 0 | 0 | 0 | 0 | 0 | 0 |
| Eloy JA, et al. | 2012 | 22528686 | 0 | 1 | 0 | 0 | 0 | 1 |
| Adappa N, et al. | 2012 | 22566127 | 0 | 1 | 0 | 1 | 1 | 3 |
| Liu, JK et al. | 2012 | 22655696 | 1 | 1 | 1 | 0 | 0 | 3 |
| Peizhi, Z et al. | 2012 | 22665002 | 0 | 1 | 0 | 1 | 0 | 2 |
| Lee, DH et al. | 2012 | 22801166 | 0 | 0 | 1 | 1 | 0 | 2 |
| Malik, MU et al. | 2012 | 22899227 | 0 | 0 | 0 | 1 | 0 | 1 |
| McCoul, E et al. | 2014 | 23022644 | 0 | 0 | 0 | 1 | 1 | 2 |
| Eloy, JA et al. | 2013 | 23038626 | 0 | 1 | 0 | 0 | 0 | 1 |
| Eloy, JA et al. | 2012 | 23070802 | 0 | 0 | 0 | 1 | 1 | 2 |
| Learned, KO et al. | 2013 | 23219190 | 1 | 1 | 0 | 1 | 1 | 4 |
| Berker, M et al. | 2013 | 23265578 | 1 | 0 | 1 | 0 | 0 | 2 |
| Iannelli, A et al. | 2014 | 23307310 | 0 | 1 | 1 | 1 | 1 | 4 |
| Cavallo, LM et al. | 2014 | 23313259 | 0 | 0 | 0 | 1 | 1 | 2 |
| Munich, SA et al. | 2013 | 23319331 | 0 | 0 | 0 | 1 | 0 | 1 |
| Eloy, JA et al. | 2013 | 23333162 | 1 | 0 | 1 | 0 | 0 | 2 |
| Campos, F et al. | 2013 | 23358164 | 0 | 1 | 1 | 1 | 0 | 3 |
| Mascarenhas, L et al. | 2014 | 23403355 | 1 | 0 | 1 | 0 | 0 | 2 |
| Eloy, JA et al. | 2013 | 23483459 | 0 | 0 | 0 | 0 | 0 | 0 |
| Kim, BY et al. | 2013 | 23553463 | 0 | 0 | 1 | 1 | 0 | 2 |
| Otto, B et al. | 2013 | 23712564 | 0 | 1 | 1 | 0 | 0 | 2 |
| Patel, K et al. | 2013 | 23724985 | 1 | 1 | 0 | 0 | 0 | 2 |
| Husain, Q et al. | 2013 | 23772323 | 1 | 1 | 1 | 0 | 0 | 3 |
| Jakimovski, D et al. | 2014 | 23811068 | 0 | 0 | 0 | 1 | 0 | 1 |
| Patel, MR et al. | 2014 | 23877996 | 0 | 1 | 1 | 1 | 0 | 3 |
| Iacoangeli, M et al. | 2014 | 24011138 | 0 | 0 | 0 | 1 | 1 | 2 |
| Shin, J et al. | 2013 | 24036728 | 0 | 0 | 1 | 1 | 0 | 2 |
| Xuejian, W et al. | 2013 | 24101261 | 0 | 0 | 1 | 0 | 0 | 1 |
| Cavallo, LM et al. | 2013 | 24251248 | 0 | 0 | 0 | 0 | 0 | 0 |
| Yildirim, AE et al. | 2013 | 24310456 | 0 | 1 | 0 | 1 | 1 | 3 |
| Banu, MA et al. | 2014 | 24315752 | 0 | 0 | 0 | 1 | 0 | 1 |
| Gaynor, BG et al. | 2013 | 24436906 | 0 | 1 | 0 | 1 | 0 | 2 |
| Wessell, A et al. | 2013 | 24436929 | 0 | 1 | 1 | 0 | 0 | 2 |
| Brunworth, J et al. | 2013 | 24498521 | 0 | 0 | 1 | 0 | 0 | 1 |
| Gruss, CL et al. | 2014 | 24898075 | 1 | 1 | 0 | 1 | 1 | 4 |
| Hu, F et al. | 2015 | 24915070 | 0 | 1 | 0 | 0 | 0 | 1 |
| Jalessi, M et al. | 2013 | 24926179 | 0 | 0 | 0 | 0 | 0 | 0 |
| Thorp, BD et al. | 2014 | 25270144 | 0 | 0 | 0 | 1 | 0 | 1 |
| Ivan, ME et al. | 2015 | 25439754 | 1 | 0 | 1 | 1 | 0 | 3 |
| Ishii, Y et al. | 2014 | 25446379 | 0 | 1 | 1 | 1 | 1 | 4 |
| Kim, EH et al. | 2015 | 25584954 | 0 | 0 | 0 | 1 | 0 | 1 |
| Hong, CK et al. | 2015 | 25630424 | 0 | 1 | 0 | 1 | 0 | 2 |
| Ishii, Y et al. | 2015 | 25675847 | 0 | 1 | 0 | 1 | 1 | 3 |
| Hayashi, N et al. | 2015 | 25685643 | 0 | 0 | 0 | 1 | 0 | 1 |
| Trinh, VT et al. | 2015 | 25685645 | 0 | 0 | 0 | 1 | 1 | 2 |
| Liebelt, BD et al. | 2015 | 25731796 | 0 | 1 | 0 | 1 | 1 | 3 |
| Zhan, R et al. | 2015 | 26080170 | 0 | 1 | 0 | 0 | 0 | 1 |
| Freyschlag, CF et al. | 2016 | 26091112 | 0 | 1 | 0 | 1 | 0 | 2 |
| Sanders-Taylor, CA et al. | 2015 | 26225317 | 0 | 1 | 0 | 1 | 0 | 2 |
| Fiorindi, A et al. | 2015 | 26225321 | 0 | 0 | 0 | 1 | 0 | 1 |
| Park, J et al. | 2015 | 26279811 | 1 | 0 | 0 | 0 | 1 | 2 |
| Amano, K et al. | 2016 | 26338198 | 1 | 1 | 0 | 1 | 1 | 4 |
| Hara, T et al. | 2015 | 26341445 | 1 | 0 | 0 | 0 | 0 | 1 |
| Horiguchi, K et al. | 2016 | 26886779 | 1 | 0 | 0 | 0 | 1 | 2 |
| Ozawa, H et al. | 2016 | 26901123 | 0 | 0 | 0 | 1 | 0 | 1 |
| Magro, E et al. | 2016 | 26902781 | 0 | 0 | 0 | 1 | 0 | 1 |
| Zenga, F et al. | 2016 | 26937335 | 0 | 1 | 0 | 1 | 1 | 3 |
| Wang, X et al. | 2016 | 26956812 | 0 | 1 | 1 | 1 | 1 | 4 |
| Moon, JH et al. | 2016 | 26967785 | 0 | 1 | 0 | 1 | 0 | 2 |
| Nix, P et al. | 2016 | 27008345 | 1 | 0 | 0 | 1 | 0 | 2 |
| Dehdashti, AR et al. | 2016 | 27175324 | 0 | 0 | 0 | 1 | 1 | 2 |
| Park, W et al. | 2016 | 27302560 | 0 | 1 | 1 | 1 | 1 | 4 |
| Amin, S et al. | 2016 | 27323957 | 0 | 0 | 1 | 0 | 0 | 1 |
| Ismail, M et al. | 2016 | 27408609 | 0 | 1 | 1 | 1 | 0 | 3 |
| Thomas, R et al. | 2016 | 27441162 | 0 | 1 | 0 | 0 | 0 | 1 |
| Fathalla, H et al. | 2017 | 27558362 | 1 | 1 | 0 | 1 | 0 | 3 |
| Fishpool, S et al. | 2017 | 27586390 | 0 | 0 | 0 | 1 | 0 | 1 |
| Roxbury, CR et al. | 2016 | 27657903 | 0 | 1 | 0 | 0 | 0 | 1 |
| Gandham, E et al. | 2017 | 28084257 | 0 | 0 | 1 | 1 | 1 | 3 |
| Soldatova, L et al. | 2017 | 28180044 | 1 | 1 | 1 | 1 | 1 | 5 |
| Zhou, Q et al. | 2017 | 28192266 | 0 | 0 | 0 | 0 | 0 | 0 |
| Yoo, F et al. | 2017 | 28321377 | 0 | 1 | 0 | 1 | 1 | 3 |
| Fnais, N et al. | 2017 | 28321378 | 1 | 1 | 0 | 1 | 0 | 3 |
| Sotomayor-Gonzalez, A et al. | 2017 | 28321383 | 0 | 1 | 1 | 1 | 0 | 3 |
| Thawani, JP et al. | 2017 | 28321384 | 0 | 0 | 0 | 1 | 0 | 1 |
| Fraser, S et al. | 2018 | 28598276 | 0 | 1 | 1 | 0 | 0 | 2 |
| Pereira, EA et al. | 2017 | 28676314 | 1 | 1 | 0 | 1 | 0 | 3 |
| So, J et al. | 2017 | 28684150 | 0 | 1 | 0 | 0 | 0 | 1 |
| Jyotirmay, H et al. | 2017 | 28764208 | 1 | 1 | 1 | 1 | 1 | 5 |
| Jeon, C et al. | 2017 | 28765065 | 1 | 1 | 1 | 0 | 0 | 3 |
| Zhang, C et al. | 2017 | 28872159 | 0 | 0 | 1 | 1 | 0 | 2 |
| Kerr, EE et al. | 2017 | 28875119 | 0 | 1 | 1 | 1 | 1 | 4 |
| Strickland, BA et al. | 2018 | 28960156 | 1 | 0 | 0 | 1 | 0 | 2 |
| Nakayama, N et al. | 2018 | 29107157 | 0 | 1 | 0 | 1 | 0 | 2 |
| Li, Z et al. | 2018 | 29194274 | 0 | 0 | 0 | 0 | 0 | 0 |
| Cohen, S et al. | 2018 | 29253284 | 0 | 0 | 0 | 1 | 1 | 2 |
| Jonathan, GE et al. | 2018 | 29322972 | 0 | 0 | 0 | 1 | 0 | 1 |
| Ye, Y et al. | 2017 | 29384918 | 0 | 1 | 1 | 1 | 0 | 3 |
| Schuss, P et al. | 2018 | 29388119 | 0 | 0 | 0 | 0 | 0 | 0 |
| Patel, PN et al. | 2018 | 29405885 | 0 | 0 | 1 | 1 | 0 | 2 |
| Liu, JK et al. | 2018 | 29421455 | 1 | 0 | 0 | 1 | 1 | 3 |
| Cong, Z et al. | 2018 | 29436283 | 0 | 1 | 1 | 1 | 1 | 4 |
| Barger, J et al. | 2018 | 29527390 | 0 | 0 | 0 | 1 | 0 | 1 |
| Geltzeiler, M et al. | 2018 | 29554360 | 0 | 0 | 1 | 1 | 1 | 3 |
| Conger, A et al. | 2018 | 29749920 | 1 | 0 | 0 | 0 | 0 | 1 |
| Kuan, EC et al. | 2018 | 29868324 | 0 | 0 | 0 | 0 | 0 | 0 |
| Theys, T et al. | 2018 | 29872915 | 0 | 0 | 0 | 1 | 0 | 1 |
| Caggiano, C et al. | 2018 | 29935316 | 1 | 1 | 0 | 1 | 0 | 3 |
| Rasmussen, J et al. | 2018 | 29969737 | 0 | 0 | 0 | 1 | 0 | 1 |
| Ogiwara, T et al. | 2018 | 29982857 | 0 | 1 | 0 | 0 | 0 | 1 |
| Roca, E et al. | 2019 | 30124966 | 0 | 0 | 0 | 1 | 0 | 1 |
| Scagnelli, RJ et al. | 2019 | 30368014 | 0 | 0 | 0 | 1 | 0 | 1 |
| Ishikawa, T et al. | 2018 | 30485207 | 0 | 1 | 0 | 0 | 1 | 2 |
| Zwagerman, N et al. | 2018 | 30485224 | 1 | 1 | **1** | 0 | 0 | 3 |
| Zhang, C et al. | 2018 | 30535967 | 0 | 1 | 0 | 0 | 0 | 1 |
| Farrell, NF et al. | 2019 | 30579017 | 1 | 0 | 1 | 1 | 1 | 4 |
| Rieley, W et al. | 2019 | 30628938 | 0 | 0 | 1 | 1 | 1 | 3 |
| Kim, EH et al. | 2019 | 30649447 | 0 | 0 | 0 | 0 | 0 | 0 |
| Moon, JH et al. | 2019 | 30738381 | 0 | 0 | 1 | 0 | 0 | 1 |
| Fujita, Y et al. | 2019 | 30814422 | 0 | 0 | 0 | 0 | 0 | 0 |
| Cavallo, L et al. | 2019 | 30904811 | 0 | 0 | 0 | 0 | 0 | 0 |
| Umamaheswaran, P et al. | 2019 | 30906716 | 0 | 1 | 0 | 0 | 0 | 1 |
| Ruggeri, AG et al. | 2019 | 30908447 | 0 | 0 | 1 | 1 |  | 2 |
| Kessler, RA et al. | 2019 | 31062526 | 0 | 0 | 1 | 1 | 0 | 2 |
| Singh, C et al. | 2019 | 31070119 | 0 | 1 | 1 | 1 | 0 | 3 |
| Wardas, P et al. | 2019 | 31119003 | 1 | 0 | 0 | 1 | 0 | 2 |
| Slavnic, D et al. | 2019 | 31143295 | 0 | 0 | 0 | 0 | 0 | 0 |
| Gode, S et al. | 2019 | 31143571 | 0 | 1 | 1 | 0 | 0 | 2 |
| Eichberg, DG et al. | 2019 | 31372758 | 0 | 0 | 0 | 1 | 0 | 1 |
| Carnevale, C et al. | 2019 | 31495344 | 0 | 1 | 0 | 1 | 0 | 2 |
| Kutlay, M et al. | 2020 | 31550544 | 0 | 1 | 0 | 0 | 0 | 1 |
| Jandali, D et al. | 2020 | 31784142 | 0 | 1 | 1 | 1 | 0 | 3 |
| Xue, H et al. | 2019 | 31832806 | 0 | 0 | 1 | 0 | 0 | 1 |
| Liu, B et al. | 2020 | 31881340 | 0 | 1 | 0 | 1 | 0 | 2 |
| Youngerman , BE et al. | 2020 | 32048039 | 0 | 0 | 0 | 0 | 0 | 0 |
| Massoud, EAS et al. | 2020 | 32087990 | 0 | 1 | 0 | 0 | 0 | 1 |
| Lavigne, P et al. | 2020 | 32104970 | 0 | 0 | 1 | 1 | 0 | 2 |
| Jin, B et al. | 2020 | 32180016 | 0 | 1 | 0 | 0 | 0 | 1 |
| Simal-Julián, JA et al. | 2020 | 32206530 | 1 | 0 | 0 | 0 | 0 | 1 |
| Hannan, CJ et al. | 2020 | 32318930 | 1 | 0 | 0 | 0 | 0 | 1 |
| Xue, H et al. | 2020 | 32319826 | 0 | 0 | 0 | 0 | 0 | 0 |
| London, NR et al. | 2020 | 32362077 | 0 | 1 | 1 | 1 | 1 | 4 |
| Zeden, JP et al. | 2020 | 32480371 | 0 | 0 | 0 | 0 | 0 | 0 |
| Jimenez Zapata, Hd et al. | 2020 | 32500002 | 1 | 1 | 0 | 1 | 0 | 3 |
