# Supplementary figures and images for: Skull base repair following endonasal pituitary and skull base tumour resection: a systematic review
Source: Pituitary. 2021 May 10;24(5):698–713. doi: 10.1007/s11102-021-01145-4 (PMC8416859; doi:10.1007/s11102-021-01145-4)

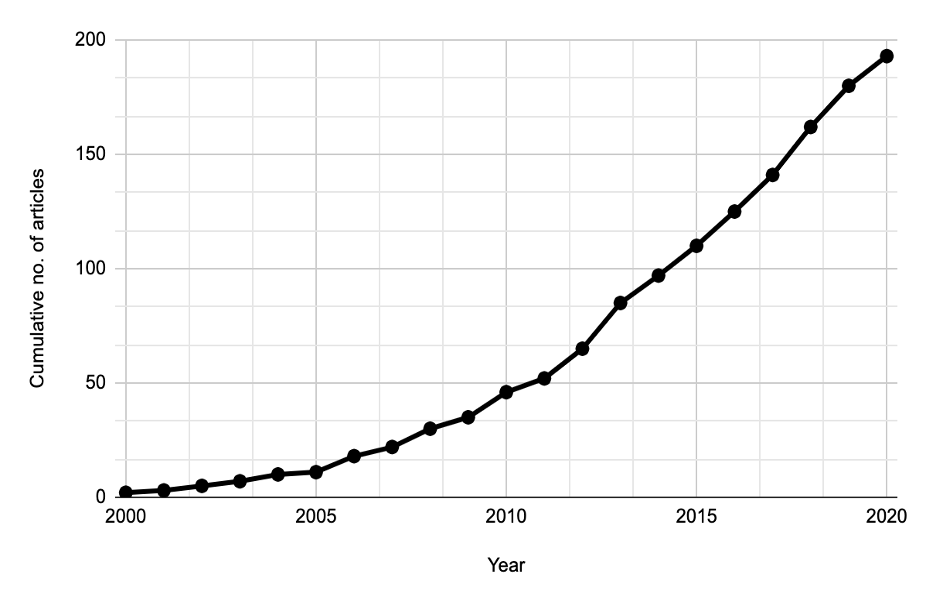

Supplement: Supplementary file 3 — Supplementary file3 (TIFF 2197 kb) Supplementary information 3: Number of studies published over time. [file 11102_2021_1145_MOESM3_ESM.tiff]
