# Supplementary material for: Skull base repair following endonasal pituitary and skull base tumour resection: a systematic review
Source: Pituitary. 2021 May 10;24(5):698–713. doi: 10.1007/s11102-021-01145-4 (PMC8416859; doi:10.1007/s11102-021-01145-4)
Supplement: Supplementary file 4 — Supplementary file4 (DOCX 136 kb) Supplementary information 4: Summary of repair protocols described per study. [file 11102_2021_1145_MOESM4_ESM.docx]

| **Authors** | **Year** | **Surgical Approach** | **Technique used (if transsphenoidal approach)** | **Study Focus** | **Skull base repair method and protocol** | **Rationale for or experience of skull base method choice** |
| --- | --- | --- | --- | --- | --- | --- |
| Ruggeri, Andrea G and Cappelletti, Marti-and Giovannetti, Filippo and Priore, Paolo and Pichierri, Angelo and Delfini, Roberto | 2019 | Both approaches described | Endoscopic | Protocol proposed. 3 repair techniques depending on risk of CSF leak. Repair for low risk: Collagen matrix, fibrin glue, autologous fat/ turbinal mucosa. Repair for medium risk: Preventive setup of nasoseptal mucosal flap Collagen matrix, fibrin glue. Repair for high risk: Preventive setup of nasoseptal mucosal flap Autologous fascia lata w/wo fat, cartilage, collagen matrix, fibrin glue, Lumbar drain for High risk ioCSFL | Patients stratified into risk of leak categories. Based on stratification they received repair A, B or C. A for low risk: Collagen matrix, fibrin glue, autologous fat/ turbinal mucosa (n=13, 19.7%). B for medium risk: Preventive setup of nasoseptal mucosal flap Collagen matrix, fibrin glue (n=23, 34.8%). C for high risk: Preventive setup of nasoseptal mucosal flap Autologous fascia lata w/wo fat, cartilage, collagen matrix, fibrin glue, lumbar drain for high risk ioCSFL (n=30, 45.5%) | Criteria for protocol based on other literature. Key parameters: size of lesion, type of lesion, size of osteodural defect, presence and type of ioCSFL leak |
| Zwagerman, Nathan T and Wang, Eric W and Shin, Samuel S and Chang, Yue-Fang and Fernandez-Miranda, Juan C and Snyderman, Carl H and Gardner, Paul A | 2018 | Both approaches described | Endoscopic | Post-op LD use in the context of high CSF rhinorrhoea risk patients (EEA, high grade ioCSFL) | Most: Collagen graft inlay, NSF (or pericranial or lateral nasal wall). Some: fascia lata, fat, collagen graft, NSF. Post-operative antibiotics x 7 days. | LD decreases CSF pressure and pulsations. Logistic regression analysis indicated that those without LD were 2.9 times more likely to have a CSF leak (OR 2.9, 95% CI 1.1–7.4, p = 0.029) than those with LD when controlling for age (p = 0.28), sex (p = 0.31) BMI (p =0.90), and any previous surgery (p = 0.67). |
| Ishikawa, Takayuki and Takeuchi, Kazuhito and Nagata, Yuichi and Choo, Jungsu and Kawabata, Teppei and Ishizaki, Tomotaka and Wakabayashi, Toshihiko | 2018 | Both approaches described | Endoscopic | Dural suturing (simple & continuous) +- graft bridges in TSA and EEA (in the context of multilayer protocol) to spare need for NSF and lumbar drain | TSA & Grade 2 leak: simple dural suturing, fat graft (abdominal), fibrin glue. EEA with small dural defect & Grade 3 leak: continuous dural suture to a fat graft bridge (dural defect too large to directly suture), LactoSorb plate, fibrin glue, +- NSF, +- Sinus balloon. EEA with moderate dural defect & Grade 3 leak: continuous dural suture to a rectus sheath graft bridge, fat graft (abdominal), LactoSorb plate, fibrin glue, +- NSF if meningioma. Ultimately, NSF was only used in 12 cases (if meningioma or planned chemotherapy/Radiotherapy)Bed rest x 1day. | Sutures provide water tight seal and if continuous, can distribute tension along course and only requires 2 knots. Disadvantages:: technically demanding and time consuming. NSF if planned chemo/Radiotherapy or if TSM or if re-op for CSF rhinorrhoea. Lumbar drains only if CSF rhinorrhoea post-op. |
| Ogiwara, Toshihiro and Nagm, Alhusain and Hasegawa, Takatoshi and Hanaoka, Yoshiki and Ichinose, Shunsuke and Goto, Tetsuya and Hongo, Kazuhiro | 2018 | Both approaches described | Both | Graded repair protocol for intra-op CSF leaks in TSA/EEA | Grade 0 or 1 leak: dural suturing of abdominal fat graft or gelatin sponge, polyglycolic acid (PGA) sheet. Grade 2, method for grade 1 with addition of mucosal flap or nasoseptal flap (NSF). Grade 3: duraplasty in fascia bridge suture closure, NSF, sinus balloon. | Dural suturing and securing grafts to dura with sutures provides tight closure. High BMI and prior endonasal surgery or cranial Radiotherapy may influence CSF leak rates and extent of repair needed. |
| Caggiano, Chiara and Penn, David L and Laws, Edward R | 2018 | Both approaches described | Endoscopic | Lumbar drains in TSA and EEA. | If an ioCSF leak was evident, the skull base defect was reconstructed by an abdominal fat graft. Pedicled nasoseptal flaps and fascia lata were used only in selected extended or expanded cases. | LD does not dec ioCSF leak or poCSF leak but increases LOS. |
| Kuan, Edward C and Yoo, Frederick and Patel, Pratik B and Su, Brooke M and Bergsneider, Marvin and Wang, Marilene B | 2018 | Both approaches described | Endoscopic | Graded repair protocol based on intra-op CSF leak | Free Mucosa Graft (FMG) for no CSF leak. Fat graft & FMG +- rigid fixation (septal bone or Resorb-X) for low-grade leaks. Fat graft & NSF +- rigid fixation for high-grade leaks. +- Foley catheter x 3d. | Fat graft fills dead space - inlay. If Dura under high tension or thinned, use rigid buttress to support fat graft (sutured to fat graft if necessary) - trimmed to fit into bony defect. Onlay with FMG (no/low flow leaks) or NSF (high flow leaks). Foley if high flow leak and rigid buttress was felt not to be fully secure (skull base anatomy, etc). In comparing FMG versus NSF reconstruction for low-grade leaks, there was no difference in postoperative CSF leak rates (p > 0.05), Role and decision making for rigid buttress and foley catheter is subjective and unclear. |
| Li, Zhen and Ji, Tao and Huang, Guo-Dong and Guo, Jian and Yang, Ji-Hu and Li, Wei-Ping | 2018 | Both approaches described | Endoscopic | Multilayer reconstruction w or w/o NSF in EEA with defects/leaks | ‘‘Sandwich’’ technique: underlay (artificial dura mater or fascia lata), Fat graft, onlay (artificial dura mater or fascia lata), + - bone (septal) buttress. For patients underwent EEA, in which intraoperative CSF leak was expected, ‘‘sandwich’’ plus a standard NSF. All pts nasal packing with absorbable gelatin sponge and iodoform gauze. | NSF + sandwich used in 55.6%, sandwich 44.4%. No significant differences in repair techniques for post op CSFR. |
| Fnais, Naif and Maio, Salvatore Di and Edionwe, Susan and Zeitouni, Anthony and Sirhan, Denis and Valdes, Constanza J and Tewfik, Marc A | 2017 | Both approaches described | Endoscopic | Adaptations to the Hemi-transeptal Flap Technique: storing NSF at the internal nasal valve with a suture to keep it out of the way for endonasal instruments. | Large defect & high flow (i.e. EEA): fat (thigh) graft, 2 layer fascia (inlay and onlay), right posterior septal mucosal flap. If very large: traditional NSF used. For small defects and low flow (i.e. TSA) hemi T NSF alone +- fat (thigh) | Operative time was shorter using the Hemi-T technique (180.51 +- 56.9 vs. 202.9 +- 62 minutes; p 0.048). The rates of nasal morbidity (septal perforation [5/102 vs. 6/37; p 0.029] and mucosal adhesion [11/102 vs. 10/39; p 0.027]), fascia lata harvest (21/100 vs. 18/39; p 0.0028), and postoperative CSF leak rates (7/100 vs. 9/38; p 0.006) were lower in the Hemi-T group. |
| Soldatova, Liuba and Campbell, Raewyn G and Elkhatib, Ahmad H and Schmidt, Thomas W and Pinto, Nelson R and Pinto, Jaime M and Prevedello, Daniel M and Ditzel Filho, Leo F and Otto, Bradley A and Carrau, Ricardo L | 2017 | Both approaches described | Endoscopic | Leukocyte-enriched platelet-rich fibrin (L-PRF) | 10-20ml of autologous blood processed into 1mm membranes within 2hrs. Repair protocol: Collagen matrix or L-PRF membranes for the intradural (inlay), mucoperiosteal graft (TSA) or EEA. LPRF at edges of flap/graft. Nasopore and Nasal Sponge packing. |  |
| Dehdashti, Amir R and Stofko, Douglas and Okun, Jessica and Obourn, Chelsea and Kennedy, Thomas | 2016 | Both approaches described | Endoscopic | Graded repair protocol for eTSA and EEA | Grade 0: Surgicel, Evicel, and Gelfoam. Grade 1: Surgicel and Evicel, Alloderm covered by a thin layer of Evicel, multilayers of Gelfoam and Evicel +- LD. Grade 2: Avitene wrapped in Surgicel over leak site, intrasellar fat graft followed by Alloderm buttress additional peripheral fat grafts, Evicel and Gelfoam, and lumbar drain placement +- LD. Grade 3 (usually EEA/non-pituitary): Duragen, fat graft, Alloderm, Evicel, and posterior nasoseptal flap. Lumbar drain for high flow cerebrospinal fluid exposures (and foley 14F x 1 day). Overall, there were 49 type 0 42 type I, 39 type 2 and 50 type 3 reconstructions. | LD intra-op but pre procedure if: expecting high flow (e.g. opening third vent for operation). LD intra-op but post-procedure: persisting ioCSF leak despite intra-op repair, inadequacy of the harvested NSF (size, shape, or position), unexpected opening of a major cistern, or concerning patient-specific factors such as obstructive sleep apnoea. Grade 2 repairs initially gave high CSF rhinorrhoea rates so LDs added to it later on in series. Advocate for type 3 (NSF + LD) recon for high flow CSF leak. Consider rescue flap if unanticipated high flow leak. |
| Nix, Paul and Tyagi, Atul and Phillips, Nick | 2016 | Both approaches described | Endoscopic | Graded repair protocol for eTSA and EEA based on bony defect size and location | Ethmoid roof <1cm Fat plug plus free mucosa graft. Ethmoid roof >1cm inlay facial Lata plus onlay nasoseptal flap. Planum Sphenoidale inlay fat and facia lata, onlay fat ring seal, nasoseptal flap, absorbable packing, +- cartilage buttress if >3cm defect, +- BSRG packing if large defect x 3-5days. Lateral sphenoid Fat plug plus nasoseptal flap, absorbable packing. Pituitary duragen, inlay facia onlay nasoseptal flap, absorbable packing. Clivus Fat plug, onlay facia lata with onlay nasoseptal flap. All NSF were glued (Tiseel). All packs were nasopore. | highest rate of primary skull base repair failures correlates with defects over the clivus – in 2 of 5 (40%) cases – and the planum sphenoidale – in 7 of 20 (35%) cases. In the case of the clivus, the defect is typically large and involves high-flow CSF. In the case of the planum sphenoidale, the optic chiasm is exposed - there is no intracranial structure to keep the graft pushed in place while healing occurs - this is probably the reason why this type of CSF defect is difficult to repair. Thus, now using the five ‘‘F’’ closure technique – Fat, Fascia, Fat, Flap and Fragmentable pack, from intradural to extradural for these defects. |
| Horiguchi, Kentaro and Nishioka, Hiroshi and Fukuhara, Noriaki and Yamaguchi-Okada, Mitsuo and Yamada, Shozo | 2016 | Both approaches described | Both | new multilayer reconstruction using nasal septal flap combined with fascia graft dural suturing for high-flow cerebrospinal fluid | Dural defects repaired with fascia graft with 6-0 nylon sutures. NSF, fibrin glue. Fat grafts, gelatin sponge. Nasal packing x 3-5 days. Prophylactic IV Antibiotics x 3 days. +- LD post-op if high flow CSF leak |  |
| Hara, Takuma and Akutsu, Hiroyoshi and Yamamoto, Tetsuya and Tanaka, Shuho and Takano, Shingo and Ishikawa, Eiichi and Matsuda, Masahide and Matsumura, Akira | 2015 | Both approaches described | Endoscopic | Graded repair protocol with fat/fascia graft suturing to dura (sliding lock technique) as an alternative to hard buttress / gasket seal / lumbar drain | Grade 1 + 2: fat graft with dural suture anchor. Sphenoid sinus mucosal flap + fibrin glue and surgicel. Gelfoam to leaking point. Beschitin-F sponge with gauze packing x3-7d \| Grade 3: Fascia inlay sutured to dura, onlay nonsutured fascial grafts. NSF + fibrin glue and surgicel. Beschitin-F sponge with gauze packing x3-7d. 6-0 Prolene, sliding-lock-knot technique. | Sutured graft can be used as a sub to hard buttress/gasket seal and lumbar drain (especially in HIGH FLOW) CSF leak: when defect traversed 2 separate geometric planes (e.g., in cases of a transplanum transtuberculum approach), when no bony edge to secure buttress against, means no hard buttress material is pressuring anatomy. Disadvantages:: tech challenging (sliding-lock-knot technique makes tying easier with endoscopic surgery), operative time, steep learning curve. |
| Park, Jae-Hyun and Choi, Jai Ho and Kim, Young-Il and Kim, Sung Won and Hong, Yong-Kil | 2015 | Both approaches described | Endoscopic | Graded repair protocol depending on CSF leak grade and response to Valsalva when repair in place | Grade 0: surgicel,. Grade 1: Surgicel + Duraform, septal buttress, mucosa (sphenoid). Grade 2: Surgicel + Duraform + Tachocomb, septal buttress, mucosa (sphenoid) or +- rescue NSF if ioCSF leak on Valsalva despite the repair, duraseal. Grade 3: Surgicel + Duraform x 2 layers + Tachocomb, septal buttress, rescue NSF always, duraseal. | Modified classification of intraoperative CSF leaks and tailored repair technique in a multi-layered fashion using an en-bloc harvested septal bone and vascularized nasoseptal flaps is an effective and reliable method for the prevention of postoperative CSF leak. Without using any artificial grafts, abdominal fat graft, and lumbar CSF diversion, this method is considerably more convenient and successful for intraoperative CSF leaks. |
| Fiorindi, Alessandro and Gioffr√®, Giorgio and Boaro, Alessandro and Billeci, Domenico and Frascaroli, Daniele and Sonego, Massimo and Longatti, Pierluigi | 2015 | Both approaches described | Endoscopic | Use of cadaveric fascia lata in 1 to 3 layers (intradural, extradural, and extrasellar) | Fat graft (abdominal), up to three fascia lata (cadaver) layers - 2 x dural replacement and 1 graft to posterior sphenoid sinus wall, fibrin glue over. 5 patients had triple layer lata, 8 had double layer, 3 had single layer. 8 Lumbar drain for CSFR revision surgeries and ioCSFL. | Fascia lata as part of multilayer approach - effective, safe. Cadaveric to avoid donor site complications and shorten op time |
| Hayashi, Nakamasa and Mitsuya, Koichi and Gorai, Katsuya and Inoue, Keita and Ito, Ichiro and Nakagawa, Masahiro and Nakasu, Yoko | 2015 | Both approaches described | Endoscopic | Perifascial areolar tissue from the femoral/inguinal region | Ball of PAT covered with haemostatic agent (oxidized cellulose polymer) and put into sellar cavity. Another sheet of PAT was spread over the sellar floor and fixed with fibrin glue | PAT has rich vascular plexus and is flexible. Shorter surgical time than NSF as plastic surgeon can extract PAT without disturbing neurosurgical procedure. Large harvest possible, therefore good for revision surgery. Not affected in this series by post-op radiation. |
| Thorp, Brian D and Sreenath, Satyan B and Ebert, Charles S and Zanation, Adam M | 2014 | Both approaches described | Endoscopic | Use of various flaps to repair skull base defects (various) in the setting of ioCSFL | Flap focussed - used in the context of ioCSF leak. 146 NSF (52 sellar, 87 other - frontal, frontoparietal, cribiform, ethmoid, planum, lateral sphenoid, clivus) - rescue flap for TSA Pit Adenoma with ioCSFL or standard for expected larger defects, 6 endoscope assisted pericranial flap (large ant skull base defects), 1 facial artery buccinator flap (secondary options when NSF unavailable),1 inferior turbinate flap (secondary options when NSF unavailable). | Vascularized repair results in excellent success rates, especially in the setting of large defects with high CSF flow and are versatile to many defect types. |
| Hu, Fan and Gu, Ye and Zhang, Xiaobiao and Xie, Tao and Yu, Yong and Sun, Chongjing and Li, Wensheng | 2015 | Both approaches described | Endoscopic | NSF vs NSF + gasket for high flow ioCSFL in TSA and EEA | Surgicel, fat (thigh), duragen. FAT NOT USED WHEN OPEN THIRD VENTRICLE. Fascia lata graft overlay (+- gasket in gasket seal group). NSF, RAAS fibrin glue. Foley Catheter packing x 5days or nasopore. Post op (same GA) LD for all pts. 18 in NSF onlay group, 33 in NSF + gasket group. 5 cases had nasopore (all in NSF + gasket group) - iterative addition over time. Rest cases had foley Catheter packing. | Significant decrease in CSFR in NSF +gasket. Nasopore > foley: less likely to be dislodged, pulled out, doesn't burst (bony chips), more comfort for patient, provides more consistent and effective tamponade/support - not just spherical so fills dead space better. LD dec CSF pressure on the recon and allows mucosal regrowth/healing. |
| Yildirim, Ali Erdem and Dursun, Engin and Ozdol, Cagatay and Divanlioglu, Denizhan and Nacar, Osman Arikan and Koyun, Oguz Kara and Ilmaz, Adil Ery and Belen, Ahmed Deniz | 2013 | Both approaches described | Endoscopic | Autologous fibrin sealant in eTSA, EEA | fat (thigh), fascia (lata) as dural replacement, Surgicel, Autologous Fibrin Sealant over. Foley Catheter. LD in obese patients or if opened third vent. AFS is made from 120ml blood, takes 30min to produce 5-6ml AFS. | Adv of AFS: effective as part of multilayer recon, autologous, cheap, does not effect post op MRI, can spray out of a stick like applicator (vivostat), changes colour to white as polymerises to allow visualisation of applied areas |
| Xuejian, Wang and Fan, Hu and Xiaobiao, Zhang and Yong, Yu and Ye, Gu and Tao, Xie and Junqi, Ge | 2013 | Both approaches described | Endoscopic | Nasal pedicled flap using collagen matrix inlay and fascia Lata or abdominal fat onlay. | Collagen matrix inserted as inlay, followed by fascia lata or abdominal fat onlay, followed by NSF with fibrin glue used to seal around the flap edges (gasket seal technique used in 2 patients) | NSF prevents host tissue reaction , cheap, malleable and safe. NSF accelerated healing but alone does not have the strength needed hence multilayer technique essential. |
| Patel, Kunal S and Komotar, Ricardo J and Szentirmai, Oszkar and Moussazadeh, Nelson and Raper, Daniel M and Starke, Robert M and Anand, Vijay K and Schwartz, Theodore H | 2013 | Both approaches described | Endoscopic | -Sellar pathology: For <2.5cm size or <1cm suprasellar extension: no CSF leak receives Gelfoam + Medpor buttress inlay + duraseal. If CSF leak then gelfoam replaced with autologous fat. For >2.5cm or >1cm suprasellar extension lumbar drain inserted and NSF prepare: if CSF leak: autologous fat then medpor buttress then NSF then duraseal. If no CSF leak then gelfoam instead of fat. -Extradural pathology: If no CSF leak gelfoam + duraseal. If CSF leak then fat + gelfoam + duraseal. -Intradural nonsellar pathology: gasket technique with fascia lata inlay then medpor buttress then uni or bilateral NSF then duraseal plus lumbar drain. All patients in all groups were kept in bed for 24 hours with bed at 30deg. | Algorithm diagram in paper. For sellar lesions: If no CSF leak: gelfoam + Medpor buttress inlay covered with duraseal. If low flow leak then gelfoam is replaced with fat. In patients with >2.5cm tumour or >1cm suprasellar extension then NSF added after medpor and lumbar drain used. Choice of gelfoam or fat is dependant on presence of CSF leak or not. For extradural lesions: Gelfoam + Duraseal if no CSF leak or if low flow leak then autologous fat + gelfoam overlay + duraseal. For intradural non-sellar lesion: gasket seal technique: fascia lata inlay buttressed by medpor covered by uni or bilateral NSF then duraseal. All patients kept in bed for 24hrs at 30deg. | Algorithm was based on risk of intraoperative CSF leak. Main factors authors argue for are tumour size, suprasellar extension and location of pathology especially intradural nonsellar pathology |
| Eloy JA, Choudhry OJ, Friedel ME, et al | 2012 | Both approaches described | Endoscopic | Comparing post-op rates of CSF leak with or without tissue sealant use | NSF harvested early in high flow anticipated cases. Sphenoid sinus mucosal edges denuded (prevent mucocele and improve adhesion of NSF). High flow converted to low flow with one of: gelfoam OR autologous fat graft +/- porex implant OR acellular dermal allograft OR fascia lata OR pericranium OR duragen. NSF then rotated onto defect. Surgicel then around the edges. Tissue sealant then applied in some patients (duraseal or fibrin glue) depending on surgeon performing procedure. then multiple pieces of gentamicin soaked gelfoam and buttressed with merocel nasal tampon covered in bacitracin ointment. Antibiotics until nasal tampons removed (usually day 10). Use 3rd gen cephalosporin OR penicillin + betalactamase inhibitor. Stool softeners and advice to avoid straining, blowing nose, or any 'activity that raises ICP'. Lumbar drain not used routinely. | Aim of paper is to assess if adding tissue sealant affected post-op CSF leak. Conclusion: no significant difference in CSF leak rates (however I note that pathology types in the two groups are quite different with maybe less invasive pathology types in the non-tissue sealant group) |
| Eloy JA, Kuperan AB, Choudhry OJ, et al | 2012 | Both approaches described | Endoscopic | Use of NSF without lumbar drainage | Same authors as 106. Very similar reported technique. NSF raised early. Size depends on anticipated defect size (e.g. clival defects may require smaller NSF). Defect mucosal edged denuded to prevent mucocele and improve NSF adhesion. High flow then converted to low flow with autologous fat graft OR Gelfoam OR fascia lata OR acellular dermal allograft (LifeCell Corporation) OR DuraGen. NSF then applied over defect. Surgical around NSF edges. Some cases have additional tissue sealant. Several pieces of gentamicin soaked gelfoam to support then buttressed by merocel nasal tampon covered in bacitracin ointment. Abx with 3rd gen cephalosporin OR penicillin + betalactamase inhibitor for 10 days or until merocel nasal tampon removed. High CSF activities advised to be avoided including heavy lifting, straining, leaning forwards, nose blowing. Stool softeners given. Advice to sneeze with mouth open. Kept in bed for 24hours. | Authors describe that reported rates of CSF leak with or without lumbar drainage varies in literature. They add their series as an example that post op CSF leaks can still be prevented even without use of lumbar drainage. They highlight risks of lumbar drainage |
| Learned, Kim O and Adappa, Nithin D and Loevner, Laurie A and Palmer, James N and Newman, Jason G and Lee, John Y K | 2013 | Both approaches described | Endoscopic | NSF assessment using MRI enhancement carried out within 48hours post-op - predictor of outcome and CSF leak | In 26/28: inlay autologous fat + duragen +/- fascia lata + NSF onlay. 2/28 had onlay NSF only. 6 patients had B/L NSF raised (total of 34 flaps). In 3, NSF could not reach anterior edge of cribriform, defect filled with free mucosal graft. | 3 cases of post-op leak found when MRI findings were: incomplete defect coverage in radiologically visible bilateral NSF, displacement of visible bilateral NSF, non-visible NSF on MRI |
| McCoul, Edward D and Anand, Vijay K and Singh, Ameet and Nyquist, Gurston G and Schaberg, Madeleine R and Schwartz, Theodore H | 2014 | Both approaches described | Endoscopic | Group A: high flow leak with NSF, Group B: NSF not used and during time period after this team adopted NSF, Group C: all patients before adoption of NSF | Perioperative Cefazolin for extra-arachnoidal tumours/encephaloceles, triple antibiotics therapy if larger defect. NSF harvest before resection if grade 3 leak anticipated, intradural tumors such as meningiomas, craniopharyngiomas, and chordomas with intradural extension as well as large mac- adenomas that extend >1 cm above the jugum sphenoidale. Closure for pit tumours: fat graft to fill empty sella, medpor then NSF then duraseal layer. For larger intradural tumours: fat to fill intracranial cavity, then gasket seal (fascia lata onlay slightly larger, supported by inlaid medpor) then NSF then duraseal. Nasal packing with telfa sponge in each nostril. Fat graft used in 61, gasket seal in 31, |  |
| Chung, Sang-Bong and Nam, Do-Hyun and Park, Kwan and Kim, Jong Hyun and Kong, Doo-Sik | 2012 | Both approaches described | Endoscopic | Use of hydroxyapatite (HA) cement as asellar repair mechanism | fascia lata or dural substitute (Lyoplant), posterior nasal septum buttress if ioCSFL, fibrin glue, HA cement (Hydroset®) graft. Bed rest x 2-3 days if high flow ioCSFL. | Adv: mouldable/adaptable, can be seem in MRI well Disadvantages:: expensive. |
| Kim, Sejin and Jeon, Chiman and Kong, Doo-Sik and Park, Kwan and Kim, Jong Hyun | 2011 | Both approaches described | Endoscopic | Use of radiation-sterilized allografts (iliac bone and fascia lata from cadavers) for sellar recon TSA | Fascia lata onlay, iliac bone buttress to gasket seal. Fascia lata, gelatin foams, collagen sponges, DuraSeal | Adv: decrease op time, donor site morbidity, septal bone is irregularly shaped. Disadvantages:: infection risk from donor (therefore gamma irradiation and cultures sent of allografts pre insertion), radiation damage to grafts - integrity and adhesion. Recommend use in no to low flow ioCSF leak for this reason. |
| Bergsneider, Marvin and Xue, Kai and Suh, Jeffrey D and Wang, Marilene B | 2012 | Both approaches described | Endoscopic | Barrier-limited multimodality reconstruction for eTSA and EEA | Barrier-limited multimodality technique: Collagen sponge (helistat), clipped (titanium - teleflex) to the dura; Fat graft (abdominal) under pressure from buttress (ethmoid, vomer OR titanium plate - Stryker OR Resorb X); NSF, Duraseal, Nasopore, Cuday balloon cath. This is compared vs NSF only. As experience and confidence in BLMM grew other time, the use of ELDs for ioCSL decreased over time to 0. | Clips need dural cuff and don't provide waterproof closure with the gelatin sponge. Clips can produce MRI artefact. BLMM can be tested with Valsalva pre NSF application. BLMM may spare LD use. the membrane barrier allows the use of a smaller fat autograft and prevents the creation of an iatrogenic mass effect resulting from the graft; (3) the membrane barrier can help support a prolapsed arachnoid diaphragma and therefore reduce the risk of delayed rupture; (4) the creation of the barrier-limited construct is not technically challenging(as opposed to described suturing techniques)the construct avoids the placement of a large fat graft within the sphenoid sinus, which can be malodorous as it decays; and (7) it appears to eliminate the need for ELDs and therefore should shorten hospital stays. The technique is not applicable in all cases, particularly if the bony defect is too large to adequately secure a buttress. The fat based mechanical component is non-vascularized and therefore theoretically prone to infection-related wound-healing complications. |
| Patel, Mihir R and Stadler, Michael E and Snyderman, Carl H and Carrau, Ricardo L and Kassam, Amin B and Germanwala, Anand V and Gardner, Paul and Zanation, Adam M | 2010 | Both approaches described | Endoscopic | Vascular skull base repair in eTSA and EEA sep on ioCSF and defect location | Generally, if no ioCSFL - mucosal graft or fat graft or no repair. If no ioCSFL + thinned diaphragm or dura subject to stress (radiotherapy, etc) = use vascular flaps. If low flow ioCSFL = transellar (fat, vascular tissue), anterior SB (vascular, fascia graft). if high flow ioCSFL = sellar (fat in sphenoid, vascular flap), clival (fat, vascular), anterior SB (fascia, vascular). Use vascular flaps with location of defect in mind -> If available, use NSF. If NA, then use pericranial (anterior skull base), temporoparietal fascia flap (TPFF) or inferior turbinate flap (posterior/clival). NSF in 150 patients. 10 PCF, 2 TPFF, 4 ITF. | **Flap taxonomy:** Intranasal (NSF, ITF, MTF), Regional (PCF, temp-parietal, palatal flap). NSF (on posterior nasoseptal/sphenopalatine artery) = ideal and versatile. ITF (inferior turb artery) = good for clival defects but cant reach anteriorly. MTF (on middle tub artery) = good for sellar or anterior but small, thin and difficult to elevate. PCF (supraorbital/supratrochlear artery) = robust, versatile but limited to sella and anterior. TPF (on sup temp artery) = parasellar, clival but not anterior. Palatal (on greater palatine artery) = difficult to elevate, versatile with large pedicle. |
| Kong, Doo-Sik and Kim, Hyo Yeol and Kim, Se-Hwan and Min, Jin-Young and Nam, Do-Hyun and Park, Kwan and Dhong, Hun-Jong and Kim, Jong Hyun | 2011 | Both approaches described | Endoscopic | Graded repair (based on ioCSFL) in eTSA and EEA - looking at no grafting, free grafting vs gasket sealing | No ioCSFL: surgicel or avitene or gelfoam. G1 and 2: fat (abdominal), mucosa (sphenoid, middle turb). G1+2: gasket seal with fascia lata and bone buttress (cadaver - iliac), fascia lata, gelfoam, collagen sponge, duraseal. G2 = LD x 5 days. Results: No tissue graft 20; Fat and mucosal graft 58; Gasket-seal method using fascia 46. In the 78 patients with grade 0 CSF leak, 20 received simple closure without tissue graft, 17 received gasket-seal method, and 41 received a free-fat–mucosal graft. In the 20 patients with grade 1 CSF leaks, eight received a free-fat and mucosal graft and 12 received the gasket-seal method. In 26 Grade3: 8 fat + mucosa, 17 gasket seal | Comparison of reconstructive methods revealed that gasket-seal method provided better control of CSF leaks than free-fat graft in patients with grade 2 leaks (11.8% vs. 66.7%, p=0.028). However, in grades 0 and 1, we found no difference among the various reconstructive methods. Degree of intraoperative CSF leak alone was closely associated with persistent CSF leak (p<0.001). Gasket seal - adv: spares nasal morbidity (grafts, flaps); disadvantages: Radiotherapy history, large defects extending to parasellar - e.g. with OGMs. |
| Sciarretta, V and Mazzatenta, D and Ciarpaglini, R and Pasquini, E and Farneti, G and Frank, G | 2010 | Both approaches described | Endoscopic | Algorithm depending on visible diaphragma defect and visible CSF leak. Three groups: gelfoam (+abdominal fat if diaphragma visibly thin, but no leak present), abdominal fat/mucoperiosteum flap (for small leak), multilayer repair including fascia lata + abdominal fat + bone/mucoperiosteum | Algorithm described. Group1-No CSF leak: Gelfoam packing +/- abdominal fat (529 patients had gelfoam, 8 had additional abdominal fat due to visible thin diaphragma but no visible leak). Group2-small defect or oozing: abdominal fat + sphenoid mucosa+/-mucoperiosteal flap from middle turbinate generally (123 patients). Group3-large diaphragmatic defect or CSF leak: fascia lata underlay, abdominal fat next, then bone/mucoperiosteal flap as underlay. Spongostan to pack sphenoid sinus. Merocel tampon nasal packing (5 patients) | Algorithm takes into account complex defects are best repaired with multilayer using autologous tissues as it is easier. Failure of repair found (on questioning patients) to be related to non-adherence to anti-high ICP guidance and/or coughing on emergence from anaesthesia. |
| Horiguchi, Kentaro and Murai, Hisayuki and Hasegawa, Yuzo and Hanazawa, Toyoyuki and Yamakami, Iwao and Saeki, Naokatsu | 2010 | Both approaches described | Endoscopic | Non-Flap group: fat or fascia lata packing into sphenoid sinus | Fat or fascia lata packing into sphenoid sinus. Most patients in this group received a pre-op LD | This was the groups old technique. |
| Horiguchi, Kentaro and Murai, Hisayuki and Hasegawa, Yuzo and Hanazawa, Toyoyuki and Yamakami, Iwao and Saeki, Naokatsu | 2010 | Both approaches described | Endoscopic | Flap group: use of NSF as well as 'sinus balloon' | NSF placed over dural defect and held in place with fibrin tissue glue. No intradural grafts. Fat graft over NSF as support. 'Sinus balloon' placed under this as support (sinus balloon is the authors design- essentially a foley catheter balloon with a shortened tip - image in paper). Prophylactic antibiotics given for 7-10 days (duration of sinus balloon placement). ELD placed if post-op CSF leak. | New technique adopted by this group. Adoption of NSF has reduced (in their analysis) rates of post-op CSF leak and use of peri-operative LD. |
| Couldwell, William T and Kan, Peter and Weiss, Martin H | 2006 | Both approaches described | NR | No routine closure of sella | For small leak, abdominal fat alone in sphenoid sinus. For larger leaks, abdominal or lata fascia over dura -> abdominal fat in sphenoid sinus -> surgicel sling. | Routine closure of sella (without CSF leak) is unnecessary, which avoid disadvantages of autologous or synthetic grafts |
| Couldwell, William T and Kan, Peter and Weiss, Martin H | 2006 | Both approaches described | NR | No routine closure of sella | For small leak, abdominal fat alone in sphenoid sinus. For larger leaks, abdominal or lata fascia over dura -> abdominal fat in sphenoid sinus -> Marlex mesh. | Routine closure of sella (without CSF leak) is unnecessary, which avoid disadvantages of autologous or synthetic grafts |
| Couldwell, William T and Kan, Peter and Weiss, Martin H | 2006 | Both approaches described | NR | No routine closure of sella | Where no CSF leak for small lesions, only single later of surgicel over the arachnoid layer and the remaining pituitary gland. | Routine closure of sella (without CSF leak) is unnecessary, which avoid disadvantages of autologous or synthetic grafts |
| Husain, Qasim and Sanghvi, Saurin and Kovalerchik, Olga and Shukla, Pratik A and Choudhry, Osamah J and Liu, James K and Eloy, Jean Anderson | 2013 | Both approaches described | Endoscopic | Reducing mucocele formation when using NSF over mucosa grafts | Denude mucosa around defect +- at sphenoid sinus. NSF, surgicel over +- (dural sealant and gelfoam soaked in gentamicin). Merocel (covered in bacitracin) x 10days. Antibiotics until packs removed. Stool softeners. | Judicious mucosal denuding around the SB defect and meticulous closure can minimize this potential risk of mucocele formation after PNSF repair of ventral SB defects. |
| Otto, Bradley A and Bowe, Sarah N and Carrau, Ricardo L and Prevedello, Daniel M and Ditzel Filho, Leo F and de Lara, Danielle | 2013 | Both approaches described | Endoscopic | Modified rescue NSF in eTSA and EEA | G1/2: collagen matrix, fat (abdominal) or mucoperiosteal (unspecified), NSF. 44 bilateral (modified rescue NSF), 4 bilateral (1 trad NSF, 1 modified rescue NSF), 16 unilateral modified rescue NSF. | A) Standard rescue NSF: use of the posterior-superior limb of the NSF incision, followed by the inferior reflection and retraction of the pedicle. B) Modification include inferior incisions: "pedicle sparing-transposition technique" (shifting the pedicle inferiorly and a more horizontal position). Adv: modification allows for the dissection of the inferior sphenoid sinus and clival recess owing to the increased mobility and more horizontal orientation of the pedicle - allows trasclival etc approach and keeps pedicle away from damage/surgical corridor. By elevating only the posterior extent of the NSF, we still capitalize on decreased donor site morbidity. |
| Hannan CJ, Almhanedi H, Al-Mahfoudh R, Bhojak M, Looby S, Javadpour M. | 2020 | Both approaches described | Endoscopic | Risk factors for poCSFR in eTSA and EEA | Fat, tissue glue, NSF, tissue glue. If high flow ioCSFL: fascia lata to dura, NSF, tissue glue | Previous surgery, craniopharyngiomas, adenomas causing Cushing’s disease (OR 5.79, 95% CI 1.53–21.95, p = 0.01) and intra-operative CSF leaks (OR 4.56, 95% CI 1.56–13.32, p = 0.006) were associated with an increased risk of post-operative CSF leak. The use of a vascularised nasoseptal flap and increasing surgical experience (OR 0.14, 95% CI 0.04–0.46, p = 0.001) were associated with a decreased rate of CSF leak. |
| Simal-Julián JA, Miranda-Lloret P, P√©rez de San Rom√°n Mena L, Sanrom√°n-√Ålvarez P, Garc√≠a-Pi√±ero A, Sanchis-Mart√≠n R, Botella-Asunci√≥n C, Kassam A. | 2020 | Both approaches described | Endoscopic | Monolayer vs multilayer with vascular flap repair in eTSA and EEA | Monolayer (n=65): duragen underlay, vascular flap, tissue glue, foley +- LD (n=21). Multilayer (n=36): fat to dead space, fascia lata graft, vascular flap, tissue glue, foley. Vascular flap: Nasoseptal 84/101, middle turbinate 5/101, extended inferior turbinate 6/101, pericranial transglabelar 3/101, “U” inverted rhinopharyngeal flap 3/101 | Inc risk of poCSFR with: patients older than 45 years, non-PAs, EEA (especially transplanum), "monolayer" recon, LD (in the context of monolayer), high flow ioCSFL (especially 3rd Vent opening). The reconstructive protocol employed in each case is an independent risk factor for presenting postoperative leak. The multilayer protocol has an intradural phase (packing dead space and creation of watertight seal), second phase addresses the bony defect and supports intradural phase, this phase is nasal and involves placing vascular flap to seal |
| London NR Jr, Mohyeldin A, Montaser AS, Tanjararak K, Prevedello DM, Otto BA, Carrau RL. | 2020 | Both approaches described | Endoscopic | **Risk factors** for Delayed CSFR after TSA/EEA (>1 week post-op) | Collagen matrix inlay/gasket seal 82.4% (14 of 17). Vascularized flap 76.5% (13 of 17), Nasoseptal flap 9 of 13, Pericranial flap 2 of 13, Buccal fat flap 1 of 13, TPPF 1 of 13, Free mucosal graft 11.8% (2 of 17), Fat graft 41.2% (7 of 17), | 14 of 17 delayed CSFR were EEA. The most common reconstruction during the initial surgery consisted of an inlay or gasket seal collagen matrix (82.4% of patients) with an onlay pedicled flap (76.5% of patients). Identifiable causes included dehiscence of the nasoseptal flap (17.6%); a provoking event such as emesis, sneezing, or fall (17.6%); partial or total necrosis of the flap (11.8%); displacement of local flap (11.8%); and inadequate apposition of the flap to the surfaces around the defect, that is, folded flap (11.8%). Re-repair with fat graft and/or collagen sponge and/or flap replacement/repositioning often successful. |
| Conger, Andrew and Zhao, Fan and Wang, Xiaowen and Eisenberg, Amalia and Griffiths, Chester and Esposito, Felice and Carrau, Ricardo L and Barkhoudarian, Garni and Kelly, Daniel F | 2018 | Both approaches described. EEA in 41%, TSA in 59%. | Endoscopic | Graded repair protocol based on intra-op CSF leak | Grade 0: Intrasellar fat graft (abdominal), Collagen sponge on-lay, sphenoid sinus mucosa, Collagen sponge, tissue glue. Grade 1: Intrasellar fat graft, Collagen sponge on-lay, buttress (bone or Medpor), sphenoid sinus mucosa, Collagen sponge, tisseel. merocel x5d. Grade 3: Intrasellar fat graft, Collagen sponge on-lay, buttress (bone or medpor), sphenoid sinus mucosa, fat in sphenoid, Collagen sponge, tisseel. merocel. Grade 3: Intrasellar, suprasellar, &/or clival fat graft, collagen sponge overlay, buttress (bone or medpor), NSF, fat, collagen sponge, tisseel, binostril merocel x5. Prophylactic Antibiotics x 5days. Fat grafts were used in 33%, 84%, 97%, and 100% of grade 0, 1, 2, and 3 leaks, respectively. Pedicled mucosal flaps (78 total) were used in 2.6% of grade 0–2 leaks (combined) and 79.5% of grade 3 leaks (60 nasoseptal and 6 middle turbinate flaps). Nasoseptal flap usage was highest for craniopharyngioma operations (80%) and lowest for pituitary adenoma operations (2%). | More buttress (permanent bone/medpor or temp packing), less LD, more Antibiotics = dec CSFR and meningitis. More NSF for high flow leaks. |
| Geltzeiler, Mathew and Nakassa, A-Caroli-Igami and Turner, Meghan and Setty, Pradeep and Zenonos, George and Hebert, Andrea and Wang, Eric and Fernandez-Miranda, Juan and Snyderman, Carl and Gardner, Paul | 2018 | Expanded endonasal (anything beyond accessing the sella alone) | Endoscopic | Evaluation of NSF viability using intra-op ICG green fluorescence angiography | 24 NSF, 11 Extended NSF, 2 Lateral Nasal Wall Flap, 1 reused flap | NSF viability with ICG correlates with post-op MRI contrast enhancement. Useful tool to predict intra-op if flap necrosis likely and therefore revision |
| Nyquist, Gurston G and Anand, Vijay K and Singh, Ameet and Schwartz, Theodore H | 2010 | Expanded endonasal (anything beyond accessing the sella alone) | Endoscopic | Bilateral NSF | Janus flap +/- fat graft(2), fascia underlay (1), gasket (2), vomer (1) | Bilateral (or Janus) flap has utility for large skull base defects. May also be used if other side of septum will be sacrificed anyways |
| Wardas, Piotr and Tymowski, Micha≈Ç and Piotrowska-Seweryn, Agnieszka and Markowski, Jaros≈Çaw and ≈Åadzi≈Ñski, Piotr | 2019 | Expanded endonasal (anything beyond accessing the sella alone) | Endoscopic | NSF (Haddad-Bassagasteguy flap) | Layered: rectus sheath + posterior septectomy bone harvest + fibrin sealant + NSF | Technical points on how to raise NSF are mostly the bulk of discussion. Absolute indications for NSF harvesting prior to resection are: reoperations in the case of a previous open approach, preoperative CSF leakage, intradural localization of a tumour related to its aetiology- pathogenesis, and suspicion of intradural diffusion of a neoplasm in MRI if the etiopathogenesis cannot clarify the tumor’s relation to the meninges (mainly chordoma). Relative indications concern mostly pituitary- macroadenomas of at least 2.5 cm in diameter assessed according to the Schwartz classification and localization |
| Singh, C and Shah, N | 2019 | Expanded endonasal (anything beyond accessing the sella alone) | Endoscopic | Posterior NSF | NSF | Cons of NSF not otherwise discussed elsewhere in reading: loss of smell, importance of counselling patients re this risk |
| Kessler, Remi A and Garzon-Muvdi, Tomas and Kim, Eileen and Ramanathan, Murugappan and Lim, Michael | 2019 | Expanded endonasal (anything beyond accessing the sella alone) | Endoscopic | NSF | NSF 19, fat graft 12, lumbar drain 7, free middle turbinate graft 3 | Vascularised reconstructions consistently superior to non-vascularised. |
| Cavallo, Luigi M and Solari, Domenico and Somma, Teresa and Cappabianca, Paolo | 2019 | Expanded endonasal (anything beyond accessing the sella alone) |  | Combination of fat, fibrin, flap and fast mobilisation | 1st layer (fat graft (abdominal) to fill skull base defect), fibrin glue to hold, check with Valsalva, 2nd layer (NSF). Merocel x 3 days. No lying flat x 2 weeks and early mobilisation. Antibiotics x 5 days post op. | Dural/bony defect/dead-space is very variable in EEA and irregular in multiple dimensions, thus using a flex/mouldable first layer (fat graft) is desirable to plug the gap. Also this may make overpacking less dangerous (only fat would compress visual structures as opposed to a rigid graft) |
| Moon, Ju Hyung and Kim, Eui Hyun and Kim, Sun Ho | 2019 | Expanded endonasal (anything beyond accessing the sella alone) |  | Extended NSFs | Extended nasoseptal flaps for large defects (NSF with inferior turbinate mucosa, NSF with entire lateral nasal wall mucosa). First layer was collagen fleece at dura, tachosil. Flaps were covered with Bemsheets surgical pads (Kawamoto) soaked in Antibiotics and then packed with merocel (covered by finger portion of surgical glove) x 5days |  |
| Liu, James K and Mendelson, Zachary S and Kohli, Gurkirat and Eloy, Jean Anderson | 2018 | Expanded endonasal (anything beyond accessing the sella alone) |  | Modified NSF for Transcribiform EEA. Involves a relaxing slit incision at sphenoidal portion of NSF - to increase AP (from frontal sinus to planum) reach of flap and reduce tension in doing so | 1st layer: Fascia lata or acellular dermal allograft (inlay). 2nd: acellular dermal allograft (inlay). 3rd layer: NSF with relaxing slit + surgicel. Packing: gentamicin-soaked gelfoam, merocel x 10-12 days. Oral Antibiotics until discharge. | Wide defects in transcribiform EEA can put standard NSF under tension when trying to cover. |
| Cohen, Salomon and Jones, Samuel H and Dhandapani, Sivashanmugam and Negm, Hazem M and Anand, Vijay K and Schwartz, Theodore H | 2018 | Expanded endonasal (anything beyond accessing the sella alone) | Endoscopic | io Lumbar Drain insertion - effects on CSF rhinorrhoea (EEA) | Gasket seal: fascia lata + Medpore, NSF overlay, Duraseal as glue over it. All patients had ioCSF LD placement if possible. Antibiotics for duration of LD insertion. | intraoperative Lumbar Drain reduces post op CSFR in obese patients undergoing EEA (i.e. larger defects and higher flow leaks) for meningioma . |
| Jeon, Chiman and Hong, Sang Duk and Seol, Ho Jun and Lee, Jung-Il and Nam, Do-Hyun and Hwang, Yoon Jung and Kong, Doo-Sik | 2017 | Expanded endonasal (anything beyond accessing the sella alone) |  | Multi-layered non-vascularized repair vs NSF: for EEA (non-sellar tumours) | Method 1, n=38: Gasket seal with fascia lata , bone (vomer) buttress. Method 2, n=57: NSF + fascia lata, merocel, lumbar drain x 3-5d. | NSF may not be as useful or may be more difficult to apply in EEA with non-pituitary skull base tumors requiring intra-arachnoid dissection (especially posterior/posterolateral locations) |
| Munich, Stephan A and Fenstermaker, Robert A and Fabiano, Andrew J and Rigual, Nestor R | 2013 | Expanded endoscopic endonasal (anything beyond accessing the sella alone) |  | First 10 patients: autologous tissue (abdominal fat or fascia lata) and/or allograft (alloderm supplemented with duraseal and gelfoam). In the next 39 patients, a vascularised NSF was used in addition to the previous. | 39/49 - pedicled nasoseptal flap, autologous tissue graft intradural underlay, autologous tissue graft overlay | Shift to addition of NSF was due to high rate of CSF leak (20%) in first 10 patients with conventional repair method |
| Acerbi, Francesco and Genden, Eric and Bederson, Joshua | 2010 | Expanded endoscopic endonasal (anything beyond accessing the sella alone) |  | Circumferential U-clips to suture Alloderm (3) or Fascia lata (1) + fat (2/3) + multilayer technique using Tisseal then Gelfoam | Prophylactic antibiotics given to all. | Watertight closures using U-clip in other fields demonstrated. Easier use than knot tying although requires practice to speed up the process. |
| Ivan, Michael E and Iorgulescu, J Bryan and El-Sayed, Ivan and McDermott, Michael W and Parsa, Andrew T and Pletcher, Steven D and Jahangiri, Arman and Wagner, Jeffrey and Aghi, Manish K | 2015 | Expanded endoscopic endonasal (anything beyond accessing the sella alone) |  | Abdominal fat autograft between two pieces of DuraGen. NSF then added in some cases. | Duragen inlay to exposed brain. Then fat graft if used. Then Surgicel over fat graft. Fibrin sealant around the edges (Confluent surgical). NSF over this with duraseal to seal the edges. Sinus walls denuded to allow better NSF adherence. Septal splints in place over exposed septum for 3-4 weeks. Gelfoam over whole wound. Foley catheter buttress. Two merocel sponges for further buttress. LD may be used for 3-5 days draining 10-20cc/hour. Pre-op antibiotics continued if foley or nasal packing kept in. No PPV for 3 days if no leak, for 1 month if leak present. Duragen 66, Fat graft 29, NSF 85, mucosal free flap 3, vascularised muscle flap 4. | NSF reported in literature to be better for predicted high flow leaks and for confirmed high flow leaks |
| Jyotirmay, Hegde and Saxena, Sunil Kumar and Ramesh, A S and Nagarajan, K and Bhat, Shreshta | 2017 | Expanded endoscopic endonasal (anything beyond accessing the sella alone) |  | Assessing NSF viability via imaging | Nothing else mentioned besides NSF | ?MRI may be a useful assessment tool of flap viability post op in terms of location and ischaemia (but this evidence is not convincing) |
| So, Jaeyoung and Park, Hunho and Sung, Kyeong-Soo and Lee, Kyu Sung and Hong, Chang-Ki | 2017 | Both approaches described |  | Tachosil sandwich technique with porous polyethylene implant vs autologous fat graft with fibrin glue | For all patients, nasal packing removed after 2 days, no sphenoid packing used | Study comparing their conventional technique with use of TachoSil for Gr3 CSF leaks |
| Fraser, Shannon and Gardner, Paul A and Koutourousiou, Maria and Kubik, Mark and Fernandez-Miranda, Juan C and Snyderman, Carl H and Wang, Eric W | 2018 | Expanded endoscopic endonasal (anything beyond accessing the sella alone) |  | Risk factors for CSF rhinorrhoea in EEA and effect of obesity | LD and NSF for large defect or high flow ioCSFL (most of these intradural cases) | NSF associated with decreased post op CSFR rates, especially with high flow, large defects in high BMI patients |
| Yoo, Frederick and Wang, Marilene B and Bergsneider, Marvin and Suh, Jeffrey D | 2017 | Expanded endoscopic endonasal (anything beyond accessing the sella alone) |  | Using single layer synthetic/xenograft material to reconstruct large anterior skull base defect (no vascular flap) - EEA | Single layer of AlloDerm or Duramatrix as underlay to dura, fibrin glue, gelfoam, +- fat packing of dead space. Pope packs (merocel sponge placed inside of a single finger of a sterile glove and sutured at the open end), +- Nasopore sponges to paranasal sinuses, +- nasal trumpets to divert nasal airflow during the immediate postoperative period. All patients = Antibiotics x 2 weeks. | Adv of single layer: it is easily performed, reduces the load of foreign material introduced as much as possible, reduces operative procedure times, and reduces the cost of additional procedures or using multi-layered artificial reconstructive materials, can avoid potential donor site morbidity associated with mucosal grafts. AlloDerm, were subject to longer periods of postoperative crusting, increased time to re-mucosalization and recurrent infection (compared with DuraMatix) |
| Gandham, Edmond J and Sundaresan, Rajan and Thomas, Regi and Chacko, Ari G | 2017 | Expanded endoscopic endonasal (anything beyond accessing the sella alone) |  | Modified NSF use in revision EEAs: change to the superior incision posterior half (crosses midline and goes from posterior nasal septum -> nasal floor -> up to ant edge of septal defect - as opposed to posterior nasal septum to ant nasal septum up to 2cm below olfactory fossa and up to septum | Fat graft, Dural defect with fascia lata onlay, NSF, surgicel, tissue glue, and gelfoam. Bilateral merocel packs x 5 days. LD + bed rest x 5 days. | Technique modification makes it more feasible to use NSF in revision cases despite septal perforation, bilateral large sphenoidotomies. Disadvantages:: sphenopalatine pedicle must be intact, bilateral flap elevation takes time and is technically difficult. |
| Fathalla, Hussein and Di Ieva, Antonio and Lee, John and Anderson, Jennifer and Jing, Rowan and Solarski, Michael and Cusimano, Michael D | 2017 | Expanded endoscopic endonasal (anything beyond accessing the sella alone) |  | Changes in skull base repair methods over time in EEA and impact on post-op CSF rhinorrhoea | All cases same approach (not graded) - Layers: Gelfoam or fat (thigh), Fascia (lata) as dural inlay, bone (vomer) graft, NSF, tisseal. Nasal silastic septal stents, Merocel or Vaseline packing. Nasal trumpets. | The nasoseptal flap decreased the rate of CSF leak but not significantly (P = 0.112), while placing a nasal trumpet to support our repair resulted in significant decrease in CSF leak rate (P = 0.0013) |
| Thomas, Regi and Chacko, Ari George | 2016 | Expanded endoscopic endonasal (anything beyond accessing the sella alone) |  | Describing graded repair protocol for EEA based on defect size and ioCSF leak. Skull base defects were classified as (1) type I (small skull base defect), (2) type II A (sella defect without cerebrospinal fluid [CSF] leak), (3) type II B (sella defect with CSF leak), (4) type III A (EEA defect without ventricular communication), and (5) type III B (EEA defect with ventricular communication). | fascia lata underlay graft, fascia lata overlay graft, NSF (NSF +- inferior turb or middle turb), tissue glue, surgicel, gelfoam, merocel. Add: Fat graft with bath plug technique for grade 2 leaks, +- gasket seal for grade 3 leaks (with cartilage buttress + fascia lata). Post-op lumbar drain for all cases. IV Antibiotics x5day. Laxatives. | Using a fat graft (+- bath plug technique) can absorb CSF pulsations and protect overlying grafts. Adapt repair protocol to defect size, morphology and underlying leak |
| Wang, Xuejian and Zhang, Xiaobiao and Hu, Fan and Yu, Yong and Gu, Ye and Xie, Tao and Ge, Junqi | 2016 | Expanded endoscopic endonasal (anything beyond accessing the sella alone) |  | Pedicled middle turbinate mucosal flap use in EEA | Artificial dura material inlay, fascia lata and fat overlay, middle turbinate mucosal flap, tissue glue. Foley Catheter packing x 7days. Lumbar drainage x 4-6 days. Stool softeners. Avoid stress (lift, cough) | Middle turb flap - Adv: Does not need to be prepared pre-op, instead can be used as rescue PRN (The blood supply of the middle turbinate mucosal flap is located in the lateral wall of nasal cavity and is the branch of the lateral nasal artery, which should be protected during the surgery). Disadvantages:: more technically demanding than standard NSF. |
| Zenga, Francesco and Tardivo, Valenti-and Pacca, Paolo and Garzaro, Massimiliano and Garbossa, Diego and Ducati, Alessandro | 2016 | Expanded endoscopic endonasal (anything beyond accessing the sella alone) |  | Nanofibrous Synthetic Dural Patch for dural replacement in EEA | Small defects: fat graft, fibrin glue. Larger defects: Fascia Lata graft to repair dura (inlay) or synthetic dura patch (ReDura) , +- fat graft, NSF. Nasal “glove finger-fill with polyvinyl alcohol sponge” tampons for packing. | Fascia lata graft= strength, pliability, thickness, availability, and biocompatibility. However, its harvesting leads to a longer operative time and higher risk of infection, and causes an external scar. ReDura is regenerative dural repair patch made of synthetic absorbable biomaterial scaffold resembling microstructure of native dural matrix that promotes regeneration of dural cell on scaffolds. Advantages:: pliable, flexible, biocompatible, strength, dec donor morbidity, dec operative time. ReDura completely degraded in few months, reducing the risk of infection associated with foreign bodies. |
| Ishii, Yudo and Tahara, Shigeyuki and Hattori, Yujiro and Teramoto, Akira and Morita, Akio and Matsuno, Akira | 2015 | Expanded endoscopic endonasal (anything beyond accessing the sella alone) |  | Fascia patchwork closure: Fascia lata or abdominal fascia inlay patch sutured, dura sutured where possible and NSF added in some cases | New technique(32): dura sutured where possible with 5-0/6-0 nylon using deep suturing needle holders and small alligator forceps. Remaining defect filled with patch sutured fascia Lata or abdominal facia INLAY. 26 then covered with NSF. OLD technique (16): fat then NSF | - |
| Gruss, Calvin L and Al Komser, Mohammed and Aghi, Manish K and Pletcher, Steven D and Goldberg, Andrew N and McDermott, Michael and El-Sayed, Ivan H | 2014 | Expanded endoscopic endonasal (anything beyond accessing the sella alone) |  | Risk factors for CSFR in the context of EEA and use of NSF | Duragen, collagen matrix graft, +- fat graft (n=43, 36%). NSF, Duraseal. +- Fat graft over NSF at edges. Gelfoam layer. Foley Catheter + merocel packing x 10 days. Post-op lumbar drain in n=73 (60%) | Large (>2cm2) and central (sellar, clival) skull base defect are highest risk of CSRF in EEA. Central skull > anterior skull base (OR = 5.9, P = .036, 95% CI, 1.12-31.33). DURAL defect size > 2cm2 (OR = 11.4, P = .034, 95% CI, 1.20-108.29). With regard to the central skull base, the authors believe the difficulty rest in closure of a defect that lies in a vertical plane. Graft migration of the dural inlay or fat or NSF can occur due to lack of counter pressure and difficulty in securing tissue to adjacent dura. In the anterior skull base, the frontal lobe can rest on the dura, |
| Iacoangeli, Maurizio and Di Rienzo, Alessandro and di Somma, Lucia Giovan-Maria and Moriconi, Elisa and Alvaro, Lorenzo and Re, Massimo and Salvinelli, Fabrizio and Carassiti, Massimiliano and Scerrati, Massimo | 2014 | Expanded endoscopic endonasal (anything beyond accessing the sella alone) |  | Multilayer. Abdo fat intracranially. Fascia lata inlay held by fibrin flue OR larger amount of fat if no fascia lata. Then bone defect filled with bone dust + fat + fibrin glue. Mucosal/muscular layer then sutured over this. | Before procedure: I shaped incision over nasopharynx mucosa. Prevertebral muscles skeletonised in subperiosteal fashion. Intracranially: abdominal fat. Inlay: Fascia lata or more fat, held by fibrin glue. Bone defect: Abdominal fat + bone dust +fibrin glue. Muscles and mucosa of nasopharynx sutured over this after an "I" shaped incision using 3 to 4 stitches of 4-0 polysorb. Fibrin glue then applied over mucosal incision. | Use of the native mucosa/muscles is a natural vascularised flap which is easier and less harmful than other nasoseptal flaps (Note by AMSA: this is presumably more feasible over the clival region due to mucosa being more flat than sellar region). Addition of bone dust to reduce migration of fat/fibrin glue between mucosal layers. |
| Patel, Mihir R and Taylor, Robert J and Hackman, Trevor G and Germanwala, Anand V and Sasaki-Adams, Dean-and Ewend, Matthew G and Zanation, Adam M | 2014 | Expanded endoscopic endonasal (anything beyond accessing the sella alone) |  | Use of secondary flaps (i.e. NOT NSF) - several techniques described. For clival chordomas: tunnelled temporoparietal fascial flap. For 2 recurrent pituitary adenoma with suprasellar extension: inferior turbinate flap | Several flaps used. Only relevant flaps by anatomy: tunnelled temporoparietal fascial flap for clival pathology. Inferior turbinate for 2 pit adenomas (advice to use fat bolster if defect >1cm and caution to avoid obstruction of the nasolacrimal duct). 1 middle turbinate flap following failed NSF for clival pathology. | Algorithm offered: to start with NSF if possible. If not available to consider other intranasal flaps in the following order by size and accessibility (lateral anterior nasal wall > Inferior turbinate > middle turbinate). Clival/sellar defects >1cm will require dermal/fat bolster with the inf turbinate flap OR use of temporoparietal flap. Authors have stopped use of mid turbinate flap due to risk of ethmoidal air cell breech and CSF leak, failure case reported, technical difficulty of not disrupting the main pedicle (anatomy of vessels to mid turbinate described in paper) and avoiding premature bony disruption to the middle turbinate. IF intranasal tissue unavailable (primarily in this series due to intranasal Ca concerns) consider the tunnelled scalp flaps. Pericranial for anterior skull base (does not reliably reach post fossa) but temporoparietal for clival defects (but not for anterior fossa/sellar as rotation causes pedicle compromise). For anterior fossa defects, if all above fails, facial artery buccinator flap OR finally consider occipital, palatal or radial forearm free flaps. |
| Mascarenhas, Lino and Moshel, Yaron A and Bayad, Fatema and Szentirmai, Oszkar and Salek, Al Amin and Leng, Lewis Z and Hofstetter, Christoph P and Placantonakis, Dimitris G and Tsiouris, Apostolos J and Anand, Vijay K and Schwartz, Theodore H | 2014 | Expanded endoscopic endonasal (anything beyond accessing the sella alone) |  | 4 techniques reported. Fat graft only. Gasket seal. Fat graft+NSF. Gasket+NSF. | 18 had fat graft only in sella with tisseal (in their earlier years) or duraseal (2 of these had post-op leak). 24 gasket seal closure with fascia lata overlay and countersunk medpor buttress and duraseal over this (1 post op leak). 55 had fat graft+/NSF then duraseal over this (1 post op leak). 29 gasket seal + NSF and duraseal over this (0 post op leak). For pit adenomas, cefazolin. For any other intra-dural/intra-arachnoidal, triple antibiotics. sphenoid mucosa stripped so no mucocele formation under NSF. For ALL repairs, floseal in nasal cavity and finally two pieces of telfa in each nostril for 1-2days. Bed rest until 1 day post-op or 1day post-removal of lumbar drain. | Patient cohort divided into early and late halves to study learning curve. All post-op leaks occurred in early half i.e. learning curve had an effect on prevention of CSF leak. No further rationale provided for repair techniques. |
| Eloy, Jean Anderson and Patel, Amit A and Shukla, Pratik A and Choudhry, Osamah J and Liu, James K | 2013 | Expanded endoscopic endonasal (anything beyond accessing the sella alone) |  | Early harvest of NSF for cases in which high flow CSF leak is anticipated e.g. from intradural pathology, extradural pathology with dural or intradural, pituitary adenoma with large defect | NSF is prepared early with 3 main incisions along nasal floor, from sphenoid ostium towards anterosuperior and then connecting the anterior points of both incisions. Sphenoid mucosa circumferentially denuded (mucocele prevention). Multilayer closure technique then used (reported in references 36-39, 41, 43 - these references have been added to the google sheet). From figure 3 legend: First fascia lata over defect (converts high to low flow leak). Surgicel over fascia lata. Then layer of thick implantable acellular dermal allograft. Then PNSF rotated over defect, covered with surgical and gentamicin soaked Gelfoam, buttressed with an expandable nasal tampon covered with bacitracin ointment. | Early harvest allows maximal size of NSF and less likely to damage pedicle. Harvest therefore not compromised by later trauma from resection. Therefore essential pre-op imaging assessment to decide on pathology with large defects or high risk of CSF leak. Disadvantages are if NSF not needed at the end - potential anosmia, scarring may compromise future revision and use of NSF and increased operative time unnecessarily. Nonetheless in this series 86/87 anticipated high flow leaks did have high flow and NSF was used. |
| Cavallo, Luigi M and Solari, Domenico and Somma, Teresa and Savic, Dragan and Cappabianca, Paolo | 2014 | Expanded endoscopic endonasal (anything beyond accessing the sella alone) |  | Awake injection of fibrin glue into the sphenoid cavity for treating the post-op CSF leak (primary repair technique also described) | Primary repair: Tisseal to seal arachnoid. Tutopatch or Tissudura overlay. Lactosorb as semisolid buttress. 6 had turbinate mucoperichondrial free flap. Focus of paper: for persistent CSF leak, in outpatient clinic, while awake, fibrin glue injected into sphenoid cavity (but without filling completely) with spinal needle and in 2 cases, fresh autologous blood injected over site to improve adhesion further. | No need for further GA procedures. Easy quick visualisation of CSF leak site possible with outpatient endoscope. Use of fibrin glue specifically preferred because firstly, biologic product so safer to use nearer cranial contents and secondly, disintegrates quicker so its role is only to temporarily halt CSF leak to allow natural healing to take place |
| Eloy, Jean Anderson and Shukla, Pratik A and Choudhry, Osamah J and Singh, Rahul and Liu, James K | 2012 | Expanded endoscopic endonasal (anything beyond accessing the sella alone) |  | NSF with fascia lata inlay | NSF prepared before resection. Circumferential mucosa denuded (mucocele prevention AND better adhesion). Fascia lata inlay. Implantable acellular dermal allograft next. Finally covered by PNSF. Surgicel around edges of NSF to secure it. Gentamicin soaked gelfoam over NSF. Bacitracin covered merocel nasal tampons inflated with gentamicin solution to buttress. Abx with 3rd gen cephalosporin or penicillin + betalactamase inhibitor for 10 days or until merocel removed. Raised ICP activates avoided. Stool softeners. | NOTE: this is the same described set of techniques for 3rd time from same group. Potentially recurrent patients used (e.g. in previous paper the recurrent osteoblastoma patient is discussed in more detail). |
| Eloy, Jean Anderson and Patel, Smruti K and Shukla, Pratik A and Smith, Mickey L and Choudhry, Osamah J and Liu, James K | 2013 | Expanded endoscopic endonasal (anything beyond accessing the sella alone) |  | Graded repair protocol for EEA | Same technique as in other study by this group (PMID 23070802). New info: NSF harvested contralateral to tumour if possible. Layer 1: autologous fascia lata, layer 2: acellular dermal allograft, layer 3: NSF | Unclear what this paper adds that is different to their previous paper. They highlight this triple layer technique is adequate and does not need lumbar drainage. |
| Liu, James K and Schmidt, Richard F and Choudhry, Osamah J and Shukla, Pratik A and Eloy, Jean Anderson | 2012 | Expanded endoscopic endonasal (anything beyond accessing the sella alone) |  | Multilayer graded approach (defect size and location) to high flow leak in EEA | Fat graft to sellar defect (transsellar approaches), Fascia lata to dura (inlay for transcribe or transclival, but overlay for transplanum as optic exposed, overlay for trans-sellar as much dead space), +- acellular dermal allograft overlay to dura (large transcribifrom defects). Surgicel. +- Repeat fascia lata +surgical if still ioCSFL.. NSF - buttress with Gentamicin-soaked Gelfoam pledgets, Merocel covered in bacitracin ointment x 10-12 days in situ. Antibiotics x 10-12 days, stool softeners, avoid heavy strain/stress | No dural sealant: cost, decreases buttressing force of nasal packs, can be barrier to NSF adherence to tissues. Merocel provides more effective buttress to NSF owing to shape, more comfortable and less likely to be pulled out by mistake. Denude mucosa under overlay graft/NSF to prevent mucocele formation. PNSF is versatile and adaptable (up to approx. 25cm2) for EEA - site must be prepared (e.g. denude mucosa, etc) and buttressed well. |
| Eloy, Jean Anderson and Choudhry, Osamah J and Shukla, Pratik A and Kuperan, Arju-B and Friedel, Mark E and Liu, James K | 2012 | Expanded endoscopic endonasal (anything beyond accessing the sella alone) |  | Multilayer repair with NSF as final common pathway | NSF harvested early. Surrounding mucosa denuded (mucocele prevention and better adhesion). High flow leak converted to low flow by multilayer repair using any of Gelfoam (2) OR autologous fat graft (24) OR autologous fascia lata (13) OR acellular dermal allograft (5) or Duragen (0). NSF then rotated onto this. Surgicel over NSF edges. Then multiple pieces of gentamicin soaked gelfoam pledgets. Expandable merocel nasal tampons covered in bacitracin ointment. 3rd gen cephalosporin or penicillin + beta-lactamase inhibitor for 10days or until tampons removed. Stool softeners. Advice on avoiding raising ICP e.g. leaning forward, blowing nose etc. |  |
| Mortuaire, G and Vandeville, S and Assaker, R and Chevalier, D | 2012 | Expanded endoscopic endonasal (anything beyond accessing the sella alone) |  | Graded repair protocol based on defect location in EEA and CSFR repair post EEA | Ant SB <1cm: Gelfoam or Surgicel. Ant SB <1cm: fat, turbinate mucosa or fascia lata. Rapid rhino balloon packing x2d. Middle SB: fat (to defect and to sphenoid sinus), fascia lata, Tissucol glue. Rapid rhino balloon packing x2d.Bed rest x 2d, PCV vaccine. | Situations at high risk of recurrence concern CSF leaks of the sphenoidal region where local anatomical support and CSF hydrostatic pressure make control of the leak more difficult -> consider multilayer, LD, etc. |
| Garcia-Navarro, Victor and Anand, Vijay K and Schwartz, Theodore H | 2013 | Expanded endoscopic endonasal (anything beyond accessing the sella alone) |  | Use of gasket seal in EEA (with ioCSFL) | Fat (thigh) to pack unless open 3rd Vent, fascia (lata) + vomer or medpor buttress - GASKET, NSF (n=21), duraseal. LD to use IT fluorescein and for post-op drainage. | Ideal repair material is biocompatible, sterile, inexpensive, low donor morbidity, low imaging interference. Gasket seal adv: watertight seal and graft support. Dis: difficult if bony defect is across two planes / across a step. LD: allows sealant to strengthen over first 24-48 hours post-op |
| Patel, Mihir R and Shah, Rupali N and Snyderman, Carl H and Carrau, Ricardo L and Germanwala, Anand V and Kassam, Amin B and Zanation, Adam M | 2010 | Expanded endoscopic endonasal (anything beyond accessing the sella alone) |  | Use of pericranial flap based on supraorbital artery | Pericranial flap made through coronal incision. Flap rotated and inserted via a glabellar incision. Collagen matrix underlay to prevent scarring over brain, pericranial flap over this then in order surgicel -> duragen -> gelfoam -> merocel nasal packing (removed after 3-7days) | Minimal donor site morbidity if harvested using endoscopic technique. Large flap can be provided although from their measurements the extent reached is likely upper clivus/posterior sellar. Is a useful alternative if vascularised flap desired (e.g. if patient requires RT) but NSF not possible (in this cohort most patients had nasal septum invasion). |
| Cappabianca, Paolo and Esposito, Felice and Magro, Francesco and Cavallo, Luigi Maria and Solari, Domenico and Stella, Lucio and de Divitiis, Oreste | 2010 | Expanded endoscopic endonasal (anything beyond accessing the sella alone) |  | Tissue glue to fill dead space | Thin layer fibrin glue on arachnoid surface. Dural substitute with bone substitute in inlay-overlay technique, reinforced by multiple layers of dural substitute, and pedicled nasoseptal flap. | Fibrin glue as sealant and to fill the “dead spaces” |
| Ahn, Jung Yong and Kim, Sun Ho | 2009 | Expanded endoscopic endonasal (anything beyond accessing the sella alone) |  | Group 1: expanded endonasal group. Suturing of fascia using suture-tying microinstruments designed by authors (as primary repair or after post-op leak) | Suture technique (used in 10/16) Fat to fill sella. Fascia (usually fascia lata as more even thickness and larger harvest possible than abdominal fascia) used as inlay. Fascia graft must be >2mm than dural defect. Dural edges need to be carefully preserved if possible for this. Edges then sutured using tools described in paper using 7-0 pronova. First few sutures require graft to be held and needle need not penetrate the full thickness of the graft. Should go through dural edge inside-outside. 12-16 sutures required in authors experience. CONVENTIONAL TECHNIQUE used by authors (used in 6/16) involved reconstruction using additional use of fleece coated fibrin glue (tachochomb), medpor, bone, biolglue or fibrin glue mentioned but not described in detail. | Narrow corridor of endonasal surgery makes reconstructive procedures technically challenging - authors therefore feels suture tying with this equipment makes it less technically challenging. |
| Locatelli, D and Vitali, M and Custodi, V M and Scagnelli, P and Castelnuovo, P and Canevari, F R | 2009 | Expanded endoscopic endonasal (anything beyond accessing the sella alone) |  | Group 1: closure using autologous materials | Use of autologous materials such as septal cartilage, mucoperichondrial, mucoperiosteum and/or abdominal fat, supported by fibrin sealant (Tisseel) and tamponade of the sella with haemostatic materials | Use of autologous materials was this teams old technique. Benefits are that it does not illicit reaction as autologous, cheap and easy to access. However, greater morbidity to patient (more incisions or damage to nasal septum) to obtain materials and longer operative time, increased risk of infection or pain |
| Locatelli, D and Vitali, M and Custodi, V M and Scagnelli, P and Castelnuovo, P and Canevari, F R | 2009 | Expanded endoscopic endonasal (anything beyond accessing the sella alone) |  | Group 2: closure using biomaterials | Use of biomaterials: Tissudura underlay. Tisseel applied. Then Tissuefleece overlay. In 123, Tissudura and tisseel used alone. In 40 intrasellar dead space filled with tissuefleece | Use of synthetic materials is the future according to authors. TissuDura selected as lamellar structure makes it watertight, biochemical makeup promotes haemostasis, easy to manipulate and cut even after hydration, and no post-op rejection evidence found |
| El-Sayed, Ivan H and Roediger, Fredrick C and Goldberg, Andrew N and Parsa, Andrew T and McDermott, Michael W | 2008 | Expanded endoscopic endonasal (anything beyond accessing the sella alone) |  | Use of NSF in EEA | No dural defect: NSF only (n=10), Dural defect: (duragen or fascia or bone) and/or fat, NSF or MTF (n=3), duraseal, gelfoam, foley x 3-5d. Bone graft only for CSF leak repair surgeries. | NSF = robust, easy to raise, versatile - ant, middle, lateral SB. Flap shrinkage in NSF may result in CSFR. Bilateral NSF in those expecting Radiotherapy. MTF useful for small defects when NSF NA (septal or fovea ethmoidalis defects) |
| Kassam, Amin B and Thomas, Ajith and Carrau, Ricardo L and Snyderman, Carl H and Vescan, Allan and Prevedello, Daniel and Mintz, Arlan and Gardner, Paul | 2008 | Expanded endoscopic endonasal (anything beyond accessing the sella alone) |  | Use of NSF in EEA | Collagen matrix +-[fascia secured with stitches or nitinol clips; or acellular dermis; and/or fat (abdominal)], NSF, tissue glue, foley x3-5d. LD if high flow ioCSFL. Avoid stress/strain, stool softeners, Antibiotics until packing removed. NSF usually stored in nasopharynx or (max sinus if target pathology is at nasopharynx or clival). | NSF = versatile (pliable, large arc; anterior, middle, clival, and parasellar), robust, 6-12 weeks until complete mucosalization (vascularised = fast healing). Increased experience = decreased CSFR (six CSF (33%) leaks occurred in our first 25 repairs, whereas we encountered only two postoperative leaks (4%) in the last 50 patients). Disadvantages:: needs to be raised before a posterior septectomy is performed |
| Cavallo, Luigi Maria and Messina, Andrea and Esposito, Felice and de Divitiis, Oreste and Dal Fabbro, Mateus and de Divitiis, Enrico and Cappabianca, Paolo | 2007 | Expanded endoscopic endonasal (anything beyond accessing the sella alone) |  | Glue to fill tissue cavity after EEA | Overlay technique in 12, inlay technique in 3, Inlay-Overlay in 5. Tuteplast (human pericardium)+duragen then lactosorb both overlay in overlay technique, lactosorb inlay in so-called inlay technique, tuteplast inlay, then lactosorb outlay in inlay-overlay technique. Tiseel/bioglue for filling sphenoid sinus. Bed rest 2 days, prophylactic Antibiotics 5 days | Lactosorb is easy to model, resistant and resorbable. In overlay technique, the dural substitute and lactosorb are wedged in the space between dura and bone, forming a "sandwich" which reduces the risk of migration and displacement. |
| Kitano, Masahiko and Taneda, Mamoru | 2007 | Expanded endoscopic endonasal (anything beyond accessing the sella alone) |  | Icing/multilayering (fancy words for multiple layers) of hydroxyapatite Cement for bony defect | Abdo fascia to dura with 5-0 nylon. Initial layer of cement over the fascia. Then multiple layers of cement. Lumbar drain in 7 patients where no water tight closure of dura (average 5.3 days in) | High rate of CSF leak with septal bone or synthetic materials for sellar floor reconstruction. Single layer of cement may not be sufficient, particularly if inadequate dural closure, due to water solubility of materials during setting time. Therefore multiple layers of comment |
| Esposito, Felice and Dusick, Joshua R and Fatemi, Nasrin and Kelly, Daniel F | 2007 | Both approaches described |  | CSF leak grading system creation for graded repair of defects | No leak: haemostatic collagen sponge (Helistat or Instat); Grade 1: +- fat if dead space intrasellar, haemostatic collagen sponge, intrasellar titanium mesh buttress, haemostatic collagen sponge, Cryolife. Grade 2, intrasellar fat grafts, collagen sponge, titanium buttress, fat to sphenoid, cryolife. Grade 3, same as Grade 2 + LD x 2days. Sometimes nasal packing (n=104) | Graded repair protocol means that more than 60% of patients can avoid autologous tissue grafts (G0) and lumbar CSF diversion (G0,1,2). Changes to protocol later in series (cryolife, G1 fat graft if dead space, LD for all G3) = reduced CSFR rate later in series. Glues not a sealant but hold construct together. Titanium buttress = rigid and strong support but equally, may cause damage to neurovascular (rigid, +- sharp edges). Fat is less morbid harvest than muscle or fascia and abdominal is less painful than thigh |
| Hadad, Gustavo and Bassagasteguy, Luis and Carrau, Ricardo L and Mataza, Juan C and Kassam, Amin and Snyderman, Carl H and Mintz, Arlan | 2006 | Expanded endoscopic endonasal (anything beyond accessing the sella alone) |  | Introducing the hadad bassagesteguy flap | collagen matrix inlay, fascia (+nitinol clips) or sellar fat graft onlay, NSF, fibrin glue, foley x 3-5d. Fat to sphenoid too. Avoid stress/strain, stool softeners, open mouth sneezing, Antibiotics until packing removed. NSF: can elevate according to the size/shape needs. | Collagen graft is pliable. Fat graft absorbs CSF pulsations. Nitinol clips helped prevent migration of fascia grafts. NSF has rich vascular pedicle (posterior nasoseptal arteries) = robust/reliable, versatile, easy to elevate, large arc, large size of flap. Disadvantages:: needs to be raised before posterior septectomy (would destroy the pedicle). NSF sharply dec CSFR rates in EEA. |
| Kitano, Masahiko and Taneda, Mamoru | 2004 | Expanded endoscopic endonasal (anything beyond accessing the sella alone) |  | Subdural Patch Graft Technique for watertight dural closure as watertight suturing of the dura mater, although desirable for prevention of CSF leakage, is technically difficult in a narrow deep surgical field such as that in TSS | Post-op lumbar drains in 17/34 | Suturing of dura material for watertight sealing is technically difficult. Patch graft technique may be easier for watertight dural closure. |
| Kitano, Masahiko and Taneda, Mamoru | 2004 | Expanded endoscopic endonasal (anything beyond accessing the sella alone) |  | Subdural Patch Graft Technique for watertight dural closure as watertight suturing of the dura mater, although desirable for prevention of CSF leakage, is technically difficult in a narrow deep surgical field such as that in TSS | Abdo fat packing, then ceramic plate outlay. Lumbar drains in all | Suturing of dura material for watertight sealing is technically difficult. Patch graft technique may be easier for watertight dural closure. |
| Kitano, Masahiko and Taneda, Mamoru | 2004 | Expanded endoscopic endonasal (anything beyond accessing the sella alone) |  | Subdural Patch Graft Technique for watertight dural closure as watertight suturing of the dura mater, although desirable for prevention of CSF leakage, is technically difficult in a narrow deep surgical field such as that in TSS | Fascia lata for dural defect sutured to dura. Lumbar drains in 8/10 | Suturing of dura material for watertight sealing is technically difficult. Patch graft technique may be easier for watertight dural closure. |
| Kitano, Masahiko and Taneda, Mamoru | 2004 | Expanded endoscopic endonasal (anything beyond accessing the sella alone) |  | Subdural Patch Graft Technique for watertight dural closure as watertight suturing of the dura mater, although desirable for prevention of CSF leakage, is technically difficult in a narrow deep surgical field such as that in TSS | Double layer graft using fascia and expanded polytetrafluoroethylene sutured to dura. Lumbar drains in 7/22 | Suturing of dura material for watertight sealing is technically difficult. Patch graft technique may be easier for watertight dural closure. |
| Gode, Sercan and Lieber, Stefan and Nakassa, Ana Carolina Igami and Wang, Eric W and Fernandez-Miranda, Juan C and Gardner, Paul A and Snyderman, Carl H | 2019 | Expanded endoscopic endonasal (anything beyond accessing the sella alone) |  | Use of extracranial pericranial flaps for clival defects (dural) in EEA - when NSF or ITF is not possible (i.e. its 3rd/4th line for clival defects) | Collagen grafts x 2, fascia graft, fat, PCF. LD (n=5/7) | PCF useful if NSF or ITF not possible/failed/necrotic - in the context of CSFL (revision) repair after clival chordoma surgery. Usually use temporoparietal in this context (posterior defects when nasal flaps NA) but its tech difficult, long pedicle, limited size. PCF adv is accessible, easy to dissect, robust pedicle/Q, can be v large in size (can be estimated via CT), low morbidity (incl cosmesis). Extra cranial (ePCF) avoids craniotomy. In posterior high flow regions, grafts / avascular will likely not suffice especially in the context of Radiotherapy. Conclusion: ePCF is an option in clival defects. |
| Kerr, Edward E and Jamshidi, Ali and Carrau, Ricardo L and Campbell, Raewyn G and Filho, Leo F Ditzel and Otto, Bradley A and Prevedello, Daniel M | 2017 | Expanded endoscopic endonasal (anything beyond accessing the sella alone) |  | Indocyanine Green Fluorescence to assess NSF during EEA | ICG given with special endoscope. 4/5 bright and homo. 1/5 distally hetero but proximal homo. MRI afterwards on all cases = enhancement. No CSFL during follow up in all 5 flaps | ICG may help inform the surgeon real-time about NSF viability and the need to use CL NSF or alternative flap if damage suspected intra-op - 'early recognition of arterial compromise, allowing the harvest of alternate flaps or modification of surgery." |
| Park, Woori and Hong, Sang Duk and Nam, Do-Hyun and Kong, Doo-Sik and Ryu, Gwanghui and Kim, Hyo Yeol and Chung, Seung-Kyu and Dhong, Hun-Jong | 2016 | Expanded endoscopic endonasal (anything beyond accessing the sella alone) |  | Use of NSF in primary cases vs cases with a Hx of septal surgery | NSF. Other aspects not mentioned | Previous septal surgery can make NSF technically more difficult (scarring, destroyed pedicles, distorted tissue planes). There was no difference in rate of CSF leakage or flap integrity (MRI with contrast) between the 2 groups. Therefore, NSF for skull base reconstruction is feasible in patients with a history of septal surgery. |
| Ishii, Yudo and Tahara, Shigeyuki and Teramoto, Akira and Morita, Akio | 2014 | Expanded endoscopic endonasal (anything beyond accessing the sella alone) |  | Repair protocol development centred around dural sutures for EEA - small and large defects | Fat (abdominal), direct dural suture, sutured Fascia (lata or abdominal) (inlay) to dura , NSF. 5-0 nylon to dura edges that were already closely apposable, then fascia patch sutured in to gaps. Slip knot technique to slide knots down. | Advocating for suturing of dura to create watertight closure - direct or via fascial patch. Fascial patch means that large dural defects can be repaired watertight. |
| Wessell, Aaron and Singh, Ameet and Litvack, Zachary | 2013 | Expanded endoscopic endonasal (anything beyond accessing the sella alone) |  | One-piece modified gasket-seal closure in EEA (high flow defects) - a unitized porous high-density polyethylene plate/rectus sheath fascia construct without fat grafting | Fascia (abdominal) secured to a bespoke/trimmed medpor by 4 x 4-0 nylon sutures - the fascia is larger SA than the medpor. NSF, Tisseel. LD at 5-10mL x 3-5days. | Traditional gasket - disadvantages: manoeuvring the two components is tech difficult and including op time, needs underlying fat graft to provide fascia graft support (fat graft risks: fat necrosis, visual compression, sinusitis, donor site issues). The construction of the one-piece graft significantly decreases operative time, lowers the learning curve for multi-layered closure (easier to perform) and avoids the need/risks of fat graft. Medpor is easier to cut than vomer/titanium of other gaskets and is elastic (helps fitting into defect) and porous (FV ingrowth). Rectus fascia (vs lata fascia) is thinner, malleable, easier to harvest with minimum donor site morbidity. Tisseal (fibrin) > Duraseal (hydrogel) as hydrogel can prevent NSF from adhering to exposed edges of the fascial graft. |
| Adappa, Nithin D and Learned, Kim O and Palmer, James N and Newman, Jason G and Lee, John Y K | 2012 | Expanded endoscopic endonasal (anything beyond accessing the sella alone) |  | Use of postop MRI (within 48 hours) assessing NSF (to predict failure/CSFR) in "complex" EEA (Kassam grade 3-5) | fat graft, fascia lata graft or Duragen, NSF, tissue glue, nasal sponge packing. +- LD pre-procedure but intra-op, opened D1 post-op and left for x 2-3 days | Post-op MRI for NSF enhancement is not predictive of flap repair |
| Lavigne P, Vega MB, Ahmed OH, Gardner PA, Snyderman CH, Wang EW. | 2020 | Expanded endoscopic endonasal (anything beyond accessing the sella alone) |  | Use of Lateral nasal wall flap for challenging revision EEA defects (2nd line to NSF) | Lateral nasal wall based off the medial branch of the inferior turbinate artery. No other repairs mentioned. | Indications for Lateral nasal wall flap: performed in revision surgery because of NSF unavailability (41.7%), for postoperative CSF leaks secondary to NSF necrosis (41.7%) and for postoperative CSF leaks that required additional vascularized tissue coverage (16.6%). Post op MRI with contrast showed flap enhancement in 21/22 (95.5%). Lateral nasal wall can be raised reactively (naturally lies outside of the surgical field where it is protected from instrument trauma), similarly usually not damaged from previous surgery. Disadvantages:: tech challenge, variable blood supply so needs broad pedicle, smaller SA than NSF and less pliable, length of pedicle makes anterior reconstructions (anterior cranial fossa) tough. |
| Youngerman BE, Kosty JA, Gerges MM, Tabaee A, Kacker A, Anand VK, Schwartz TH. | 2020 | Expanded endoscopic endonasal (anything beyond accessing the sella alone) |  | Acellular dermis vs autologous fascia lata for gasket/button seal in EEA with high flow ioCSFL | Duraform, [AlloMAX (collagen and elastin that is fabricated from human cadaver skin) or fascia (lata) + medpor GASKET] or [button: allomax or fascia lata - if gasket not possible], NSF, duraseal or adheres, floseal, nasopore. LD intra-op but preprocedural for intrathecal fluorescein and drainage. Antibiotics while LD in place. Gasket = 34, Button = 4, NSF = 38 (100%). | The risk of CSF leak was not significantly different in the ADM group compared with the fascia lata group (OR 0.47, 95% CI 0.04–5.70, p = 1.00). Medpor easier to fashion than bone therefore preferable for gasket. acellular dermis avoids donor site complications of f lata (inf, seroma, haematoma, pain -> dec QoL and effects QoL more than endonasal issues). AlloMax promotes ingrowth and vascular into its architecture. Acellular may be a viable option for button/gasket in high flow/risk EEA |
| Xue H, Yang Z, Liu J, Wang X, Bi Z, Liu P. | 2019 | Expanded endoscopic endonasal (anything beyond accessing the sella alone) |  | Continuous dural suturing (with fascia patchwork) in EEA with *high flow* leak and *large defects* | Dural suture group: Fat (thigh) to dead space, artificial dura, +- suturing with fascia (lata) patchwork (5-0 PDS, sliding knot, continuous), fat (thigh), fibrin glue, NSF, iodoform gauze packing x 14d. . Non-dural suture group: Fat (thigh), muscle (thigh) to dead space, artificial dura, collagen sponge, fat, fascia (lata), fibrin glue, NSF, iodoform gauze packing x 14d. iodoform gauze packing x 14d. Antibiotics x 3-5d. LD dep on tumour invasion characteristics, dural defect size x 4-5d | Dural suturing could significantly decrease the incidence of poCSF leakage (p = 0.049, OR 0.108, 95% CI 0.013–0.899). Adv: restore dural integrity, supports surrounding construct and prevents graft shifting and compression of neurovascular structures; potentially graft/LD/NSF sparing. Disadvantages:: time, technical (learning curve). |
| Carnevale C, Tom√°s-Barber√°n M, Til-P√©rez G, Iba√±ez-Dom√≠nguez J, Arancibia-Tagle D, Rodr√≠guez-Villalba R, Sarr√≠a-Echegaray P. | 2019 | Expanded endoscopic endonasal (anything beyond accessing the sella alone) |  | Various techniques for preventing CSFR in EEA with large defects (anterior) | Fat graft or cellulose to dead space (but not if 3rd Vent open). Fascia lata (inlay and onlay layers), Vomer buttress - Gasket seal. Vascular flap: NSF 13, Lat nasal Wall 8, 7 middle turbinate flap. Tisseel. Merocel in latex glove finger + Antibiotics ointment (avoids avulsion of flap on removal) x 5d. Bed rest (head 30deg) x5days, laxatives, fibre diet, Antibiotics. | Gasket and vascular flap complimentary in large defects with high flow. Rigid buttress protects flap from CSF pulse and pressures. Nasal morbidity can be decreased by reconstructing the pedicled nasoseptal flap donor site with a contralateral reverse rotation flap. MTF less nasal morbidity (leaves septum and lateral wall intact), fast healing, less operating times. |
| Leng, Seth Brown, Vijay K Anand, Theodore H Schwartz | 2008 | Expanded endoscopic endonasal (anything beyond accessing the sella alone) |  | Gasket seal technique for EEA (all with ioCSFL) | Fat to sella/cavity (unless 3rd vent open), fascia (lata) OR alloderm - gasket seal with buttress (septal vomer or titanium or Porex), duraseal or fibrin glue. Lumbar drain if CSFL persists despite repair (n=0) or case specific (n=5). | Gasket seal Adv: internal buttress means external support via packing is less important (Avoids foley which is uncomfortable, can be displaced, mucous aspiration, sinus blocking); spares the need for NSF (useful if undergoing irradiation). Disadvantages:: needs bony rim to be placed in, tricky if defect is across multiple planes. The sheet of gasket seal cannot be too thin/flimsy (duragen), the rigid plate of gasket seal needs to be rigid (cartilage not enough) and not reabsorb able (i.e. not medpor) |
| Luginbuhl, Peter G. Campbell MD James Evans MD Marc Rosen | 2010 | Expanded endoscopic endonasal (anything beyond accessing the sella alone) |  | Button technique for EEA (all with high flow ioCSFL) | Button technique to DURAL defect (larger fascia lata, neurolon 40 stitches, small fascia lata), NSF, nasopore, fibrin glue | After employing the button graft to repair the dura on high-flow and open-cisternal cases, the postoperative CSF leak rate declined to 10% (2/20). This statistically significant improvement compares with 45% (9/20) leak rate in the prebutton graft group for open-cistern defects (P < .03). In the button graft group, concomitant nasoseptal flaps were employed in 16/20 repairs with only one CSF leak among this subpopulation. We employed the button technique without a nasoseptal flap in four cases and had one postoperative CSF leak after a craniopharyngioma resections. Adv: button graft spares NSF, fat grafts, implants, problems with graft migration, balloon catheters, or other packing agents. The button graft can be used in conjunction with the nasal septal flap or as a standalone repair with good results reducing the postoperative leak rate to 10% for high-flow CSF repairs. |
| Patel, Priyesh N and Stafford, Alicia M and Patrinely, James R and Smith, Derek K and Turner, Justin H and Russell, Paul T and Weaver, Kyle D and Chambless, Lola B and Chandra, Rakesh K | 2018 | Transsphenoidal (micro or endoscopic) | Endoscopic | Repair protocol using vascularised and non-vascularised techniques | Bone + free mucosa graft (FMG) (57/188), Bone + FMG + fat (49), Dural substitute underlay (19), Dural substitute underlay + fat (14), Dural substitute under + overlay (15), Dural substitute + NSF (22), Dural substitute + NSF + Bone (4), Dural substitute + FMG (5), fat (3) | No rationale given per se. Discussion that in their cohort, NSF patients had higher rates of post-op leaks. Likely due to higher risk patients receiving more aggressive treatment such as the NSF |
| Magro, Elsa and Graillon, Thomas and Lassave, Jerome and Castinetti, Frederic and Boissonneau, Sebastien and Tabouret, Emline and Fuentes, St√©phane and Velly, Lionel and Gras, Regis and Dufour, Henry | 2016 | Transsphenoidal (micro or endoscopic) | Endoscopic | Soft repair vs hard reconstructions. Hard reconstruction had addition of Titanium MESH +/- Medpor. Soft repair details in column AK | NSF in 3. 77 repair with tailored artificial dura (neuropatch) or silastic (dow corning), augmented with pangen or abdominal fat - if leak seen, augmented further with fibrin glue (tissucol) or DuraSeal. 223 titanium mesh + neuropatch +/- medpor | High rates of CSF leak with early soft repairs led to change to hard repair. |
| Slavnic, Dejan and Cook, Richard Floyd and Bahoura, Matthew and Paik, Gijong and Tong, Doris Wl and Houseman, Clifford M and Barrett, Ryan J and Soo, Teck-Mun | 2019 | Transsphenoidal (micro or endoscopic) | Both | Use of Bone Morphogenetic Protein (BMP) - Duragen patch in repair | All duragen samples here are BMP-Duragen samples. First repair: Duragen + duraseal +/- bone/cartilage. Final repair (4th in 2, 2nd in 2 patients) Duragen + BMP +/- duraseal/abdominal fat/fascia Lata/tisseel. Middle repairs: abdominal fat +/- Duragen/duraseal/cartilage/Lumbar drain | BMP found to have osteoinductive properties. Used off-label in spinal procedures to induce osteogenesis. Therefore postulated to do the same in sellar reconstruction. |
| Umamaheswaran, Preethi and Krishnaswamy, Visvanathan and Krishnamurthy, Ganesh and Mohanty, Sanjeev | 2019 | Transsphenoidal (micro or endoscopic) | Both | Graded repair protocol based on intra-op leak | 18 patients underwent type I repair (Surgicel), 21 patients underwent type II repair (Surgicel + Nasoseptal flap ± Fibrin glue), 8 patients underwent type III repair (Surgicel + Abdominal Fat + Fascia lata ± Fibrin glue) and 5 patients underwent type IV repair (Duragen + Nasoseptal flap ± Fibrin glue). All cases packed with polyvinyl acetal sponge (Ivalon) x 2 days. | Unilayer repair for Grade 1 leaks. Multilayer for Grade 2-4 (+- NSF). |
| Fujita, Yuichi and Taniguchi, Masaaki and Tsuzuki, Takashi and Nakai, Tomoaki and Uozumi, Yoichi and Kimura, Hidehito and Kohmura, Eiji | 2019 | Transsphenoidal (micro or endoscopic) | Endoscopic | New liposuction based fat graft | Multilayer: abdominal fat (liposuction fat wrapped in surgicel), suturing the dura 6-0 resorbable PDS, nasal septal cartilage or bone graft, sphenoid sinus mucosal graft, fibrin glue (Beriplast). | Liposuction fat technique has less donor site morbidity but has only been used for lower grade leaks (0, 1, 2) |
| Kim, Eui Hyun and Moon, Ju Hyung and Kim, Sun Ho | 2019 | Transsphenoidal (micro or endoscopic) | Microscopic | Dural Clips | Grade 1 leakage: Clips, +- Suturing (7-0 Pronova),Tachosil. Grade 2-4: clips, fibrin, Avitene. Clips were Commercially available titanium haemostatic microclips measuring only 2.6 mm in length. | Clipping is quicker and less demanding then dural suturing. Avoid if the edges of the normal glandular tissue is thick and if the dural defect is posterior margin of diaphragm, if defect size is large and may need support for 1-2 sutures. |
| Rieley, William and Askari, Ayda and Akagami, Ryojo and Gooderham, Peter A and Swart, Petrus A and Flexman, Ala-M | 2019 | Transsphenoidal (micro or endoscopic) | Endoscopic | Outcomes of CPAP vs no CPAP peri-op in OSA patients (CPAP may adversely effect CSF leak rates and pneumocephalus) | CPAP (anti-supportive) used for patients with OSA. Multilayer repair varied according to intra-op leak and tumour size?. Synthetic with or without fat (n= 313, 75%); mucosal flap (n = 9, 2%); nasoseptal flap (n = 84, 20%); left open (n =10, 2%); and bovine pericardium graft (n =2, 0.5%). |  |
| Farrell, Nyssa Fox and Kingdom, Todd T and Getz, Anne E and Lillehei, Kevin O and Youssef, A Samy and Ramakrishnan, Vijay R | 2019 | Transsphenoidal (micro or endoscopic) | Both | Medpor has a small risk of chronic sphenoid sinusitis and plate extrusion | 52.5% medpor alone, 37.4% medpor + duragen or fibrin glue at dura, 10.1% medpor + fat/fascia/duragen/fibrin glue combo | Medpor is mouldable, porous (promotes tissue ingrowth -> including stability and dec infection), no foreign body reaction. Disadvantages:: radiolucent, infection risk (foreign body) |
| Zhang, Chao and Yang, Ning and Mu, Long and Wu, Chunxiao and Li, Chao and Li, Weiguo and Xu, Shujun and Li, Xingang and Ma, Xiangyu | 2018 | Transsphenoidal (micro or endoscopic) | Endoscopic | Outcomes using NSF | No CSF leak intra-op: "artificial dura", Low flow: "artificial dura", NSF. High flow: fat graft (thigh), fascia lata, NSF, gelatin sponge |  |
| Scagnelli, Robert J and Patel, Varun and Peris-Celda, Maria and Kenning, Tyler J and Pinheiro-Neto, Carlos D | 2019 | Transsphenoidal (micro or endoscopic) | Endoscopic | Free mucosal grafts in TSA / low grade leaks | Pre 2014: Durepair inlay + Surgical onlay. Post 2014: Durepair inlay + Mucosa graft (nasal floor). Durepair inlay + NSF if high flow CSF leak due to opening of suprasellar cistern and third ventricle. | Mucosa grafts give less morbidity than NSF (smell, nasal crusting) and may be more appropriate in TSA / Low grade CSF leaks. However, in EEA and high grade leaks, NSF preferable. |
| Roca, Ele-and Penn, David L and Safain, Mi-G and Burke, William T and Castlen, Joseph P and Laws, Edward R | 2019 | Transsphenoidal (micro or endoscopic) | Both | Fat grafts in TSA and low grade CSF leak | Fat graft divided into small pieces, covered in Avitene, Floseal to seal and MedPor to support, Nasopore packing in posterior aspect of sphenoid sinus. If EEA or high grade leak = add fascia lata graft or NSF. | Fat grafts: autologous and cost-effective coverage of the skull base defect, promotes vascularized granulation tissue, and induces a mild inflammatory response that is helpful for healing. |
| Rasmussen, Jorge and Ruggeri, Carlos and Ciraolo, Carlos and Baccanelli, Matteo and Yampolsky, Claudio and Ajler, Pablo | 2018 | Transsphenoidal (micro or endoscopic) | Endoscopic | Use of L-PRF (leukocyte- and platelet-rich fibrin - from patients blood intra-op) in TSA / Low grade leaks | Grade 0 leak: Spongostan, bone graft (nasal bone) or fat/fascia graft + Tisseal +- Adherus. After these approaches, final layer of L-PRF was placed. Merocel for 2 days always. Post op Antibiotics x 2days. | LPRF induces tissue regen at the recon. Autologous, very low cost, low morbidity to patient, quick to apply |
| Theys, Tom and Van Hoylandt, Anais and Broeckx, Charlotte-Elise and Van Gerven, Laura and Jonkergouw, Joyce and Quirynen, Marc and van Loon, Johannes | 2018 | Transsphenoidal (micro or endoscopic) | Endoscopic | L-PRF in a range of surgeries (incl TSA) - pilot study | L-PRF alone or L-PRF and Tisseal. | Adv: autologous, cheap, induces healing. Disadvantages:: prep fails if interval between blood collection and centrifugation >60 sec. |
| Barger, James and Siow, Matthew and Kader, Michael and Phillips, Katherine and Fatterpekar, Girish and Kleinberg, David and Zagzag, David and Sen, Chandranath and Golfinos, John G and Lebowitz, Richard and Placantonakis, Dimitris G | 2018 | Transsphenoidal (micro or endoscopic) | Endoscopic | Posterior nasoseptal flap (as opposed to traditional flap) in TSA. Flap from ant aspect of middle turb -> sphenoid rostrum posteriorly. Flap created on posterior septal branch of sphenopalatine. | If ioCSF leak: Alloderm inlay, tisseel, posterior NSF + Tisseel + Gelfoam. If no leak: posterior NSF + Tisseel + Gelfoam. Some cases: fat graft (?when/criteria?). All cases: Sphenoid packing with NuGauze sutured x1 suture to the septum, Bacitracin ointment. | No leak = just flap. Leak= multilayer closure. Posterior NSF would be raised and otherwise discarded during procedure anyway so using it can save operative time and decrease patient morbidity (nasal irritation and crusting with large exposure of traditional NSF). However, it likely only has a role in smaller defects. |
| Cong, Zixiang and Liu, Kaidong and Wen, Guodao and Qiao, Liang and Wang, Handong and Ma, Chiyuan | 2018 | Transsphenoidal (micro or endoscopic) | Endoscopic | Sellar floor flap in TSA WITHOUT ioCSF leak. Mucoperiosteal Flap - pedicled. | 43 sellar floor flap (with gelatin sponge under and fibrin glue over), 70 no flap (gel sponge only). | SFF can be quickly integrated into standard sellar floor opening - thus does not add much op time and has less morbidity than other autologous grafts/flaps - would have otherwise been discarded |
| Schuss, Patrick and Hadjiathanasiou, Alexis and Klingm√ºller, Dietrich and G√ºresir, √Ågi and Vatter, Hartmut and G√ºresir, Erdem | 2018 | Transsphenoidal (micro or endoscopic) | Both | Comparison of (1) with muscle patch and fibrin glue and (2) with fibrin glue alone. | Group 1: Fibrin glue, muscle graft +- fascia graft. Group 2: gelfoam and fibrin glue only. All ioCSF leak = LD immediately post-op. All patients = nasal tampon x 2-3 days. | No significant difference gained with using muscle patch. Fibrin glue alone is shorter operating time, dec donor site morbidity, short hospital stay. LD use varied from surgeon to surgeon. |
| Ye, Yuanliang and Wang, Fuyu and Zhou, Tao and Luo, Yi | 2017 | Transsphenoidal (micro or endoscopic) | Endoscopic | Graded repair protocol for eTSA | Graded protocol. Grade 0/1: gelatin sponge or haemostatic gauze, artificial dura overlay, more gelatin sponge or haemostatic gauze. Grade 2: gelatin sponge or haemostatic gauze, artificial dura inlay, more gelatin sponge or haemostatic gauze, another layer of dura overlay, + Cryolife. Grade 3 and/or large dural defect >5mm: gelatin sponge or haemostatic gauze, artificial dura inlay, more gelatin sponge or haemostatic gauze, another layer of dura overlay, Cryolife, NSF. | Artificial dura: provides type I collagen scaffold, induces regeneration of dura and tissue, antibacterial effect, excellent histocompatibility and biodegradability. NSF support for high flow. |
| Jonathan, Gandham E and Sarkar, Sauradeep and Singh, Georgene and Mani, Sunithi and Thomas, Regi and Chacko, Ari George | 2018 | Transsphenoidal (micro or endoscopic) | Endoscopic | io Lumbar Drain insertion - effects on CSF rhinorrhoea | If ioCSF leak: fat graft and LD intraoperative or LD 6 hours post op (depending on whether intraoperative Lumbar Drain group or not for RCT). If no isCSF leak: the LD was removed immediately after extubating in the intraoperative Lumbar Drain group. | Intraoperative CSF drainage significantly reduced the incidence of CSF leak from 46.7% in the no LSAD group to 3.3% in the LSAD group (P < 0.001). However, there were no statistically significant differences in the incidence of postoperative CSF rhinorrhoea between the two groups. There were no major catheter-related complications. There was no statistically significant difference in the extent of resection between the two groups. |
| Nakayama, Noriyuki and Yano, Hirohito and Egashira, Yusuke and Enomoto, Yukiko and Ohe, Naoyuki and Kanemura, Nobuhiro and Kitagawa, Junichi and Iwama, Toru | 2018 | Transsphenoidal (micro or endoscopic) | NS | Use of completely autologous fibrin glue as a repair adjunct. | 400mL blood taken to produce 4.5mL of CAFG. IF ioCSFL: fat graft, oxidized regenerated cellulose + CAFG, lumbar drain | CAFG is totally autologous and decreases risk of infection transmission, allergy. Disadvantage is some patients may not be able to give 400mL blood (anaemia, unstable, bleeding tumour, etc...may need supplements or transfusion) and slightly slower coagulation time (but similar coagulation strength to commercial fibrin glue). Also is less viscous than commercial coagulation glue so may slide down to unwanted areas BUT can soak oxidized regenerated cellulose with it to mitigate. Time consuming process to do in house. |
| Strickland, Ben A and Lucas, Joshua and Harris, Brian-and Kulubya, Edwin and Bakhsheshian, Joshua and Liu, Charles and Wrobel, Boze-and Carmichael, John D and Weiss, Martin and Zada, Gabriel | 2018 | Transsphenoidal (micro or endoscopic) | Both | Graded repair based on ioCSF leak in TSA | If ioCSF leak = Fat (abdominal or thigh) and/or 2-layer fascial apposition method with Fascia (abdominal or thigh). If no io CSF leak = no grafts placed usually but 92 (9%) of patients had this repair without preceding ioCSF leak. In total 57.2% of repairs were fat alone, 18.8% were fat + fascia, 1 case had dural substitute allograft added to fat + fascia. | Half of the patients who developed postoperative CSF rhinorrhoea had no evidence of intraoperative CSF leakage. Unidentified intraoperative CSF leaks and/or delayed development of CSF fistulas are equally important sources of postoperative CSF rhinorrhoea as the lack of employing effective CSF leak repair methods. Empirical sellar reconstruction in the absence of an intraoperative CSF leak may be of benefit following resection of large tumors, especially if the arachnoid is thinned out and herniates into the sella. |
| Zhang, C and Ding, X and Lu, Y and Hu, L and Hu, G | 2017 | Transsphenoidal (micro or endoscopic) | Microscopic | Graded repair based on ioCSF and arachnoid defect in TSA. | Low flow ioCSFL + small arachnoid defect: gelfoam, surgicel, fibrin glue, gelfoam on top to pack. High flow ioCSFL and large arachnoid defect: fat (abdominal), cartilage (septal), artificial dura mater as graft, collagen sponge, and lumbar drain x 2-4 days. | Graded repair protocols are good. |
| Pereira, Erlick A C and Grandidge, Carly A and Nowak, Victoria A and Cudlip, Simon A | 2017 | Transsphenoidal (micro or endoscopic) | Endoscopic | Effect of duraseal for CSFR prevention | Any combination of: duraseal, tisseel, spongostan, fat, floseal. MC was duraseal + spongostan. NSF. | Fat grafts: donor site morbidity, make overpack, can add iso-intense noise to post-op MRI at sella. Fibrin glues (animal/human derived) may allergy, infection, anti-vegan. Duraseal is completely synthetic and resorbable after 4-8 weeks. |
| Thawani, Jayesh P and Ramayya, Ashwin G and Pisapia, Jared M and Abdullah, Kalil G and Lee, John Y-K and Grady, M Sean | 2017 | Transsphenoidal (micro or endoscopic) | Endoscopic | Risk factor analysis (with attention to CSF rhinorrhoea) in TSA for PA | If ioCSF leak: fat abdominal and/or fascia lata, +- NSF, +- LD. CSF leak in fat graft (n 61), fascia lata (n=3) nasoseptal flap (155), or perioperative lumbar drain placement (n 8) - not stat significant difference between groups. | Operative strategies including placement of fat graft, nasoseptal flap, or intraoperative lumbar drain placement may have limited value in reducing the risk of postoperative CSF |
| Sotomayor-Gonz√°lez, Arturo and D√≠az-Mart√≠nez, Armando Jos√© and Radillo-Gil, Ram√≥n and Garc√≠a-Estrada, Everardo and Morales-G√≥mez, Jes√∫s Alberto and Palacios-Ortiz, Isaac Jair and P√©rez-C√°rdenas, Samuel and Arteaga-Trevi√±o, Mauricio and De Le√≥n, √Ångel Mart√≠nez-Ponce | 2017 | Transsphenoidal (micro or endoscopic) | Endoscopic | New NSF (Rescue) technique modification: successful rescue flap via partial posterior superior septectomy (allows enough exposition of the sphenoidal sinus while preserving the nasoseptal septum) | Modified NSF Rescue Flap: . NSF + fibrin glue + Foley Catheter x 3days (Used if ioCSF leak seen [n=6] or if re-operation cases [n=2]). If no ioCSF leak: dural replacement (unspecified), surgicel, gelfoam, fibrin glue. | Rescue flap means the NSF is only raised if needed (therefore may avoid olfactory and crusting issues). Adv of this modification means that the mucosa is manipulated less. Disadvantages:: cant be used if need to expose floor of sphenoid sinus. |
| Zhou, Qiangyi and Yang, Zhijun and Wang, Xingchao and Wang, Zhenmin and Zhao, Chi and Zhang, Shun and Li, Peng and Li, Shiwei and Liu, Pinan | 2017 | Transsphenoidal (micro or endoscopic) | Endoscopic | Risk factors and management of isCSF leak | Grade 0: no repair unless large intrasellar dead space (packed with fat). Grades 1&2: fat graft +-0 artificial dura. Grade 3: fascia lata graft over dura, fat graft, NSF. | Graded repair is important. Intraoperative CSF leaks have a propensity to occur in cases with fibrous or large tumors. For patients with fibrous or large tumors, the NSF should be raised at the beginning of the surgery. |
| Roxbury, Christopher R and Saavedra, Tiffany and Ramanathan, Murugappan and Lim, Michael and Ishii, Masaru and Gallia, Gary L and Reh, Douglas D | 2016 | Transsphenoidal (micro or endoscopic) | Endoscopic | Layered sellar reconstruction with avascular free grafts (i.e. no NSFs) for low flow ioCSF leak in EEA | Structured into dural underlay, dural overlay and bony overlay. Gelfoam or Fat or DuraGen - underlay dura. DuraMatrix or Duragen - overlay dura. Middle turbinate mucosal autograft or Alloderm, tissue glue, gelfoam, surgicel. Nasal packing all cases. MS dura underlay: DuraGen(56/68 [82.4%]). MC dura overlay Duramatrix (62/72, 86%). MC bony overlay: Alloderm (24/73, 32.9%), MT Graft (19/73, 26%), Alloderm + MT Graft combo (8/73, 10.9%). | Multilayer closure is important in achieving water tight seal in the context of ioCSF leak. This closure can spare the need for NSF (therefore avoiding the associated morbidity) in low flow ioCSF leaks. Also this preserves the ability to use a vascularized repair should the patient require a more-complex skull base reconstruction at a later time. There does not seem to be any obvious benefit of the MTMG compared with AlloDerm. Conclusion: a layered, watertight closure with avascular free grafts may be an acceptable alternative for prevention of postoperative CSF leak that is technically simple to perform and is associated with minimal postoperative nasal sequelae (whilst the nasoseptal flap should still be considered the most reliable option for reconstruction of high-flow intraoperative CSF leaks). |
| Fishpool, Samuel J C and Amato-Watkins, Anthony and Hayhurst, Caroline | 2017 | Transsphenoidal (micro or endoscopic) | Endoscopic | free middle turbinate (FMT) graft reconstruction in eTSA | Layers: Gelfoam to dural defect, FMT mucosa graft, Tisseel, Gelfoam to sphenoid sinus. | FMTG for low flow leaks in eTSA. Prevents sinonasal morbidity of NSF and prevents a separate abdominal or thigh incision. Middle turbinate resection improves surgical access but may not be necessary in eTSA... |
| Ismail, Mostafa and Fares, Abd Alla and Abdelhak, Balegh and D'Haens, Jean and Michel, Olaf | 2016 | Transsphenoidal (micro or endoscopic) | Endoscopic | Comparing no packing vs fat or synthetic sellar packing in TSA for PA without ioCSF leak. | Generally: +- packing, bone graft (sphenoid rostrum), fibrin glue. If no ioCSF leak: Group A – with no intrasellar packing (n=16), Group B – with haemostatic materials packing Gelfoam or Surgicel (n=10) , and Group C – with abdominal fat packing (n=21). If ioCSF leak = use fat graft (n=46 and excluded from analysis). | No sellar packing group had no sphenoid sinusitis and empty sella syndrome and there was no stat significant difference in CSF Rhinorrhoea rates. Lack of sellar packing makes intraoperative of post op MRI easier. Consider no packing when no ioCSF leak is present. |
| Amin, Sameh M and Fawzy, Tamer O and Hegazy, Ahmed A | 2016 | Transsphenoidal (micro or endoscopic) | Endoscopic | Composite Vascular Pedicled Middle Turbinate Flap | Fat graft (small sellar defects), MTF (larger sellar defects), NSF (v large defects) | The MTF is suitable for relatively small defects (NSF for larger). Adv: vascular tissue repair, quick, gives mucosa and bony support, no bio adhesive materials or ballooned catheters are needed to fix the flap in place. Disadvantages:: tech demanding. |
| Moon, Ju Hyung and Kim, Eui Hyun and Kim, Sun Ho | 2016 | Transsphenoidal (micro or endoscopic) | Both | New technique for remodelling the redundant arachnoid pouch (the so-called snare technique) to reconstruct the diaphragm, seal off the CSF leak points completely, and reduce the dead space in the tumour resection cavity (large pit adenomas with a lot of suprasellar extension). | 7–0 Pronova suture to snare arachnoid. Avitene - over arachnoid. Tachosil (7 cases) or Medpor (2 cases) - sellar recon. BioGlue over all of this. | Because the surface area of the tumour contacting the arachnoid membrane is relatively large in cases with huge suprasellar extension, the possibility of arachnoid tears during dissection of the tumour from the overlying arachnoid membrane is higher, the leak point of the arachnoid could be larger, and there could be multiple tear points. This type of CSF leakage is difficult to seal off with conventional repair techniques. Snare is less operating time and less puncturing of the arachnoid (as opposed to dural direct suturing). Disadvantages:: apply this technique exclusively for the cases with large redundant arachnoid membranes that herniate downward near or below the level of the dural opening after resection of tumour masses |
| Ozawa, Hiroyuki and Tomita, Toshiki and Watanabe, Yoshihiro and Sekimizu, Mariko and Ito, Fumihiro and Ikari, Yuichi and Saito, Shin and Toda, Masahiro and Ogawa, Kaoru | 2016 | Transsphenoidal (micro or endoscopic) | Endoscopic | Sigmoid incision rescue flap in eTSA as an alternative to NSF (avoid nasal morbidity in selected cases) and rescue (allows wide exposure of the sphenoidal rostrum) | Fat graft x4 (if ioCSL) to pack. Surgicel x3, fascia lata x 1, +- mucous (middle turb) graft x10 - for sellar recon. Pedicled flap (SI rescue x 19, NSF x1) overlay. Sinus Balloon Catheter and chitin-coated gauze for packing. | SI rescue flap: as an alternative to NSF (avoid nasal morbidity in selected cases) and rescue (allows wide exposure of the sphenoidal rostrum) |
| Amano, Kosaku and Hori, Tomokatsu and Kawamata, Takakazu and Okada, Yoshikazu | 2016 | Transsphenoidal (micro or endoscopic) | Endoscopic | Sphenoid sinus mucosa (SSM) for dural defect (in lieu of fat or fascia) - suturing to defect with 6-0 nylon if large defect. OR use pedicle flap of SSM instead of nasoseptal septal flap cover the sellar floor. Harvested mucosa was immersed in saline with antibiotics such as gentamicin before using. Attached with fibrin glue. SSMpatching was applied in 82 cases of which 38 were with suturing, SSM flap in 239, and a combination of them in 24. | SSMpatching was applied in 82 cases of which 38 were with suturing, SSM flap in 239, and a combination of them in 24. | SSM Adv: effective, less invasive, easier for graft harvesting (in the same field of surgery), and providing natural anatomical reconstruction, without potential donor site morbidity - spares fat graft use |
| Sanders-Taylor, Chris and Anaizi, Amjad and Kosty, Jennifer and Zimmer, Lee A and Theodosopoulos, Phillip V | 2015 | Transsphenoidal (micro or endoscopic) | Endoscopic | Is universal sellar recon necessary for eTSA pituitaries | Very small defects - fibrin glue only. Fat (abdominal), cartilage (septum) or bone (septum) buttress, fibrin glue. Nasopore to pack. Avoid heavy stress. Overall, 235 (89%) had reconstruction with autograft (abdominal fat, septal bone/cartilage) and biological glue. | In our patients, delayed CSF leaks likely resulted from missed intraoperative CSF leaks or postoperative changes. Universal sellar reconstruction can pre-emptively treat missed leaks and provide a barrier for postoperative changes. When delayed CSF leaks occurred, sellar reconstruction often allowed for conservative treatment (i.e., lumbar drain) without repeat surgery. We found universal reconstruction provides a low risk of delayed CSF leak with minimal complications. |
| Freyschlag, Christian F and Goerke, Stephanie Alice and Obernauer, Jochen and Kerschbaumer, Johannes and Thom√©, Claudius and Seiz, Marcel | 2016 | Transsphenoidal (micro or endoscopic) | Both | Sandwich technique for TSA (micro and endo) | Sandwich: (spongostan, Tachosil), (Bone buttress - sphenoid sinus wall, septum, vomer), (Tachosil, Spongostan). Nasal tamponades x 1day. LD if ioCSF leak. |  |
| Zhan, Rucai and Chen, Songyu and Xu, Shujun and Liu, James K and Li, Xingang | 2015 | Transsphenoidal (micro or endoscopic) | Endoscopic | Use of LD in low flow ioCSF in eTSA pituitary (in the context of multilayer repair) | Gelfoam fill, synthetic dura overlay, fibrin glue. If low flow ioCSF leak: Gelfoam fill, Fat (thigh), fascia (lata) underlay, synthetic dura overlay, fibrin glue. If high flow ioCSF leak: add NSF, fibrin glue, nasal pledget packing x 1-3 days. Antibiotics x 3-7 days. Bed rest to 15, avoid heavy stress. | Continuous LD may not be necessary for the management of low flow postoperative CSF leak after endoscopic endonasal transsphenoidal approach to pituitary adenoma. |
| Liebelt, Brandon D and Huang, Meng and Baskin, David S | 2015 | Transsphenoidal (micro or endoscopic) | Both | Medpor vs nasal bone buttress | Fat graft (abdominal), medpor or bone (nasal), duraseal or fibrin glue. If ioCSL = LD x 4days. If supra-sellar tumour = LD x 1 day. 136 had medpor, 61 had nasal bone | Always recon the skull base - even if no ioCSF leak, to prevent: chiasmal herniation syndrome or empty sella syndrome, as well as to prevent an arachnoid diverticulum from rupturing after surgery and producing a delayed CSF leak. Medpor = similar CSFR and nasal outcomes, mouldable, no sharp edges, less infection as porous (vs other materials), less nasal donor morbidity (but more expensive than autologous alternatives - nasal bone) |
| Trinh, Victoria T and Duckworth, Edward A M | 2015 | Transsphenoidal (micro or endoscopic) | Endoscopic | Abdominal fat graft+durepair inlay is focus of paper +/- any of the following: durepair overlay, porex graft, duraseal, SMF +/- nasal packing or lumbar drain | All patient received abdominal fat followed by durepair inlay then original dura placed over this then DURASEAL. Additional repairs: 3 durepair onlay. 5 NSF. 1 durepair onlay +NSF. 2 lumbar drains. | Articles main focus is in fact new technique of INTRAumbilical fat harvest to obtain SCARLESS graft |
| Hong, Chang Ki and Kim, Yong Bae and Hong, Je Beom and Lee, Kyu Sung | 2015 | Transsphenoidal (micro or endoscopic) | Both | Repair technique 1 - conventional method - autologous fat graft + fibrin glue +/- lumbar drain | Autologous fat graft with fibrin glue with or without lumbar drainage | This was their conventional repair technique before switching to use of tachosil (see row 85 for second section of this paper) |
| Hong, Chang Ki and Kim, Yong Bae and Hong, Je Beom and Lee, Kyu Sung | 2015 | Transsphenoidal (micro or endoscopic) | Both | Repair technique 2 - tachosil-fibringlue-tachoscil sandwich then medpor on skull base | Tachosil placed over exposed pituitary/diaphragma, sellar dead space then filled with fibrin glue, repeat layer of tachosil over sellar face then medpor used as buttress for final layer | This comparison found tachosil reduced rate of post-op rhinorrhoea, obviated need of lumbar drainage and reduced length of hospital stay. Tachosil pros: no disease risk (as with allograft) or distant site morbidity (as with autograft). No migration. Easily shaped. Easily removed if recurrent surgery required. Flexible for narrow operative corridors. |
| Kim, Eui Hyun and Roh, Tae Hoon and Park, Hun Ho and Moon, Ju Hyung and Hong, Je Beom and Kim, Sun Ho | 2015 | Transsphenoidal (micro or endoscopic) | Endoscopic | Suturing of the anterior pit gland to the anterior dural margin to close a visible defect vs use of tachosil to fill cavity and pull over the exposed anterior dural margin | Conventional repair+sutures vs conventional repair. Sutures are 7-0 pronova to stitch the anterior margin of the pituitary to anterior margin of dura (51 patients). Remaining 71 patients had conventional repair only with Tachosil to fill tumour cavity + haemostatic avitene then further layer Tachosil to cover exposed pituitary and extending anteriorly to cover anterior dural margin + bioglue. In some patients additional use of medpor or macropore to reconstruct sellar floor (no specified numbers) | Addition of sutures was used when a large visible defect anteriorly between pituitary and dural margin was seen to close the gap before carrying out the rest of the repair. Concern from authors that packing alone would widen such a gap. However, article comment highlights that difference in post-op rhinorrhoea between two groups is not significant (but I say to the comment, the group with the sutures did have a visibly larger defect so not comparable) |
| Tewfik MA, Valdes CJ, Zeitouni A, Sirhan D, Di Maio S | 2014 | Transsphenoidal (micro or endoscopic) | Endoscopic | Hemi-transeptal Flap Technique: Adaptation of the Rescue flap. Preserves pedicles early and lifts a flap into nasopharynx, allows wide sphenoidotomy. Creates right sided posterior septum flap. | Hemi-T flap and absorbable stitch for all eTSA. Suggest Rescue or NSF (larger) for EEA. If ioCSF leak, also add 2 layers of fascia lata graft (inlay and onlay). | Hemi-T: shorter op times vs Rescue [152.6 +- 56.8 versus 205.2 +- 61.3 minutes; p ¼ 0.001], similar complications profile (septal perf, cartilage necro, mucosal adhesions) although may be slightly higher, can harvest on a PRN basis (therefore save nasal morbidity if flap not needed). Hemi-T vs Rescue: does not limit surgical access in the same way. |
| Jalessi, Maryam and Sharifi, Guive and Mirfallah Layalestani, Mohammad Rasool and Amintehran, Ebrahim and Yazdanifard, Parin and Rezaee Mirghaed, Omidvar and Farhadi, Mohammad | 2013 | Transsphenoidal (micro or endoscopic) | Endoscopic | Graded repair (ioCSFL based) eTSA Pit Adenoma | Stage I: surgicel, sphenoid sinus filled up with Gelfoam. Stage II: fat graft, surgicel underlay, fascia lata as graft, surgicel, gelfoam to sphenoid sinus. Stage III: As per stage 2, fibrin glue (Glubran 2), +- lumbar drain if high flow ioCSFL Nasal packing unspecified type. Avoid stress/strain. . | Stage I of reconstruction was used in 126 patients (52.5%) with no intra-operative CSF leak or severe prolapse of arachnoid membrane. Stage II was performed in 80 patients (33.3%) with either leak grade 1 (73 patients) or grade 0 with severe prolapse of the suprasellar components induced in the sella (2 cases) or in whom extra-pseudocapsular dissection performed (5 cases). Stage III was performed in 34 cases (14.2%) with either CSF leak grade 2 (29 patients) or grade 1 with simultaneous severe destruction or removal of sellar floor laterally, superiorly or inferiorly (5 patients) which made it impossible to place the fascia underlay to the bone. |
| Brunworth, Joseph and Lin, Ti-and Keschner, David B and Garg, Rohit and Lee, Jivianne T | 2013 | Transsphenoidal (micro or endoscopic) | Endoscopic | Use of NSF for CSFR repair after previous pituitary adenoma TSA | Previously at index surgery - avascular, multilayer closure was performed and failed. During this preoperative repair: salvaged NSF, fat graft (abdominal), fibrin glue, gelfoam. Lumbar drain 10ml/hr x 3/4 days. | Despite prior partial posterior septectomy, an HBF could be harvested by raising the remaining mucoperiosteal paddle between the inferior aspect of the septal perforation and the nasal floor. Although it does not guarantee pedicle viability, endoscopic evaluation of the integrity and dimensions of the remaining mucoperiosteum between the sphenoid rostrum and anterior septum may assist in predicting the feasibility of HBF application. The mean height of the remaining mucoperiosteal tissue between the prior sphenoid sinusotomy and choanae was 14.3 mm, and the mean distance between the most inferior aspect of the septal perforation and nasal floor was 10.7 mm |
| Gaynor, Brandon G and Benveniste, Ronald J and Lieberman, Seth and Casiano, Roy and Morcos, Jacques J | 2013 | Transsphenoidal (micro or endoscopic) | Both | Acellular Dermal Allograft - TSA - Pit Adenoma | No ioCSFL = collagen sponge +- fat graft, Alloderm, bone buttress. ioCSFL: fat or multilayer alloderm (alloderm, gelfoam +- surgicel, alloderm). Always: Merocel x 5-7days with prophylactic Antibiotics cover whilst these were in situ. Sellar floor repair was performed in 259 cases (57.3%) with fat graft (n ¼ 87, 19.2%), AlloDerm (n ¼ 163, 36.1%), materials (n ¼ 9, 2%). | There was no statistically significant difference in postoperative CSF leak rates between AlloDerm and fat graft in the presence of an intraoperative CSF leak (p ¼ 0.34, chi-square test). Allograft = expensive and doesn't save a huge of deal of time vs fat graft, but less donor site morbidity. |
| Banu, Matei A and Kim, Joon-Hyung and Shin, Benjamin J and Woodworth, Graeme F and Anand, Vijay K and Schwartz, Theodore H | 2014 | Transsphenoidal (micro or endoscopic) | Endoscopic | Use of intrathecal fluorescein for known CSFR - to identify leaks and aid repair decision making | General protocol: Abdominal fat inlay, Medpore or vomer, Duraseal +- a vascularized nasoseptal flap. Lumbar drain x 3 days. | In our series, we visualized the fluorescein tinted CSF roughly 80% of the time (91.6 in iatrogenic CSFR). In cases with absent visualization, it is suspected that the fluorescein may have not adequately circulated to the skull base or the skull base breach may have not been actively leaking CSF. Ca n be used for initial leak detection or examining integrity of repair. Interestingly, we achieved successful closure in 7 of 8 patients without fluorescein stained CSF leakage. Thus, visualizing the leak during the repair procedure is useful, but not entirely necessary for a successful closure. disadvantages. ITF: HA, seizure, neurotoxic, LL deficits, opisthotonos. |
| Cavallo, Luigi M and Solari, Domenico and Somma, Teresa and Di Somma, Alberto and Chiaramonte, Carmela and Cappabianca, Paolo | 2013 | Transsphenoidal (micro or endoscopic) | Endoscopic | equine pericardium sheet (LYOMESH®) as dura mater substitute | If no ioCSFL but prolapse of an intact, thinner suprasellar cistern after tumour removal: Lyomesh placed as underlay with fibrin glue. If ioCSFL: lyomesh underlay AND overlay with fibrin glue. | Lyomesh Adv: watertight, biocompatible, no BSE infection risk, transparent, pliable, durable, slow reabsorption & promotes ingrowth of tissue. May spare autologous tissues |
| Shin, Ji-Hyeon and Kang, Seok-Gu and Kim, Sung Won and Hong, Yong Kil and Jeun, Sin-Soo and Kim, Eun Hae and Kim, Soo Whan and Cho, Jin Hee and Park, Yong Jin | 2013 | Transsphenoidal (micro or endoscopic) | Endoscopic | Bilateral NSF elevation. Use of right sided NSF routinely and use of left NSF in cases of confirmed intra-op CSF leak | 14 right NSF used. 13 left NSF used. Multilayer technique. Bilateral NSF raised. Routine part of procedure: vomer/ethmoid bone used as rigid buttress. Next reflected sphenoidal sinus mucosal layer. Next Surgical+Duraseal. Sphenoid sinus filled with nasopore. Then silastic sheets, fixed with 2x 5-0 PDS sutures, then merocel packing. However, if CSF leak confirmed with Valsalva after bone layer, right NSF applied with bony edge overlap and stripping of mucosa off bony edges. Left NSF overlying this is sphenoid sinus mucosa insufficient. | Elevation of NSF after removal of a tumour is often difficult. Use of a single nasoseptal flap might not always seal the entire defect, and the septal mucosa on the contralateral side would be sacrificed anyway. Covering sphenoid sinus with NSF reduces nasal crusting. B/L elevation allows wider field of view. Concomitant septoplasty can be done with no additional incision. Unused flaps can be repositioned back into position |
| Jakimovski, Dejan and Bonci, Gregory and Attia, Moshe and Shao, Huibo and Hofstetter, Christoph and Tsiouris, Apostolos J and Anand, Vijay K and Schwartz, Theodore H | 2014 | Transsphenoidal (micro or endoscopic) | Endoscopic | Main focus of paper is the use of intra-op IT fluorescein to assist with ioCSFL leak detection and therefore guide the need for more aggressive repair with fat +/- NSF | If ioCSFL not detected: gelfoam in sella, floor reconstruction +tissue sealant. If ioCSFL detected: autologous fat in sella, floor reconstruction + tissue sealant. In some patients with tumour>2.5cm, NSF was used (55 patients). The details of remainder of repair are not reported. |  |
| Kim, Boo Young and Shin, Ji-Hyeon and Kang, Seok-Gu and Kim, Sung Won and Hong, Yong Kil and Jeun, Sin-Soo and Kim, Soo Whan and Cho, Jin Hee and Park, Yong Jin | 2013 | Transsphenoidal (micro or endoscopic) | Endoscopic | Bilateral modified nasoseptal rescue flaps elevation | Autologous bone (vomer/PPE) used as rigid buttress to fill defect, sphenoid sinus mucosa covers site, haemostatic agent added next (surgical) then tissue sealant (duraseal). Sphenoid sinus packed with nasopore. If CSF leak detected after autologous bone stage then Right modified rescue NSF was extended anteriorly (to become conventional NSF) and used to cover defect then surgical and duraseal. Denuded sphenoid sinus then covered with left modified rescue NSF. | For small sellar pathology, NSF preparation routinely is deemed too excessive by authors. Hence, they describe technique of modified NSF preparation to facilitate quicker preparation of NSF if CSF leak detected in an initially low leak risk patient. |
| Eloy, Jean Anderson and Shukla, Pratik A and Choudhry, Osamah J and Singh, Rahul and Liu, James K | 2013 | Transsphenoidal (micro or endoscopic) | Endoscopic | Fascia lata onlay with NSF for Transplanum defects | NSF harvested early. Surrounding mucosa denuded (mucocele prevention and better adhesion). High flow leak converted to low flow by multilayer repair using any of Gelfoam (0) OR autologous fat graft (9) OR autologous fascia lata (19) OR acellular dermal allograft (15) or Duragen (2). NSF then rotated onto this. Surgicel over NSF edges. Then multiple pieces of gentamicin soaked gelfoam pledgets. Expandable merocel nasal tampons covered in bacitracin ointment. 3rd gen cephalosporin or penicillin + betalactamase inhibitor for 10days or until tampons removed. Stool softeners. Advice on avoiding raising ICP e.g. leaning forward, blowing nose etc. | Mucosa denuded to prevent delayed mucocele formation. |
| Campos, Francia and Fujio, Shingo and Sugata, Sei and Tokimura, Hiroshi and Hanaya, Ryosuke and Bohara, Manoj and Arita, Kazunori | 2013 | Transsphenoidal (micro or endoscopic) | Microscopic | Use of FGGS-DT (Fibrin Glue-soaked Gelatin Sponge - Diluted) | CSF leakage point blocked with FGGS-DT (1:5 or 1:10 dilution). Sellar further filled with FGGS-DT. Sellar floor reconstructed with vomer. Further 2-3 pieces of FGGS-DT applied and fat then applied in some cases. | Focus of paper is experiment testing tensile strength variation with different thrombin concentrations in making up FGGS and with different setting times. Argument: too strong an adhesive sets too quickly, hindering the reconstruction. And too weak and adhesive does not repair well. Experiment to test optimum adhesion concentration. Optimum concentration 1:10. |
| Berker, Mustafa and Aghayev, Kamran and Y√ºcel, Ta≈ükƒ±n and Hazer, Derya Burcu and Onerci, Metin | 2013 | Transsphenoidal (micro or endoscopic) | Endoscopic | Use of dural flap technique in 50 patients. Technique involves opening dural with a remaining lateral pedicle and closing by compression or with strips of gelatin sponge across defect | 50 patients had dural flap technique. Out of 135 with intra-op leak, 15/135 had dural flap technique+fat+/-fascia lata, 120/135 had fat+/-fascia lata only. Otherwise repairs were as follows: Group 1: defect <5mm OR leak is type 1/2 - free fat+surgical in sella, dural flap technique OR gelatin sponge+surgical as outlay, sphenoid sinus filled with gelatin sponge. Group 2: defect5-10mm OR leak type 3 - free fat+surgical in cavity, Inlay+outlay fascia lata graft, if >15mm defect foley catheter balloon support. Group 3: very large defect >10mm - fascia lata inlay and onlay around dural defect, sellar cavity filled with fat, further 2 layers of fascia lata, foley catheter support and lumbar drainage considered here. | Pros of dural flap technique: native tissue used, maintains natural pedicle for vascular supply, and fewer steps to prepare. They highlight this technique may be limited to small defects which is the case in their cohort. |
| Malik, Muhammad Usman and Aberle, Jens C and Flitsch, Joerg | 2012 | Transsphenoidal (micro or endoscopic) | Microscopic | mTSA CSF leak rates and repair protocol | No ioCSF leak: oxidised cellulose, bone graft (nasal). If ioCSF leak: muscle (lateral rectus) graft + fibrin glue to fill sella in 47 ioCSFL patients (OR duraseal only with no muscle or other glue - 15 io CSFL patients), bone graft (nasal). | DuraSeal useful for ***small*** ioCSFL and may be graft sparing. |
| Peizhi, Zhou and Jianjian, Tang and Ji, Zhang and Youping, Li and Shu, Jiang | 2012 | Transsphenoidal (micro or endoscopic) | Microscopic | Use of titanium clips to diaphragm in mTSA for pituitary adenoma | If no ioCSFL: sponge, dural patch, bone (nasal septum), bioglue. If ioCSFL small defect: As previous. If ioCSFL with moderate dural defect: clip around perforation/defect, sponge, dural patch, bone(nasal septum), bioglue | The occurrence of postoperative CSF rhinorrhoea was significantly lower in group 1 (clip era) than that in group 2 (8/502 vs. 17/228, χ2=14.907, P<.001) but only 57 of 510 group 1 had clips placed. Adv: watertight seal, graft and LD sparing. Disadvantages:: cant do with large defects where dura difficult to approximate, careful with thin/damaged dura, needs specialised clip applicators |
| Eloy, Jean Anderson and Choudhry, Osamah J and Shukla, Pratik A and Kuperan, Arju-B and Friedel, Mark E and Liu, James K | 2012 | Transsphenoidal (micro or endoscopic) | Endoscopic | Multilayer repair with NSF as final common pathway | NSF harvested early. Surrounding mucosa denuded (mucocele prevention and better adhesion). High flow leak converted to low flow by multilayer repair using any of Gelfoam (2) OR autologous fat graft (24) OR autologous fascia lata (13) OR acellular dermal allograft (5) or Duragen (0). NSF then rotated onto this. Surgicel over NSF edges. Then multiple pieces of gentamicin soaked gelfoam pledgets. Expandable merocel nasal tampons covered in bacitracin ointment. 3rd gen cephalosporin or penicillin + betalactamase inhibitor for 10days or until tampons removed. Stool softeners. Advice on avoiding raising ICP e.g. leaning forward, blowing nose etc. |  |
| Mehta, Gautam U and Oldfield, Edward H | 2012 | Transsphenoidal (micro or endoscopic) | Microscopic | Use of ioCSF in mTSA for pituitary adenoma to reduce CSFR | In intraoperative Lumbar Drain group, intraoperative Lumbar Drain under same GA but preprocedural. 20–60 ml of CSF in 20-ml increments is removed via the lumbar catheter (at dural exposure and tumour removal stages). If ioCSL: Fat graft 37 (76% of ioCSFL cases) and intraoperative Lumbar Drain POST procedure in 24 cases (49% of ioCSFL cases). | intraoperative Lumbar Drain use reduces ioCSFL and may spare the amount the repair burden intra-op. The rate of postoperative CSF leakage was similar (5% vs 5%), despite the fact that intraoperative CSF drainage reduced the need for operative repair (from 32% to 5%, p < 0.001). |
| Burkett, Clinton J and Patel, Samip and Tabor, Mark H and Padhya, Tapan and Vale, Fernando L | 2011 | Transsphenoidal (micro or endoscopic) | Both | Fat graft +/- LD vs Duraseal and Duragen - for TSA for pituitary adenoma | Fat graft +- LD group: grades 0 and 1, fibrin sealant and gelfoam; and grade 2, fibrin sealant, gelfoam, abdominal fat graft, nasal packing, and LD for 5 days. For duraseal/gen group: grades 0 and 1, a layer of the Duragen. overlay to dura + Duraseal and grade 2, a layer of Duragen + Duraseal, and nasal packing x 3days. Both groups = 2-3 days Antibiotics. | The failure rate for CSF leak repairs between the two groups was similar. The use of a dural sealant and collagen matrix, however, has the advantages of decreased length of hospital stay, decreased length of Intensive Care Unit stay, avoidance of the morbidity of an extra abdominal incision, and avoidance of the risks of lumbar CSF drainage. Fat grafts can obscure post op MRI and can risk optic compression, donor site morbidity but is cheap and reliable. LD dec CSF pressure, risk infection a(reduced if placed intraoperative sterile environment, pneumocephalus, catheter issues, nerve damage, including length of stay, immobility. Duraseal is totally synthetic - no infectious transmission. Collagen in Duragen is chemotactic for fibroblasts and promotes healing and sealing of dural graft. |
| Kaptain, George J and Kanter, Adam S and Hamilton, David K and Laws, Edward R | 2011 | Transsphenoidal (micro or endoscopic) | Microscopic | Avascular, autologous, repair for mTSA | Fat (abdominal) to sella and sphenoid sinus, septal bone buttress (or if NA, titanium mesh or bioabsorbable polylactide plates). Nasal packs x 2days. LD if supersellar extension (to aid resection) and/or to prevent CSFR. | Avascular autologous repair is effective and acceptable in mTSA. Overall rates of donor site morbidity is low but present (haematoma, visual compression) |
| Cho, Jin Mo and Ahn, Jung Yong and Chang, Jong Hee and Kim, Sun Ho | 2011 | Transsphenoidal (micro or endoscopic) | Microscopic | Use of synthetic tachocomb vs autologous + LD for ioCSF in mTSA. Tachocomb is a haemostatic material used as graft, dural replacement - collagen fleece covered with a layer of human fibrinogen, human thrombin, and bovine aprotinin. | G1: tachocomb to dura +/- avitene, tachocomb to support and cover bony defect, bioglue, G2: coagulated tearing point of dura and placed tachocomb over dura, more tachocomb to support and bony, bioglue. G3: tachocomb in layers to dura, more tacho to support and to bony, bioglue. 90 cases using tachocomb, bioglue (cryolife) | tachocomb: decreases the need for an additional skin incision, reduces patient discomfort, and shortens hospital stay. |
| Kobayashi, Hiroyuki and Asaoka, Katsuyuki and Terasaka, Shunsuke and Murata, Jun-Ich | 2011 | Transsphenoidal (micro or endoscopic) | Endoscopic | Use of titanium clips to dura in eTSA. The AnastoClip Vessel Closure System (VCS) - FDA approved. | VCS clips (2-5 clips), Neoveil (polyglycolic acid nonwoven fabric) or absorbable gelatin sponges (Gelfoam), fibrin glue, fat to sphenoid, LD x 2-3days | Dis: metal artefact on scans, tech difficult, cant be used for large defects (unless clip graft to dura), needs to be used as part of multi-layer closure. Adv: non-penetrating (vs sutures), quicker to apply (vs sutures) |
| Cohen-Gadol, A A and Bellew, M P and Akard, W and Payner, T D | 2010 | Transsphenoidal (micro or endoscopic) | Endoscopic | n -butyl 2-cyanoacrylate (cyanoacrylate) tissue glue for repair of ioCSFL in TSA. | Cyanoacrylate to dura, Fat graft (abdominal) +- Gelfoam or Surgicel to pack sella, cartilage (septal) or bone (sphenoid) buttress. | Adv: very adhesive, solidifies in seconds in the presence of blood, synthetic. Dis: can block suckers, can cause collateral damage owing to adhesion strength |
| Moliterno, Jennifer A and Mubita, Lynn L and Huang, Clark and Boockvar, John A | 2010 | Transsphenoidal (micro or endoscopic) | Endoscopic | Use of high viscosity polymethylmethacrylate cement | Gelfoam additionally placed in subdural space if CSF leak visible. Use of high viscosity polymethylmethacrylate cement injected into epidural space, moulded into shape such that an edge sits between dura and bone. | Cement allows moulding to any shape in skull base defect (therefore better than bone/cartilage as buttress). High-viscosity PMMA vs low viscosity cements reduces rates of leakage of cement or migration down nose while waiting for cement to set. Cement then hardens therefore supporting skull base better than soft grafts. Barium content of PMMA allows guidance under fluoroscopy. Authors state that autologous grafts likely still generally the best and less risk of infection however they posit cement systems as better for larger defects |
| Ahn, Jung Yong and Kim, Sun Ho | 2009 | Transsphenoidal (micro or endoscopic) | Endoscopic | Group 2: standard TSA group. Suturing of fascia using suture-tying microinstruments designed by authors (as primary repair or after post-op leak) | Suture technique used in 5/5 to repair post-op leak. Initially this group had conventional technique. Fat to fill sella. Fascia (usually fascia lata as more even thickness and larger harvest possible than abdominal fascia) used as inlay. Fascia graft must be >2mm than dural defect. Dural edges need to be carefully preserved if possible for this. Edges then sutured using tools described in paper using 7-0 pronova. First few sutures require graft to be held and needle need not penetrate the full thickness of the graft. Should go through dural edge inside-outside. 12-16 sutures required in authors experience. CONVENTIONAL TECHNIQUE used by authors as primary repair in this group involved reconstruction using additional use of fleece coated fibrin glue (tachochomb), medpor, bone, biolglue or fibrin glue mentioned but not described in detail. | Narrow corridor of endonasal surgery makes reconstructive procedures technically challenging - authors therefore feels suture tying with this equipment makes it less technically challenging. |
| Nishioka, Hiroshi and Izawa, Hitoshi and Ikeda, Yukio and Namatame, Hiroaki and Fukami, Shinjiro and Haraoka, Jo | 2009 | Transsphenoidal (micro or endoscopic) | Microscopic | No suture of dural floor before 2005 | If no leak, nothing or surgicel packing. If leak, sellar packing with surgicel covered fat, and then sellar reconstruction with septal bone/cartilage. If major leak, fascial graft | None specific given |
| Nishioka, Hiroshi and Izawa, Hitoshi and Ikeda, Yukio and Namatame, Hiroaki and Fukami, Shinjiro and Haraoka, Jo | 2009 | Transsphenoidal (micro or endoscopic) | Microscopic | Suture of dural floor since 2006 | If no leak, then presumably nothing or surgicel packing as above. If leak, surgicel+fibrin glue for sellar packing, then dural suture (fascial patch if large defect). Sellar floor resorted with bone fragments with surgicel | Narrow dural defect with suturing, as synthetic material are not effective for large defects |
| Rabad√°n, Alejandra T and Hern√°ndez, Diego and Ruggeri, C Santiago | 2009 | Transsphenoidal (micro or endoscopic) | Both | None specific (collagen sponge +/- fat | If no leak: thin layer of glue-soaked sponge for small tumours, packing with soaked sponge for large tumours. If leak, then fat in sellar cavity, thin layer collagen sponge, fat in sphenoid cavity, thin layer sponge. LD post-op | Authors concerns re synthetic material: source of infection, hampers reoperation, expensive. Abdominal incision is minimal and lengthen of operation is lengthened by minutes. |
| El-Banhawy, Omar A and Halaka, Ahmed N and Altuwaijri, Mohammed A and Ayad, Heshmat and El-Sharnoby, Mohamed M | 2008 | Transsphenoidal (micro or endoscopic) | Endoscopic | Nasal Turbinate Graft | Small defects (<5mm) bone overlay only. If >5mm, inlay and overlay bone, sometimes with other grafts (abdominal fat, pedicled mucoperichondrial graft | The manoeuvre is easy to perform with achieving a watertight seal without restriction related to the working space |
| El-Banhawy, Omar A and Halaka, Ahmed N and Altuwaijri, Mohammed A and Ayad, Heshmat and El-Sharnoby, Mohamed M | 2008 | Transsphenoidal (micro or endoscopic) | Endoscopic | Nasal Turbinate Graft | Small defects (<5mm) bone overlay only. If >5mm, inlay and overlay bone, sometimes with other grafts (abdominal fat, pedicled mucoperichondrial graft | The manoeuvre is easy to perform with achieving a watertight seal without restriction related to the working space |
| Yoon, Tae-Mi and Lim, Sang-Chul and Jung, Shin | 2008 | Transsphenoidal (micro or endoscopic) | Both | Mucosal flaps | Sellar packed with fibrin glue-soaked gelfoam and if leak, +abdominal fat. Sellar reconstruction with bone. Covered with sphenoid mucosal flap | Extension dissection of nasal septum can result in complications such as septal perforation and saddle nose. Fat can be a source of infection |
| Yoon, Tae-Mi and Lim, Sang-Chul and Jung, Shin | 2008 | Transsphenoidal (micro or endoscopic) | Both | Mucosal flaps | Sellar packed with fibrin glue-soaked gelfoam and if leak, +abdominal fat. Sellar reconstruction with bone | Extension dissection of nasal septum can result in complications such as septal perforation and saddle nose. Fat can be a source of infection |
| Tamasauskas, Arimantas and Sink≈´nas, Kestutis and Draf, Wolfgang and Deltuva, Vytenis and Matukevicius, Algimantas and Rastenyte, Daiva and Vaitkus, Saulius | 2008 | Transsphenoidal (micro or endoscopic) | Endoscopic | Autologous fat and bone only | Packing sellar and sphenoid with fat, then bone defect repaired with bone | None |
| Tamasauskas, Arimantas and Sink≈´nas, Kestutis and Draf, Wolfgang and Deltuva, Vytenis and Matukevicius, Algimantas and Rastenyte, Daiva and Vaitkus, Saulius | 2008 | Transsphenoidal (micro or endoscopic) | Endoscopic | Synthetic material with autologous fat packing, no autologous bone | INTRADURAL: surgicel on pituitary membrane -> tachosil -> fat -> tachosil. EXTRADURAL (but intrasellar): tachosil, surgicel | None |
| El-Banhawy, Omar A and Halaka, Ahmed N and Ayad, Heshmat and El-Altuwaijri, Mohammed and El-Sharnoby, Mohamed M | 2008 | Transsphenoidal (micro or endoscopic) | Endoscopic |  | For small <5mm defects, simple overlay graft. For >5mm, inlay-overlay grafts. Then gelfoam, then gentamicin soaked ribbon gauze for 48 hours. IV antibiotics for unknown duration | No |
| Sherman, Jonathan H and Pouratian, Nader and Okonkwo, David O and Jane, John A and Laws, Edward R | 2008 | Transsphenoidal (micro or endoscopic) | Both | ePTFE for dural substitute | If CSF leak (15/30) - abdominal fat graft, if no CSF leak (15/30) - gelfoam. Then ePTFE dural substitute as inlay. sellar reconstruction using bone/cartilage (8/30), medpor implant (18) or macropore plate (3), nothing in one case. Lumbar drain only used for injecting air, not for drainage. No post-op drains | ePTFE does not increase risk of infection, unlike non-synthetic material. Not ferromagnetic, therefore less scan interference. Inert, and has good tensile strength, and is pliable |
| Sherman, Jonathan H and Pouratian, Nader and Okonkwo, David O and Jane, John A and Laws, Edward R | 2008 | Transsphenoidal (micro or endoscopic) | Both | ePTFE for dural substitute | If CSF leak (15/30) - abdominal fat graft, if no CSF leak (15/30) - gelfoam. No dural substitute. sellar reconstruction using bone/cartilage (15/30), medpor implant (11/30) or macropore plate (2/30), nothing in two cases. Lumbar drain only used for injecting air, not for drainage. No post-op drains |  |
| Yano, Shigetoshi and Tsuiki, Hiromasa and Kudo, Marei-and Kai, Yutaka and Morioka, Motohiro and Takeshima, Hideo and Yumoto, Eiji and Kuratsu, Jun-ichi | 2007 | Transsphenoidal (micro or endoscopic) | Endoscopic | PGA sheets | PGA sheets and fibrin glue to repair the sellar floor. Fat and LD used in 4 patients with large arachnoid defects | PGA is absorbable, easily shaped and reacts with fibrin glue. Used with success in lung and spinal surgery. |
| Yano, Shigetoshi and Tsuiki, Hiromasa and Kudo, Marei-and Kai, Yutaka and Morioka, Motohiro and Takeshima, Hideo and Yumoto, Eiji and Kuratsu, Jun-ichi | 2007 | Transsphenoidal (micro or endoscopic) | Endoscopic | Use of PGA in skull base repair | Earlier part of series pre-PGA: Mucosal patch +/- abdominal fat and LD for large arachnoid defects. | None specific for mucosa over PGA |
| Dusick, Joshua R and Mattozo, Carlos A and Esposito, Felice and Kelly, Daniel F | 2006 | Transsphenoidal (micro or endoscopic) | Microscopic | Bioglue | Grade 0 - collagen sponge only. Grade 1 - sponge inlay -> mesh intrasellar-extradural -> sponge. Grade 2 - abdominal fat+sponge inlay -> mesh -> abdominal fat + sponge. Grade 3 - abdominal fat+sponge inlay -> mesh -> abdominal fat + sponge with LD 48 hours | None specific given |
| Seda, Lauro and Camara, Rodio Brandao and Cukiert, Arthur and Burattini, Jose Augusto and Mariani, Pedro Paulo | 2006 | Transsphenoidal (micro or endoscopic) | Microscopic | Fibrin glue without grafting or implants | When no CSF leak, surgicel packing only. Nasal cavity packed with Doyle-type cannula | Cellulose helps clot formation and becomes gelatinous, and is haemostatic. Fibrin glue gives good seal and haemostasis |
| Seda, Lauro and Camara, Rodio Brandao and Cukiert, Arthur and Burattini, Jose Augusto and Mariani, Pedro Paulo | 2006 | Transsphenoidal (micro or endoscopic) | Microscopic | Fibrin glue without grafting or implants | With CSF leak, 2 layers of surgicel packing and filling the intrasellar spoace with fibrin glue. Nasal cavity packed with Doyle-type cavity. 5 days of lumbar drainage and prophylactic Antibiotics | Cellulose helps clot formation and becomes gelatinous, and is haemostatic. Fibrin glue gives good seal and haemostasis |
| Silva, L R F and Santos, R P and Zymberg, S T | 2006 | Transsphenoidal (micro or endoscopic) | Microscopic | Secondary repair of CSF fistula | Surgicell->fibrin glue->mucoperiosteum from middle turbinate -> septal cartilage +/- abdominal fat.pABx 24 hours |  |
| Cappabianca, Paolo and Esposito, Felice and Cavallo, Luigi M and Messina, Andrea and Solari, Domenico and di Somma, Lucia G M and de Divitiis, Enrico | 2006 | Transsphenoidal (micro or endoscopic) | Endoscopic | TissuDura ( equine collagen foil) as dural replacement | No other details | Collagen based heterologous grafts avoids need to donor site complications, and does not promote inflammatory response |
| Sade, B and Mohr, G and Frenkiel, S | 2006 | Transsphenoidal (micro or endoscopic) | Microscopic | No fat packing | Inlay of fascia lata/pyophilised bovine pericardium. Then another layer onlay on top of glue. then autologous bone outlay | Fat packing not used due to interference with MRI interpretation, prevent donor site complications, and risk of chiasmal compression. LD reduces risk of CSF rhinorrhoea |
| Sade, B and Mohr, G and Frenkiel, S | 2006 | Transsphenoidal (micro or endoscopic) | Microscopic | No fat packing | Inlay of fascia lata/pyophilised bovine pericardium. Then another layer onlay on top of glue. then autologous bone outlay | Fat packing not used due to interference with MRI interpretation, prevent donor site complications, and risk of chiasmal compression. LD reduces risk of CSF rhinorrhoea |
| Sade, B and Mohr, G and Frenkiel, S | 2006 | Transsphenoidal (micro or endoscopic) | Microscopic | No fat packing | Inlay of fascia lata/lyophilised bovine pericardium. Then another layer onlay on top of glue. then autologous bone outlay | Fat packing not used due to interference with MRI interpretation, prevent donor site complications, and risk of chiasmal compression. LD reduces risk of CSF rhinorrhoea |
| Nishioka, J. Haraoka, and Y. Ikeda | 2005 | Transsphenoidal (micro or endoscopic) | Microscopic | Risk factors for ioCSFL and CSF in mTSA | No leak = surgicel only. Minor leak = surgicel, fat, fibrin glue, cartilage (septum) or bone (septum). Major leak: minor + fascia (lata or abdominal). | recurrent transsphenoidal surgery and radiotherapy as the most important risk factors for intraoperative and as statistically significant risk factors for postoperative CSF leakage |
| van Aken, M O and Feelders, R A and de Marie, S and van de Berge, J H and Dallenga, A H G and Delwel, E J and Poublon, R M L and Romijn, J A and van der Lely, A J and Lamberts, S W J and de Herder, W W | 2004 | Transsphenoidal (micro or endoscopic) | NR | Lumbar drain post-operatively where intra-op leak was observed | Sellar floor reconstruction with bony nasal septum, and sphenoid sinus filled with tissuecoll. Pre-op oral amoxicillin for 1 day, and 6 days post-op, with stat dose intra-op | CSF rhinorrhoea is a risk factor for post-surgical meningitis, therefore lumbar drain to reduce the risk. |
| Sonnenburg, Robert E and White, David and Ewend, Matthew G and Senior, Brent | 2003 | Transsphenoidal (micro or endoscopic) | Endoscopic | No sellar reconstruction | No sellar reconstruction | Avoiding sellar reconstruction avoids donor site complications and interference with post-op tumour reduction measurements |
| Kumar, Arun and Maartens, Nicholas F and Kaye, Andrew H | 2003 | Transsphenoidal (micro or endoscopic) | Microscopic | Bioglue | Abdo fat in cavity -> sphenoid bone reinforced with bioglue. Preop LD if macroadenoma with suprasellar extension, removed if no intraoperative leak. Antibiotics for 2-5 days (mean 2.46) | Bioglue Surgical Adhesive is easy to apply, whilst securing and supporting the bone graft and forms a watertight seal. |
| Cappabianca, Paolo and Cavallo, Luigi Maria and Valente, Vinicio and Romano, Immacolata and D'Enza, Alfonso Iodice and Esposito, Felice and de Divitiis, Enrico | 2004 | Transsphenoidal (micro or endoscopic) | Endoscopic | fibrin sealant and collagen fleece | Sella only repaired if CSF leak, prolapse of cistern or bleeding from cavernous sinus. For sella cavity, fibrin sealant (24) /collagen fleece (16) / sealant+fleece(16). Then polyester/silicone dural substitute | Synthetic material as autologous tissue prolongs the operative time, requires a separate surgical incision, causes additional discomfort, and pore aesthetics. Also autologous tissue usually not available in minimally invasive |
| Cappabianca, Paolo and Cavallo, Luigi Maria and Valente, Vinicio and Romano, Immacolata and D'Enza, Alfonso Iodice and Esposito, Felice and de Divitiis, Enrico | 2004 | Transsphenoidal (micro or endoscopic) | Endoscopic | fibrin sealant and collagen fleece | Sella only repaired if CSF leak, prolapse of cistern or bleeding from cavernous sinus. For sella cavity, collagen fleece (8 cases cisternal prolapse, 1case od bleeding cavernous sinus, 1 bleeding from residual lesion, 6 due to intraoperative CSF leak). Then polyester/silicone dural substitute | Synthetic material as autologous tissue prolongs the operative time, requires a separate surgical incision, causes additional discomfort, and pore aesthetics. Also autologous tissue usually not available in minimally invasive |
| Cappabianca, Paolo and Cavallo, Luigi Maria and Valente, Vinicio and Romano, Immacolata and D'Enza, Alfonso Iodice and Esposito, Felice and de Divitiis, Enrico | 2004 | Transsphenoidal (micro or endoscopic) | Endoscopic | fibrin sealant and collagen fleece | Sella only repaired if CSF leak, prolapse of cistern or bleeding from cavernous sinus. For sella cavity, fibrin sealant (glue used for 6 cases of cisternal prolapse, 2 bleeding cavernous sinus or 16 intraoperative CSF leak. Then polyester/silicone dural substitute | Synthetic material as autologous tissue prolongs the operative time, requires a separate surgical incision, causes additional discomfort, and pore aesthetics. Also autologous tissue usually not available in minimally invasive |
| Cappabianca, Paolo and Cavallo, Luigi Maria and Valente, Vinicio and Romano, Immacolata and D'Enza, Alfonso Iodice and Esposito, Felice and de Divitiis, Enrico | 2004 | Transsphenoidal (micro or endoscopic) | Endoscopic | fibrin sealant and collagen fleece | Sella only repaired if CSF leak, prolapse of cistern or bleeding from cavernous sinus. For sella cavity, sealant+fleece(2 bleeding from cavernous sinus, 1 cisternal prolapse, 13 intraoperative CSF leak). Then polyester/silicone dural substitute | Synthetic material as autologous tissue prolongs the operative time, requires a separate surgical incision, causes additional discomfort, and pore aesthetics. Also autologous tissue usually not available in minimally invasive |
| Cappabianca, Paolo and Cavallo, Luigi Maria and Esposito, Felice and Valente, Vinicio and De Divitiis, Enrico | 2002 | Transsphenoidal (micro or endoscopic) | Endoscopic |  | Dural replacement -> fat/resorbable substances -> dural replacement -> fibrin glue. No closure or packing if no suprasellar extension, or extension that is incompletely removed. Sellar packed if prolapse of cistern, bleeding from medial wall of cavernous sinus or carotid artery injury, CSF (+/- packing sphenoid sinus), pan invasive adenoma (+/- packing sphenoid sinus). | autologous bone or cartilage from the nasal septum usually is unavailable unless it is obtained purposely , therefore synthetic material |
| Kim, J and Cheong, J and Yi, H and Bak, K and Kim, C and Lee, S | 2002 | Transsphenoidal (micro or endoscopic) | Microscopic | Silicone plate for sellar floor reconstruction | Abdo fat in cavity. Silicone plate between bone and dura. Packing with regenerated cellulose if necessary | difficult to harvest a suitable bone splint in many cases, therefore synthetic material used |
| Kelly, D F and Oskouian, R J and Fineman, I | 2001 | Transsphenoidal (micro or endoscopic) | Microscopic | Abdominal fat graft for larger CSF leaks | For larger leaks, abdominal fat inlay, outlay titanium mesh -> abdominal fat. Nasal packing removed in 24 hours | Simplified collagen repair without abdominal fat grafting in one patient with an obvious and relatively large arachnoidal defect resulted in CSF rhinorrhoea. Therefore use of this repair was reserved for only smaller leaks. |
| Kelly, D F and Oskouian, R J and Fineman, I | 2001 | Transsphenoidal (micro or endoscopic) | Microscopic | Collagen Sponge Repair | For small leaks: collagen sponge inlay, outlay titanium mesh-> collagen sponge. Nasal packing removed in 24 hours | Onlay of collagen sponge over dura promotes rapid fibroblast ingrowth, collagen formation, and sealing of dural defects. Collagen sponge is inert, no inflammatory response. No need for tissue grafts, fibrin glue, or postoperative CSF diversion if repair for small leaks successful. |
| Seiler, R W and Mariani, L | 2000 | Transsphenoidal (micro or endoscopic) | Microscopic | Avoiding use of autologous grafts by using: resorbable vicryl patch (polyglactin 910/poly-p-dioxanone, Ethisorb dura patch; Ethicon, Inc., Somerville, NJ) instead of fascia lata; gelatin foam (Spongostan; Johnson & Johnson Medical Ltd., Skipton, UK) for packing the pituitary fossa; and fibrin glue (Tissucol; Baxter AG, Vienna, Austria) for sealing and supporting the sella turcica. | Prophylactic Antibiotics starting pre-op for 24 hours if no lumbar drain. Microadenoma - gelfoam-packing >vicryl patch overlay -> gelfoam. Macroadenoma inlay vicryl patch -> gelfoam, outlay vicryl patch -> foam. If large tumours and large intra-op tear of diaphragmata, then lumbar drain with 2-3 days of Antibiotics. Nasal tampons removed 3rd day | Prevent autograft due to cosmesis. "Cartilage or bone is not used, because the package is held in place and sealed by the fibrin glue." |
| Citardi, M J and Cox, A J and Bucholz, R D | 2000 | Transsphenoidal (micro or endoscopic) | Both | Acellular Dermal Allograft for Sellar Reconstruction after TSA | IV cefazolin 48h starting intraoperative, then to 3-5 days oral 1st-gen cefalosporin. Lumbar drains for macroadenoma with massive sellar expansion and superior erosion, removed 48-72 hours after surgery. Acellular dermal allograft (multiple if empty sellar) -> Bony/cartilaginous septal graft -> acellular dermal allograft -> Mucosal flap over defect glued with fibrin glue. Cavity filled with microfibrillar collagen. | Use of allograft to avoid donor site issues; maintains the architecture of type IV collagen; easy to manipulate; demonstrates host fibroblast and vascular in growth |
| Iannelli, Aldo and Lenzi, Riccardo and Muscatello, Luca | 2014 | Transsphenoidal (micro or endoscopic) | Endoscopic | Adaptation of fat graft technique for eTSA in PA | If macroadenoma or if ioCSFL: Fat (abdominal) wrapped in oxidised cellulose - Tabotamp - which is secured into a ball shape by an absorbable suture. Collagen matrix, fibrin glue. Repairs tested with Valsalva. | Fat wrapping makes the fat easier to manipulate and position (quicker and more reliable placement), stabilises (prevents lobules detaching and unwanted herniation of sections), w |
| Lee, Dong Hoon and Yoon, Tae Mi and Lee, Joon Kyoo and Jang, Woo Youl and Moon, Kyung Sub and Jung, Shin and Joo, Young Eun and Lim, Sang Chul | 2012 | Transsphenoidal (micro or endoscopic) | Endoscopic | Modified traditional NSF for use in revision TSA (when previous large sphenoidotomy/pedicle obliteration has occurred) | Denude mucosa surrounding defect, NSF, fibrin glue, nasal pack. | Modified NSF technique (extending an inferior incision posteriorly toward the nasopharynx, to improve the viability of the NSF = wider base. Removal of rostrum and floor of sphenoid sinus improves pedicle length) possible if previous surgery had large sphenoidotomy. |
| Romero, Alicia Del Carmen Becerra and Nora, Joaquim Ense√±at and Topczewski, Thomaz Eduardo and Aguiar, Paulo Henrique Pires de and Alobid, Isam and Rodrigu√©z, Enrique Ferrer | 2010 | Transsphenoidal (micro or endoscopic) | Endoscopic | Risk factors for CSFR in eTSA - non-graded repair protocol | Dural substitute (Durepair), Tisseel. Fat +- NSF if repair of poCSFR | Durepair is malleable and easy to place. Fat: less donor site morbidity. Unlike other reports, no association between the variables and postoperative CSF fistula was found in this report. |
| Jimenez Zapata HD, Rodriguez Berrocal V, Vior Fern√°ndez C, S√°nchez FM, Garc√≠a Fern√°ndez A. | 2020 | Transsphenoidal (micro or endoscopic) | Endoscopic | Use TachoSil as direct dural/arachnoid repair for ioCSF leak with defect in eTSA for PA | No ioCSFL: surgicel to sella, mucosa (middle turbinate) to dural opening. If ioCSFL (n=24): tachosil to arachnoid, surgicel to sella pack, mucosal graft (or NSF if G2/3 ioCSFL or elevated BMI), surgicel, nasal tampon sponges x 2 days. | Tachosil is cheaper (price/cm) and more adhesive (fibrinogen and thrombin coated) vs. other collagen patches (e.g. Duragen). May provide better seal but is technically challenging to place (needs to have active surface folded when passing through wet nose and used with dry instruments as it sticks to wet surfaces). |
| Xue H, Wang X, Yang Z, Bi Z, Liu P. | 2020 | Transsphenoidal (micro or endoscopic) | Endoscopic | Risk factors for poCSFR in eTSA for PA | No ioCSFL: +- fat (thigh) to dead space, collagen sponges, nasal sponge x3-5d. G1/2: fat, collagen sponge, fibrin glue, iodoform to sphenoid x 2 weeks. G3: fat, fascia lata, NSF, +- LD post-proc x 3-5d. | The presence of ioCSF leak higher grade of ioCSF leak preoperative, Knosp grades 3–4 and visual impairment were predictors for poCSF leak. NSF dec poCSFR |
| Jin B, Wang XS, Huo G, Mou JM, Yang G. | 2020 | Transsphenoidal (micro or endoscopic) | Endoscopic | Use of autologous bone graft for skull base floor to repair the skull base | Acellular dermis (heal-all) as dural replacement, bone graft (sphenoid bone), +- fascia lata (n=60), NSF, Surgicel. Nasopore, iodoform gauze to pack x 10d. Stool softeners, avoid stress/strain, Antibiotics x 3d. Bone graft (not pedicled): floor of sella (Sphenoid) raised like a craniotomy and replaced at the end of the procedure. | Significantly lower leakage rate in the ISBF group than in the bone flap group (6 of 38, 15.8%, P = 0.042). Doing this craniotomy (instead of craniectomy with drill and Kerrison's) similar to transcranial craniotomies with repositioning bone flap in situ-is feasible, safe, and reliable clinically. Facilitates bony healing, safe access if reoperation required, base for overlying NSF. Previous surgery, insufficient pneumatization of the sphenoid sinus, and lesions with potential bone infiltration. (e.g. meningioma) would preclude the use of this technique. |
| Massoud EAS, Hebb ALO, Clarke DB. | 2020 | Transsphenoidal (micro or endoscopic) | Endoscopic | Use of gelfoam packing instead of foley catheter to support NSF in eTSA for PA | If high flow ioCSFL: [Fat (thigh), fascia (lata) - n=35], NSF, fibrin glue, Gelfoam (rolled into cylinders and packed). Septal splints. If low/no flow - NSF only... | Adv: comfort, less likely dislodge, no balloon to burst, adequate buttress |
| Liu B, Wang Y, Zheng T, Liu S, Lv W, Lu D, Chen L, Chen L, Ma T, Gao G, Qu Y, He S. | 2020 | Transsphenoidal (micro or endoscopic) | Endoscopic | Use of intra-op LD in eTSA for GTR and CSFR prevention | Synthetic dura, dural sealant, NSF. If ioCSL: add fascia (lata), some had LD (if in the non-LD group). | Intraoperative LD was associated with a higher rate of GTR (optimize the extent of diaphragm ascent by dec pressure across diaphragm) (92.4% in the LD group vs. 78.6% in the control group, P=0.006), especially in macroadenomas with suprasellar extension - Wilson A->C -(90.3% vs. 75.0%, P=0.012). Both intraoperative and postoperative CSF leak rates were significantly decreased in the LD group (intraoperative: 10.1% vs. 31.4%, P< 0.001; postoperative: 3.4% vs. 11.4%, P=0.035). |
| Jandali D, Shearer S, Byrne R, Papagiannopoulos P, Tajudeen BA, Batra PS. | 2020 | Transsphenoidal (micro or endoscopic) | Endoscopic | Nasal outcomes without sellar repair in eTSA | If no ioCSFL: surgicel, duraseal. | If no ioCSFL and small bony defect (eTSA), CSFR are low but not negligent. The key factor is identifying ioCSFL leak and stepping up repair appropriately (ioCSFL was not seen in this series of CSFR but no Valsalva, IT fluorescein etc is mentioned). |
| Kutlay M, Durmaz O, Kƒ±rƒ±k A, Ya≈üar S, √ñzer ƒ∞, Ezg√º MC, Kural C, Temiz √á, Durmaz A, Daneyemez MK, Izci Y. | 2020 | Transsphenoidal (micro or endoscopic) | Endoscopic | Use of Superior Turbinate Flap after eTSA | If no ioCSFL: Tutopatch or Tutoplast to dura as single inlay layer, bilateral STF, surgicel, tisseel,nasopore or gelfoam packing. If low flow ioCSF/G1 or G2: bilayer (inlay and onlay) Tutopatch or Tutoplast to dura, bilateral STF, surgicel, tisseel, nasopore or gelfoam packing. If high flow ioCSF/G3: bilayer (inlay and onlay) fascia (lata) to dura, surgicel, bilateral STF, surgicel, tisseel, nasopore or gelfoam packing, merocel soaked in bacitracin x 5-7d. If high flow: add acetazolamide. If G2 or G3: LD post-proc intra-op. All cases: Antibiotics x 5 days, avoid stress/strain, stool softeners | Dis: *variable anatomy*, have to alter procedure (STs often sacrificed), small flap so need bilateral, short, limited arc of rotation (sellar repair only). Adv: preserves NSF or can be used for unexpected ioCSFL when NSF hasn't been raised - i.e. as a rescue flap; low nasal morbidity |
| Eichberg DG, Richardson AM, Brusko GD, Ali SC, Buttrick SS, Shah AH, Alam ES, Sargi ZB, Komotar RJ. | 2019 | Transsphenoidal (micro or endoscopic) | Endoscopic | Use of Dehydrated amniotic membrane (DAM) in eTSA for PA | Surgicel to sella, Dehydrated amniotic membrane (Cygnus Solo) to dura, Alloderm, gelfoam wrapped in surgicel, merocel. If high flow CSFL = add NSF. Repair tested with Valsalva | Dehydrated amniotic membrane (DAM) allograft: augments epithelialization + facilitates wound healing (growth factors); and minimizes and impedes bacterial growth (antimicrobial properties); donor site sparing, does not give FB reaction like synthetics might (low expression of cell surface antigens so does not trigger strong immune response), adequate safety profile with no adverse reactions directly related to the DAM product. |
| Zeden JP, Baldauf J, Schroeder HWS. | 2020 | Transsphenoidal (micro or endoscopic) | Endoscopic | Use of PDS foils for sellar floor repair in eTSA for PA | Gelatin foam (no CSF leak, thick diaphragm), collagen matrix (low flow ioCSFL, thin diaphragm), oxidised cellulose OR fat (abdominal) (G2/3 ioCSFL) to sella. Fibrin glue. PDS foil to sella floor | PDS as a buttress. Adv: minimal radio artefact, tech easy, cheap, effective, rigid but pliable (can withstand CSF pressure changes and stabilise construct). Needs enough bony margin of the sellar floor left that enables a sufficient fixation of the foil. PDS foil: pliable, trimmable, absorbable, inert, MRI compatible, fast, inexpensive. Used to provide support to the soft packing material and prevent dislodgement. Used when there is a bony ridge that it can be overlapped with (sellar pack -> PDS -> bony defect) |
| Zanation, Adam M and Carrau, Ricardo L and Snyderman, Carl H and Germanwala, Anand V and Gardner, Paul A and Prevedello, Daniel M and Kassam, Amin B | 2009 | Expanded endoscopic endonasal (anything beyond accessing the sella alone) |  | Use of nasoseptal flap | Dura Matrix or Duragen, +- fat or Alloderm, NSF (mucosa is drilled away over the bony areas of the nasoseptal flap onlay to ensure healing), Surgicel, DuraSeal, Gelfoam - to prevent the Foley balloon or tampon sponge packing from sticking to the flap. If the defect is primarily sellar or parasellar, then a 10-mL Foley balloon is used as the bolster. If the defect is cribriform or clival, then usually tampon sponges are used. For high-flow leaks these stay in place for 5–7 days. Antibiotic while the tampon sponges are in place. LD for almost all (post-op). | The overall postoperative CSF leak rate was 5.7% (4/70). All four postoperative leaks were successfully managed with endoscopic repair, fat bolstering, and repeat CSF lumbar drain diversion. Paediatric patients (2/6, 33%, p=0.002 - possible flap size limitation in paediatric skull base reconstructions), large dural defects (CSFR 4/42, 9.5%, p=0.14) , and radiation therapy (CSFR: 2/12, 12.5%, p=0.07) were noted to be factors in reconstructive failure |
