# Supplementary material for: Skull base repair following endonasal pituitary and skull base tumour resection: a systematic review
Source: Pituitary. 2021 May 10;24(5):698–713. doi: 10.1007/s11102-021-01145-4 (PMC8416859; doi:10.1007/s11102-021-01145-4)
Supplement: Supplementary file 5 — Supplementary file5 (DOCX 21 kb) Supplementary information 5: Taxonomy of repair techniques with the number of studies reporting the use of the repair technique. [file 11102_2021_1145_MOESM5_ESM.docx]

Supplementary Information 4: Taxonomy of repair techniques with the number of studies reporting the use of the repair technique. TSA: transsphenoidal approach, EEA: extended endonasal approach, NSF: nasoseptal flap.

| **Category** | **Sub-category 1** | **Sub-category 2** | | **Examples** | **TSA**  **(95 studies)** | **EEA**  **(55 studies)** | **Both**  **(43 studies)** |
| --- | --- | --- | --- | --- | --- | --- | --- |
| Dural repair | Direct closure | | Sutures +- patchwork | Sutures: Nylon, Prolene  Patches: Fascia, collagen sponge/fleece | Sutures - 8  Fascia - 9  Collagen- 4 | Sutures - 25  Fascia - 19  Collagen- 1 | Sutures - 3  Fascia - 9  Collagen - 1 |
|  |  | | Clips | Nitinol, titanium | 3 | 3 | 1 |
|  | Reconstruction | | Autologous | Fascia lata, rectus fascia | 8 | 14 | 9 |
|  |  | | Synthetic | Duramatrix, duragen, durepair, alloderm ReDura (nanoporous dura synthetic), Tissudura, expanded polytetrafluoroethylene dural substitute, | 23 | 21 | 9 |
|  |  | | Allograft: | Cadaver, Dehydrated amniotic membrane | 1 | 4 | 0 |
|  |  | | Xenograft | Lysomesh (equine), Lyoplant (bovine), Tutopatch (equine) | 5 | 1 | 0 |
| Barrier-restoring grafts | Autologous | | Fascia | Fascia lata, rectus fascia | 28 | 31 | 24 |
|  |  | | Mucosa | Septal, turbinates, sphenoid | 13 | 4 | 8 |
|  |  | | Cartilage | Septal | 6 | 1 | 0 |
|  |  | | Bone | Vomer, septal, turbinate | 13 | 4 | 1 |
|  | Allografts | |  | Cadaveric fascia lata, Allomax, acellular dermal allograft | 6 | 7 | 6 |
|  | Xenografts | |  | Lysomesh (equine pericardium)) | 4 | 0 | 0 |
|  | Synthetic | | Absorbable | Gelfoam | 1 | 4 | 5 |
|  |  | | Haemostatic | Surgicel, Tachocomb | 3 | 1 | 1 |
|  |  | | Cement | Hydroxyapatite | 5 | 0 | 1 |
|  | Button technique | |  | Fascia lata, nylon sutures | 0 | 2 | 0 |
|  | Gasket Seal | |  | Graft: fascia lata, Tachosil  Buttress: vomer, Medpor, titanium mesh | 0 | 10 | 10 |
| Volume-restoring grafts | Autologous | | Fat grafts | Abdo, thigh, femoral/inguinal | 67 | 33 | 37 |
|  |  | | Muscle | Quadriceps, lateral rectus | 2 | 1 | 0 |
|  | Synthetic | |  | Collagen sponge, gelatin sponge | 13 | 15 | 14 |
| Pedicled vascular flaps: | Nasal | | NSF | Hadad-Bassagasteguy, rescue, modified rescue, extended + Janus/bilateral | NSF -27  Rescue - 2  Modified rescue - 2 | NSF – 40  Janus - 1 | NSF – 29 |
|  |  | | Other | Inferior turbinate, middle turbinate, superior turbinate, Sellar floor, lateral nasal, inverted rhinopharyngeal flap | 4 | 8 | 2 |
|  | Extra-nasal | |  | Pericranial, temporoparietal, buccinator, palatal, occipital, radial forearm (free flap) | 0 | 2 | 4 |
| Haemostatic | Fluid-based | |  | Surgiflo, Floseal | 0 | 1 | 0 |
|  | Membrane-based | |  | Surgicel, Avitene, Tachocomb, Helistat or Instat | 72 | 11 | 13 |
| Tissue glues | Fibrin-based | |  | Autologous fibrin sealant, Tisseel | 50 | 14 | 13 |
|  | Synthetic | |  | Polyethylene glycol (Duraseal), hydrogel (adherus) | 11 | 10 | 10 |
|  | Other | |  | Glutaraldehyde (Bioglue), cyanoacrylate | 16 | 13 | 2 |
| Supportive: Construct stabilization | Buttress | | Autologous | Bone, Cartilage | 28 | 10 | 10 |
|  |  | | Synthetic | Absorbable: Medpor, Lactosorb, Polydioxanone  Non-absorbable: Titanium mesh | 27 | 8 | 11 |
|  | Packing | | Balloon | Spherical shape: Foley, Cylindrical shape: Merocel, Pope pack, Rapid rhino balloon | 9 | 25 | 11 |
|  |  | | Non-balloon | Bismuth-soaked ribbon gauze, iodoform gauze, polyvinyl alcohol sponge, Nasopore (Absorbable), Gelfoam packing | 37 | 7 | 7 |
| Supportive: CSF diversion | Short term | |  | Lumbar puncture, lumbar drain, external ventricular drain | 73 | 42 | 31 |
|  | Medium/Long term | |  | Ventriculoperitoneal shunt, lumbar shunt | 3 | 3 | 1 |
| Supportive: Other | Nasal trumpets | |  |  | 0 | 2 | 0 |
